# Supplementary material for: Construct ceRNA Network and Risk Model of Breast Cancer Using Machine Learning Methods under the Mechanism of Cuproptosis
Source: Diagnostics (Basel). 2023 Mar 22;13(6):1203. doi: 10.3390/diagnostics13061203 (PMC10047351; doi:10.3390/diagnostics13061203)
Supplement: Supplementary file 1 [file diagnostics-13-01203-s001.zip › Figure S2/ggalluvial.pdf]

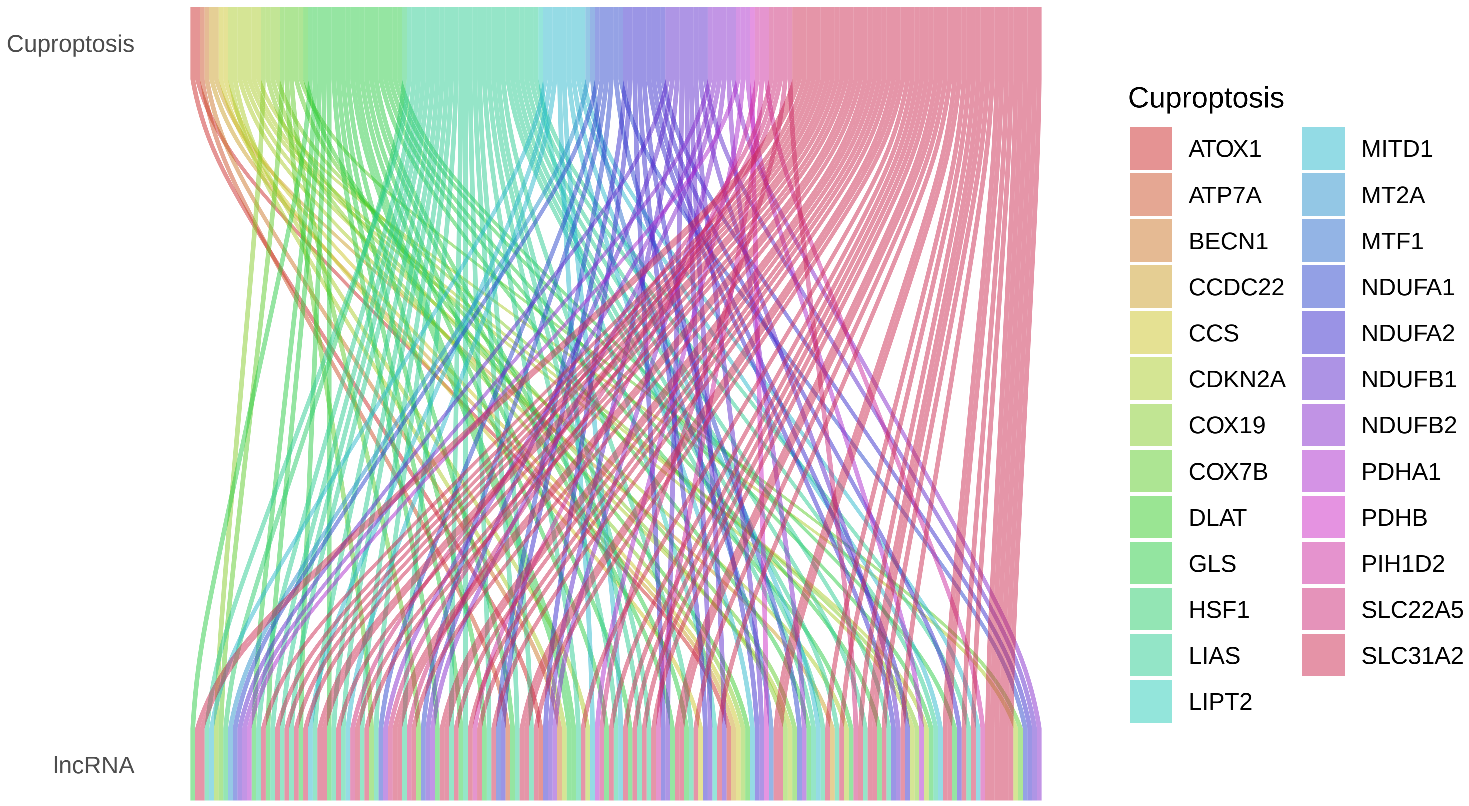
Cuproptosis
lncRNA
Cuproptosis
ATOX1
ATP7A
BECN1
CCDC22
CCS
CDKN2A
COX19
COX7B
DLAT
GLS
HSF1
LIAS
LIPT2
MITD1
MT2A
MTF1
NDUFA1
NDUFA2
NDUFB1
NDUFB2
PDHA1
PDHB
PIH1D2
SLC22A5
SLC31A2
