# Supplementary material for: Construct ceRNA Network and Risk Model of Breast Cancer Using Machine Learning Methods under the Mechanism of Cuproptosis
Source: Diagnostics (Basel). 2023 Mar 22;13(6):1203. doi: 10.3390/diagnostics13061203 (PMC10047351; doi:10.3390/diagnostics13061203)
Supplement: Supplementary file 1 [file diagnostics-13-01203-s001.zip › Introduction of Supplementary Materials.pdf]

Introduction of Figure:

Figure S1. (A) The diagnostic performance these three hub genes used as breast cancer diagnostic genes. (B) The ROC of the 1-, 3-, and 5-year OS for test set samples.

Figure S2. Visualization of co-expression analysis of CRGs and CRLs.

Figure S3. The correlation between 50 medicines and risk score, as well as the differential expression of these medicines in the high - and low-risk group.

Introduction of Table:

Table S1. Source of datasets and related information.

Table S2. Classification and description of references.

Table S3. Four CRLs and their corresponding regression coefficients.

Table S4. Compared with other prognostic models constructed using CRLs.
