# Supplementary material for: Construct ceRNA Network and Risk Model of Breast Cancer Using Machine Learning Methods under the Mechanism of Cuproptosis
Source: Diagnostics (Basel). 2023 Mar 22;13(6):1203. doi: 10.3390/diagnostics13061203 (PMC10047351; doi:10.3390/diagnostics13061203)
Supplement: Supplementary file 1 [file diagnostics-13-01203-s001.zip › Table S1.pdf]

Table S1 Source of datasets and related information

Datasets	Source	Platform	Sample	Normal	Tumor
GSE54002	GEO	GPL570	breast	16	417
GSE29431	GEO	GPL570	breast	12	54
GSE42568	GEO	GPL570	breast	17	104
GSE139038	GEO	GPL27630	breast	24	41
GSE205185	GEO	GPL21185	breast	5	17
