# Supplementary material for: Construct ceRNA Network and Risk Model of Breast Cancer Using Machine Learning Methods under the Mechanism of Cuproptosis
Source: Diagnostics (Basel). 2023 Mar 22;13(6):1203. doi: 10.3390/diagnostics13061203 (PMC10047351; doi:10.3390/diagnostics13061203)
Supplement: Supplementary file 1 [file diagnostics-13-01203-s001.zip › Table S2.pdf]

Table S2 Classification and description of references

References	Description
[13-14]	There are only two published papers on breast cancer in which cuproptosis-related lncRNAs were used to construct prognostic models. It shows that our risk model has certain novelty.
[20-26]	Previous studies were used to prove the role of CRCGs screened in breast cancer.
[27]	The functions and pathways of 14 CRCGs are mainly enriched in breast cancer.
[28-31]	To prove the significance of the results of immune infiltration analysis on the occurrence and development of breast cancer.
[33-39]	Citing previous studies, the important role of our ceRNA network in breast cancer was expounded.
[40-41]	The real-life use cases of the risk prognosis model
[42-48]	Of the 50 potentially sensitive drugs, examples include drugs that have been reported to have some effect on breast cancer.
