# Supplementary material for: Construct ceRNA Network and Risk Model of Breast Cancer Using Machine Learning Methods under the Mechanism of Cuproptosis
Source: Diagnostics (Basel). 2023 Mar 22;13(6):1203. doi: 10.3390/diagnostics13061203 (PMC10047351; doi:10.3390/diagnostics13061203)
Supplement: Supplementary file 1 [file diagnostics-13-01203-s001.zip › Table S3.pdf]

Table S3 Four CRLs and their corresponding regression coefficients

CRL	regression coefficient
C9orf163	1.8365139936957
PHC2-AS1	2.29853199668974
AC087741.1	0.950421008877884
AL109824.1	0.601632220939569
