# Supplementary material for: Construct ceRNA Network and Risk Model of Breast Cancer Using Machine Learning Methods under the Mechanism of Cuproptosis
Source: Diagnostics (Basel). 2023 Mar 22;13(6):1203. doi: 10.3390/diagnostics13061203 (PMC10047351; doi:10.3390/diagnostics13061203)
Supplement: Supplementary file 1 [file diagnostics-13-01203-s001.zip › Table S4.docx]

Table S3 Compared with other prognostic models constructed using CRLs

| References | Data source | The number of CRLs used to build the model | The AUC of ROC for all samples | The AUC of ROC for training samples | The AUC of ROC for test samples |
| --- | --- | --- | --- | --- | --- |
| [13] | TCGA | 11 | AUC at 1 year: 0.766 | AUC at 1 year: 0.766 | AUC at 1 year: 0.686 |
|  |  |  | AUC at 3 years: 0.734 | AUC at 3 years: 0.734 | AUC at 3 years: 0.687 |
|  |  |  | AUC at 5 years: 0.736 | AUC at 5 years: 0.736 | AUC at 5 years: 0.686 |
| [14] | TCGA | 37 | AUC at 1 year: 0.766 | - | - |
|  |  |  | AUC at 3 years: 0.808 | - | - |
|  |  |  | AUC at 5 years: 0.745 | - | - |
| This study | TCGA | 4 | AUC at 1 year: 0.721 | AUC at 1 year: 0.740 | AUC at 1 year: 0.697 |
|  |  |  | AUC at 3 years: 0.695 | AUC at 3 years: 0.776 | AUC at 3 years: 0.604 |
|  |  |  | AUC at 5 years: 0.633 | AUC at 5 years: 0.715 | AUC at 5 years: 0.550 |

Notes：The '-' in the table indicates that no experimental data was obtained in the corresponding study.
