# Supplementary material for: Construct ceRNA Network and Risk Model of Breast Cancer Using Machine Learning Methods under the Mechanism of Cuproptosis
Source: Diagnostics (Basel). 2023 Mar 22;13(6):1203. doi: 10.3390/diagnostics13061203 (PMC10047351; doi:10.3390/diagnostics13061203)
Supplement: Supplementary file 1 [file diagnostics-13-01203-s001.zip › Table S4.pdf]

Table S3 Compared with other prognostic models constructed using CRLs

References	Data source	The number of CRLs used to build the model	The AUC of ROC for all samples	The AUC of ROC for training samples	The AUC of ROC for test samples
[13]	TCGA	11	AUC at 1 year: 0.766	AUC at 1 year: 0.766	AUC at 1 year: 0.686
			AUC at 3 years: 0.734	AUC at 3 years: 0.734	AUC at 3 years: 0.687
			AUC at 5 years: 0.736	AUC at 5 years: 0.736	AUC at 5 years: 0.686
[14]	TCGA	37	AUC at 1 year: 0.766	-	-
			AUC at 3 years: 0.808	-	-
			AUC at 5 years: 0.745	-	-
This study	TCGA	4	AUC at 1 year: 0.721	AUC at 1 year: 0.740	AUC at 1 year: 0.697
			AUC at 3 years: 0.695	AUC at 3 years: 0.776	AUC at 3 years: 0.604
			AUC at 5 years: 0.633	AUC at 5 years: 0.715	AUC at 5 years: 0.550

Notes：The '-' in the table indicates that no experimental data was obtained in the corresponding study.
