# Supplementary figures and images for: Construct ceRNA Network and Risk Model of Breast Cancer Using Machine Learning Methods under the Mechanism of Cuproptosis
Source: Diagnostics (Basel). 2023 Mar 22;13(6):1203. doi: 10.3390/diagnostics13061203 (PMC10047351; doi:10.3390/diagnostics13061203)

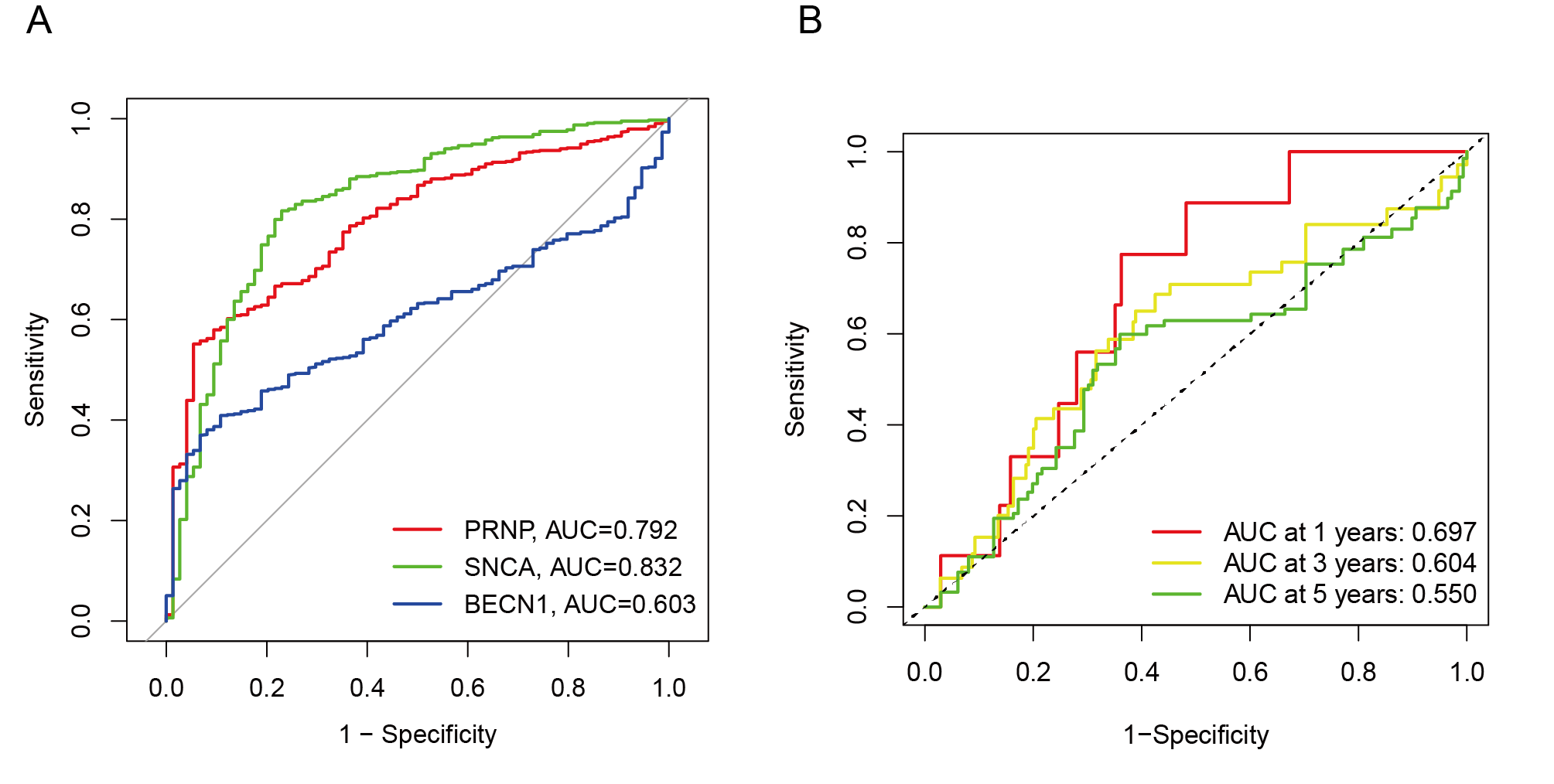

Supplement: Supplementary file 1 [file diagnostics-13-01203-s001.zip › Figure S1/Figure S1.png]

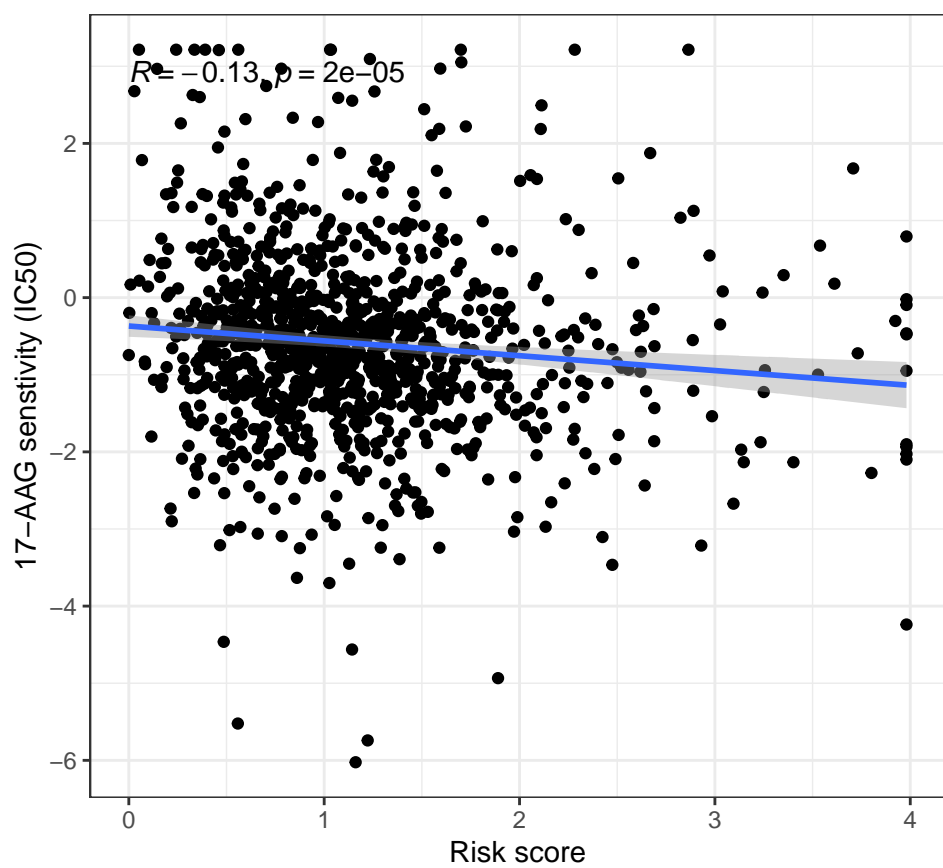

Supplement: Supplementary file 1 [file diagnostics-13-01203-s001.zip › Figure S3/Cor.17-AAG.pdf]

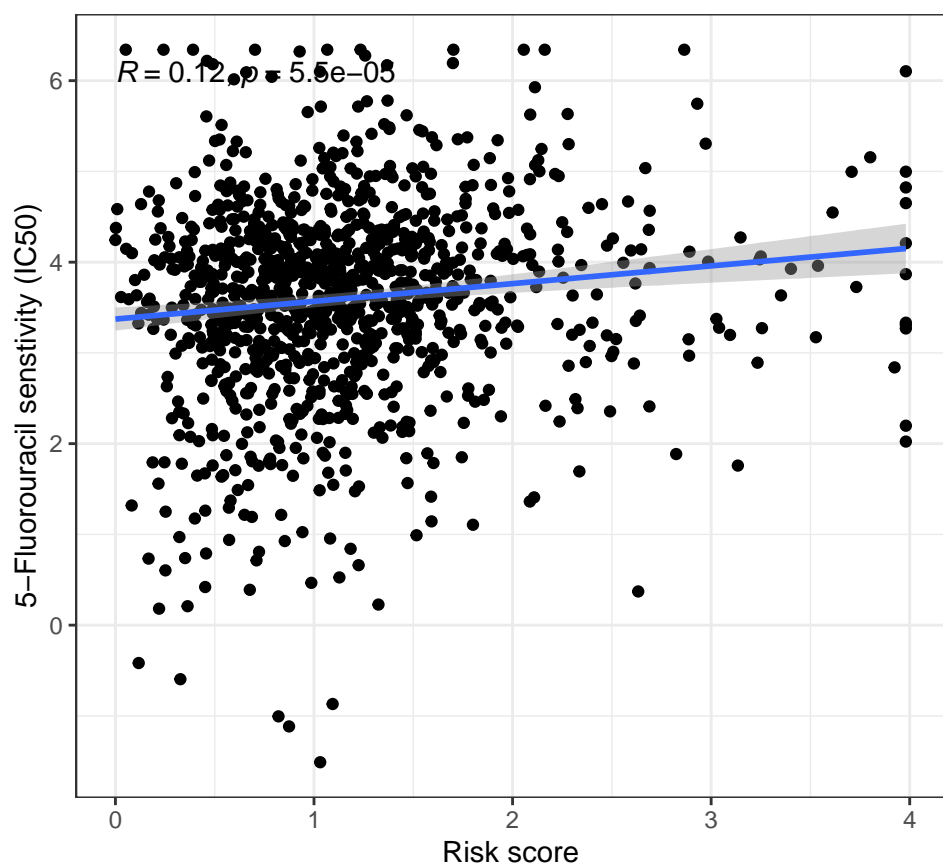

Supplement: Supplementary file 1 [file diagnostics-13-01203-s001.zip › Figure S3/Cor.5-Fluorouracil.pdf]

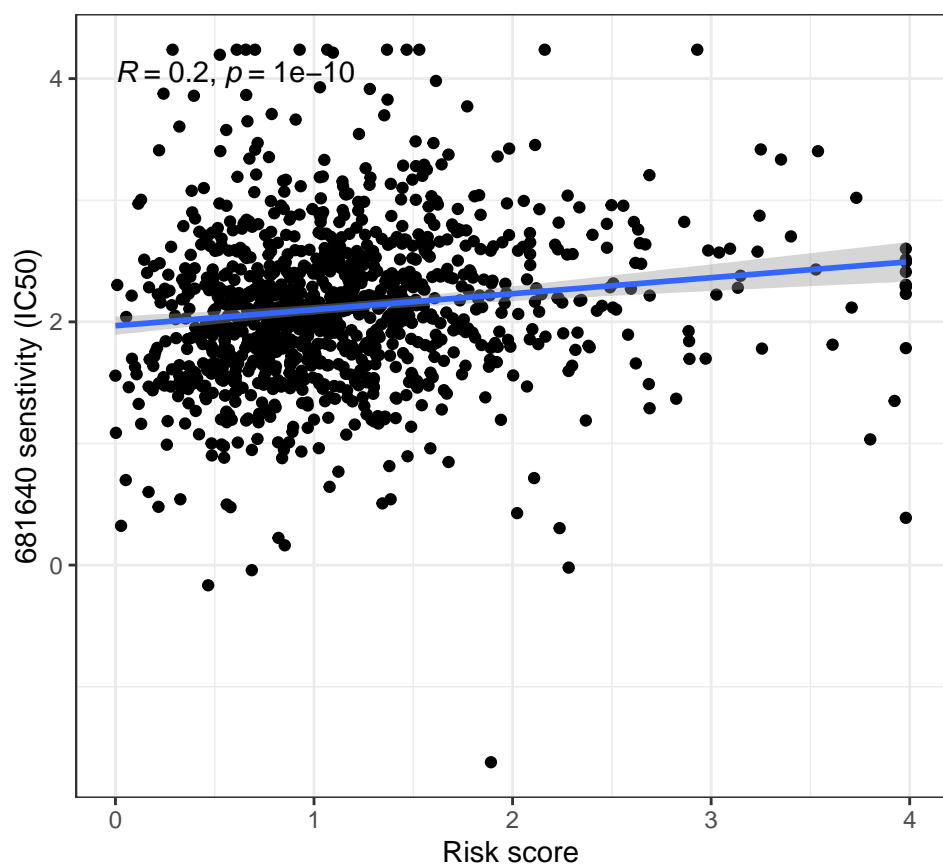

Supplement: Supplementary file 1 [file diagnostics-13-01203-s001.zip › Figure S3/Cor.681640.pdf]

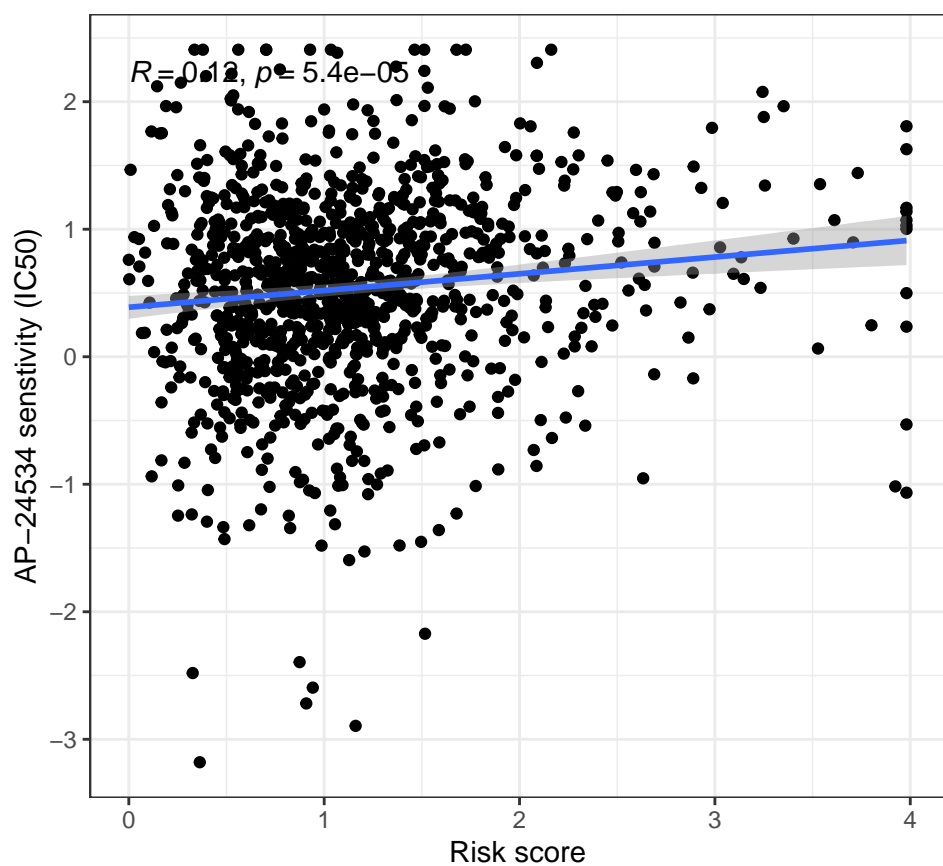

Supplement: Supplementary file 1 [file diagnostics-13-01203-s001.zip › Figure S3/Cor.AP-24534.pdf]

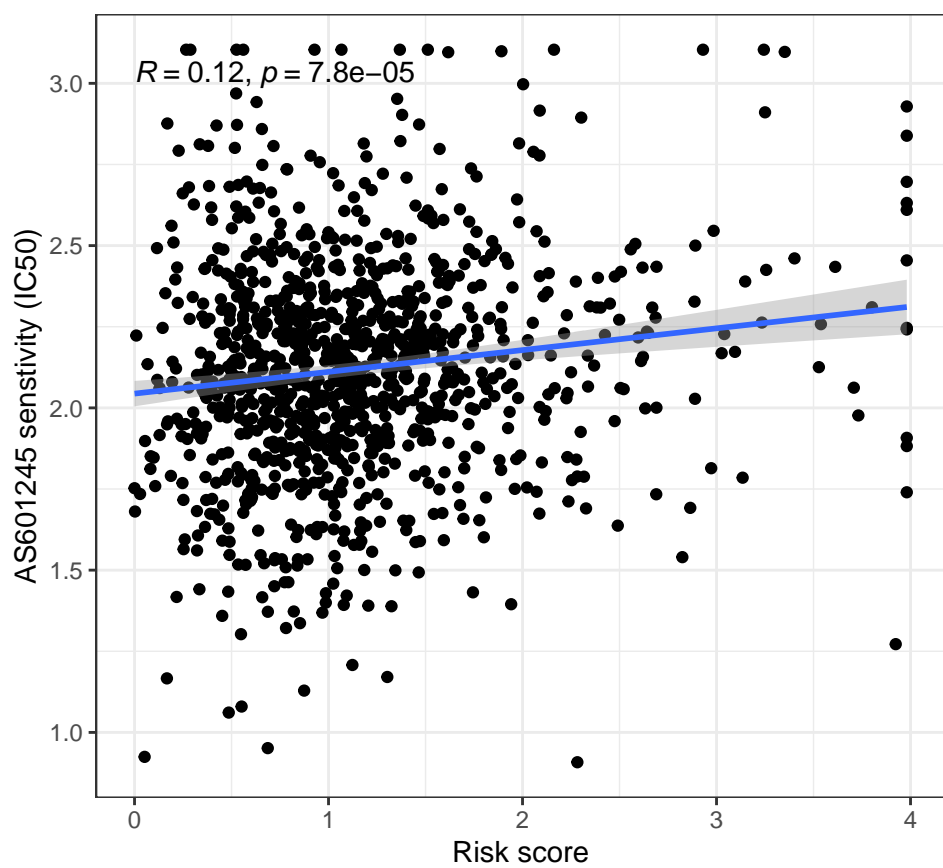

Supplement: Supplementary file 1 [file diagnostics-13-01203-s001.zip › Figure S3/Cor.AS601245.pdf]

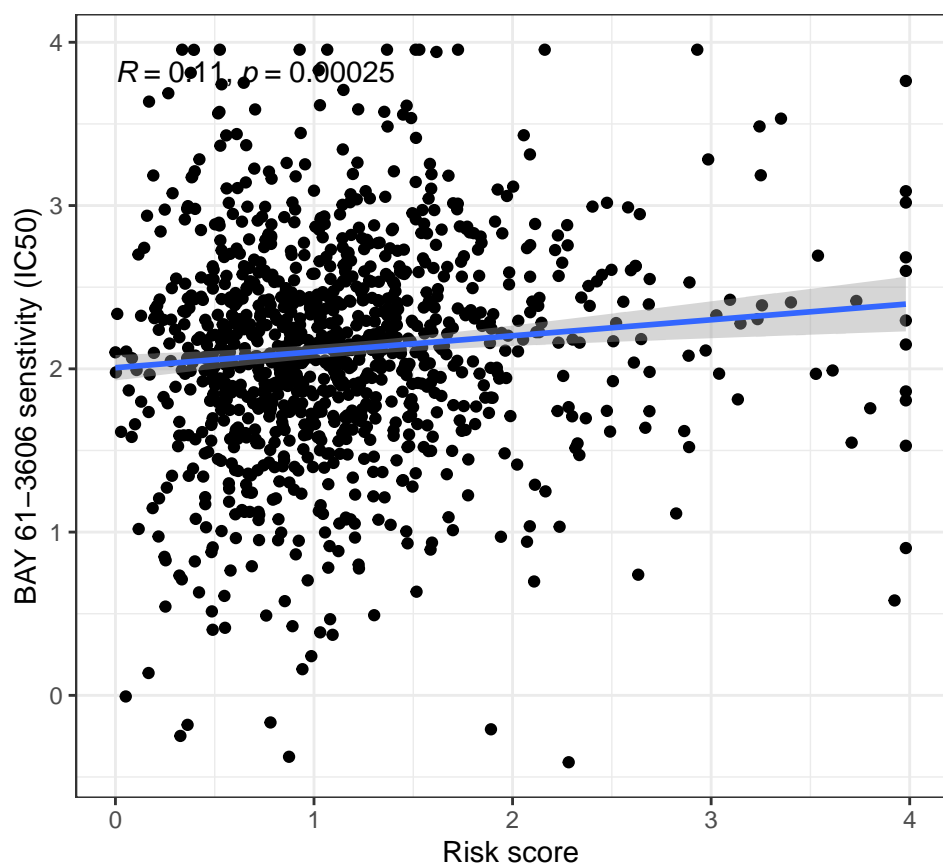

Supplement: Supplementary file 1 [file diagnostics-13-01203-s001.zip › Figure S3/Cor.BAY 61-3606.pdf]

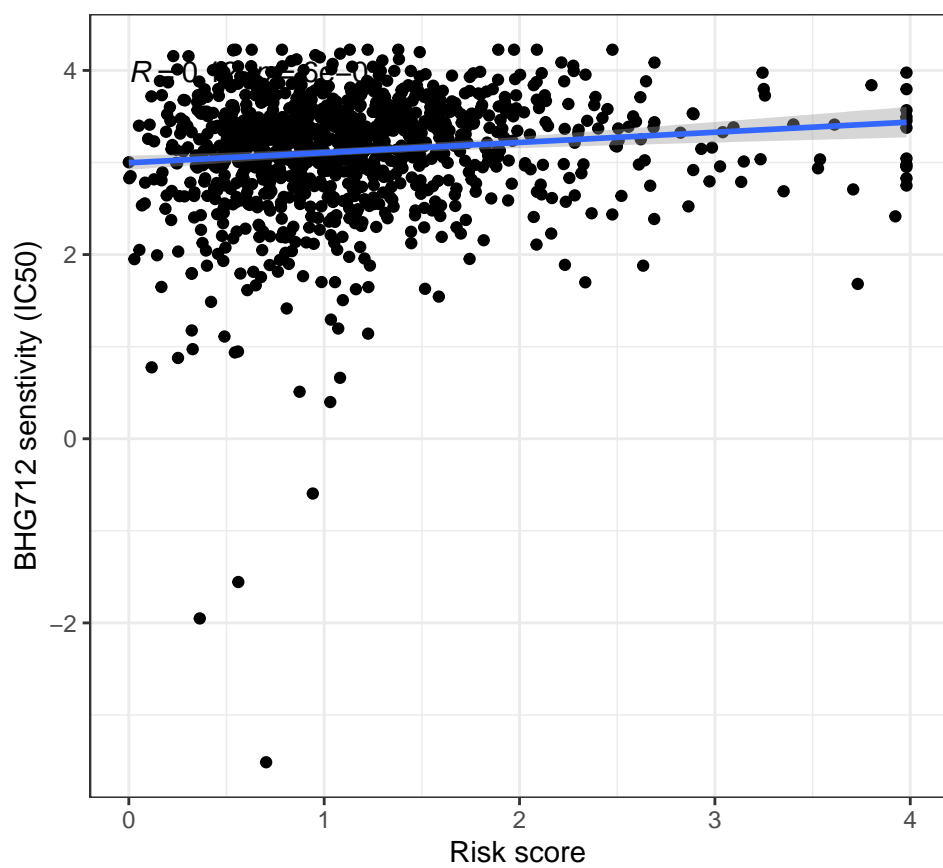

Supplement: Supplementary file 1 [file diagnostics-13-01203-s001.zip › Figure S3/Cor.BHG712.pdf]

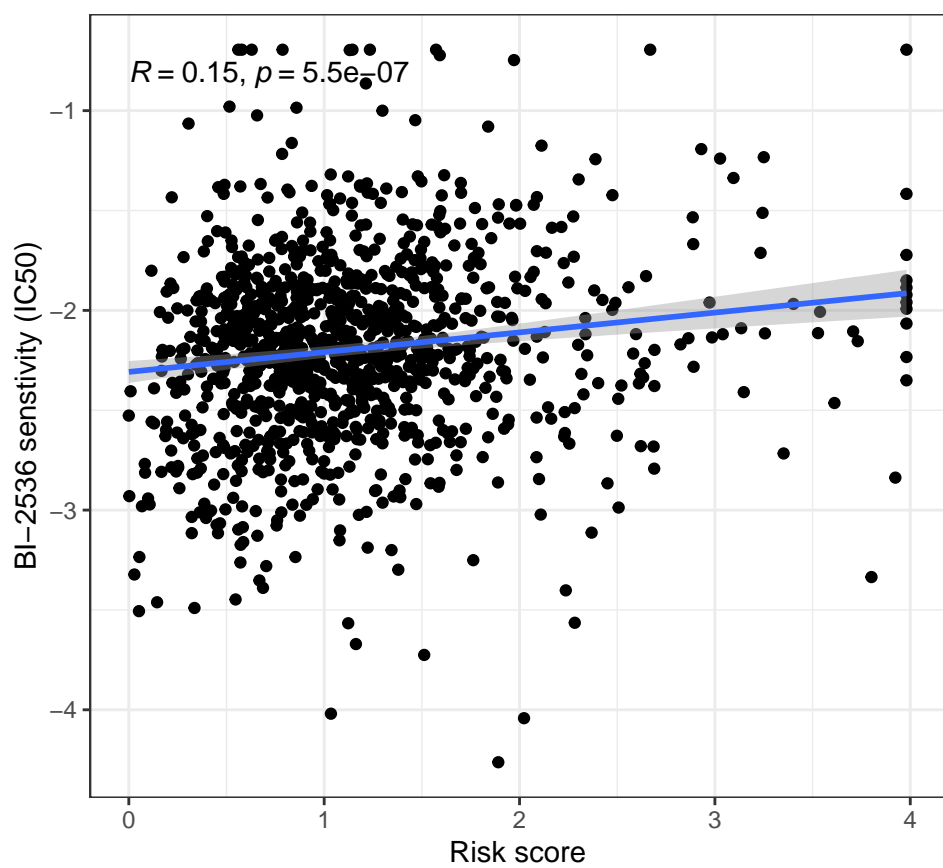

Supplement: Supplementary file 1 [file diagnostics-13-01203-s001.zip › Figure S3/Cor.BI-2536.pdf]

Bleomycin (50  $\mu$ M) sensitivity (IC50)

$R = 0.16$ ,  $p = 8.8e-08$

Risk score

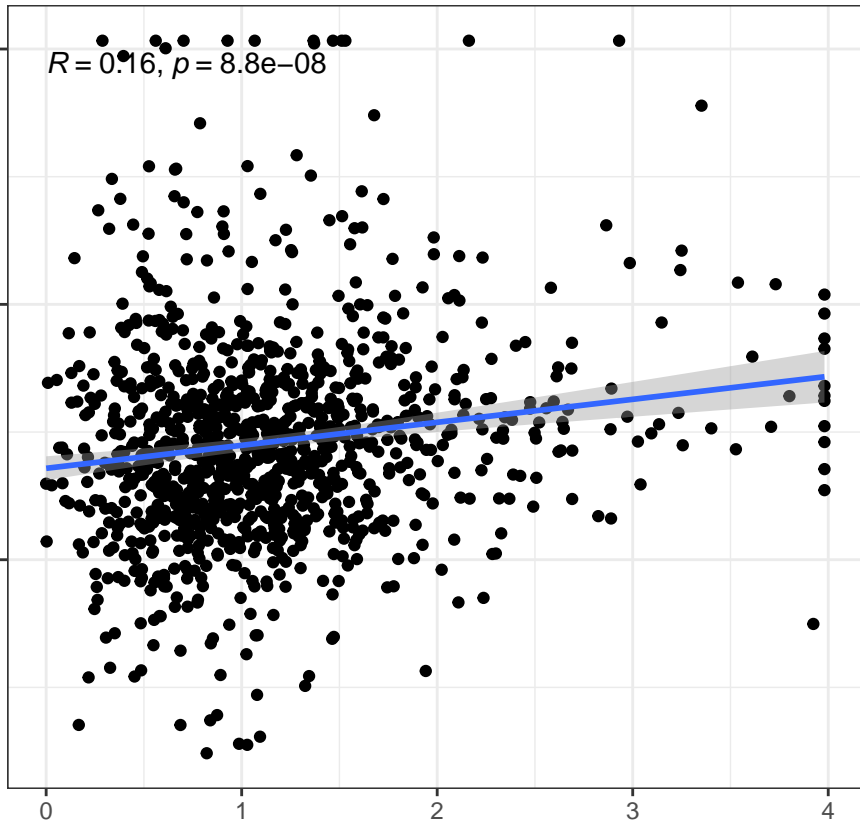

Supplement: Supplementary file 1 [file diagnostics-13-01203-s001.zip › Figure S3/Cor.Bleomycin (50 uM).pdf]

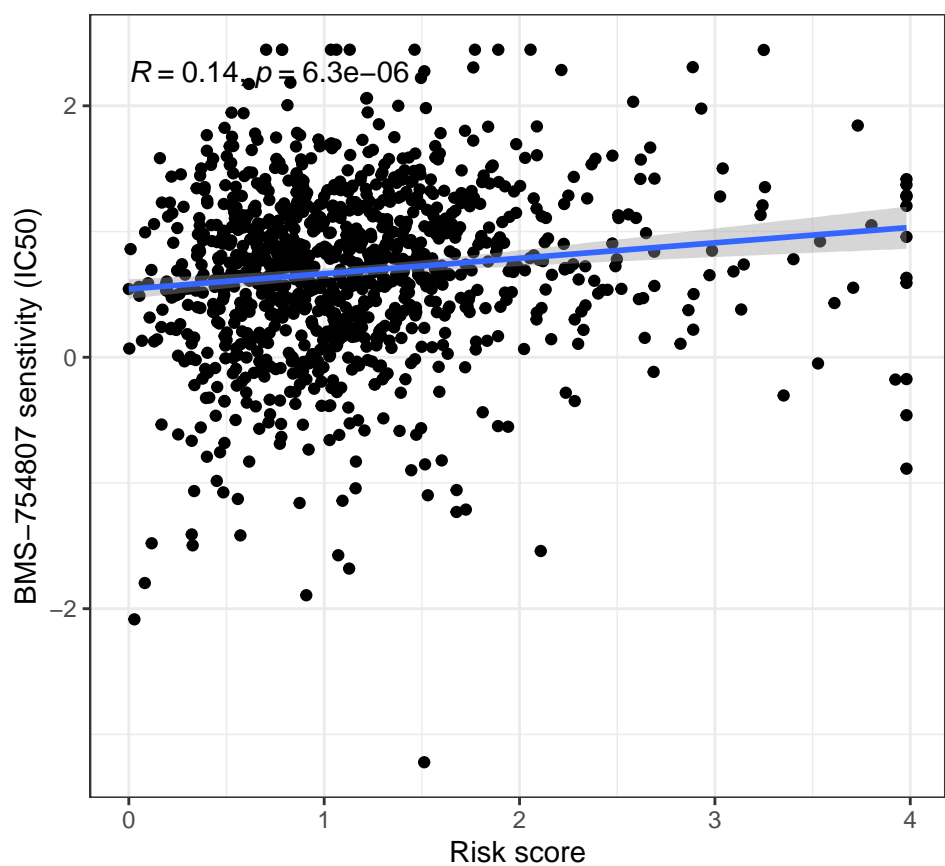

Supplement: Supplementary file 1 [file diagnostics-13-01203-s001.zip › Figure S3/Cor.BMS-754807.pdf]

BX-795 sensitivity (IC50)

$R = 0.18$ ,  $p = 3.1e-09$

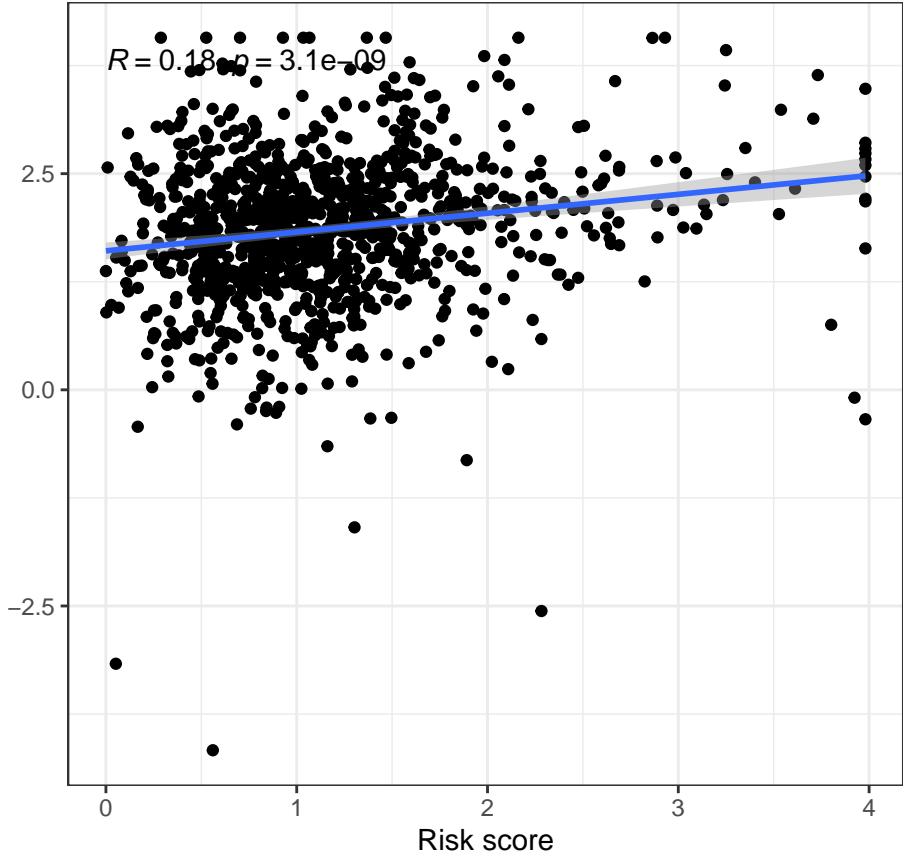

Supplement: Supplementary file 1 [file diagnostics-13-01203-s001.zip › Figure S3/Cor.BX-795.pdf]

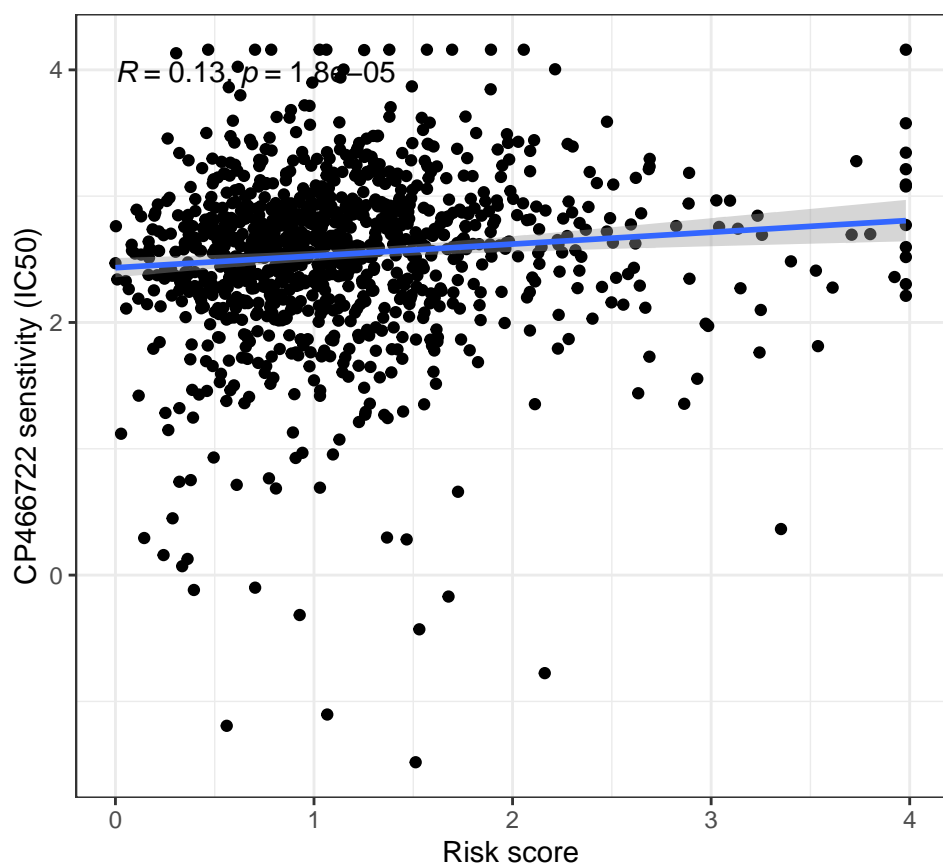

Supplement: Supplementary file 1 [file diagnostics-13-01203-s001.zip › Figure S3/Cor.CP466722.pdf]

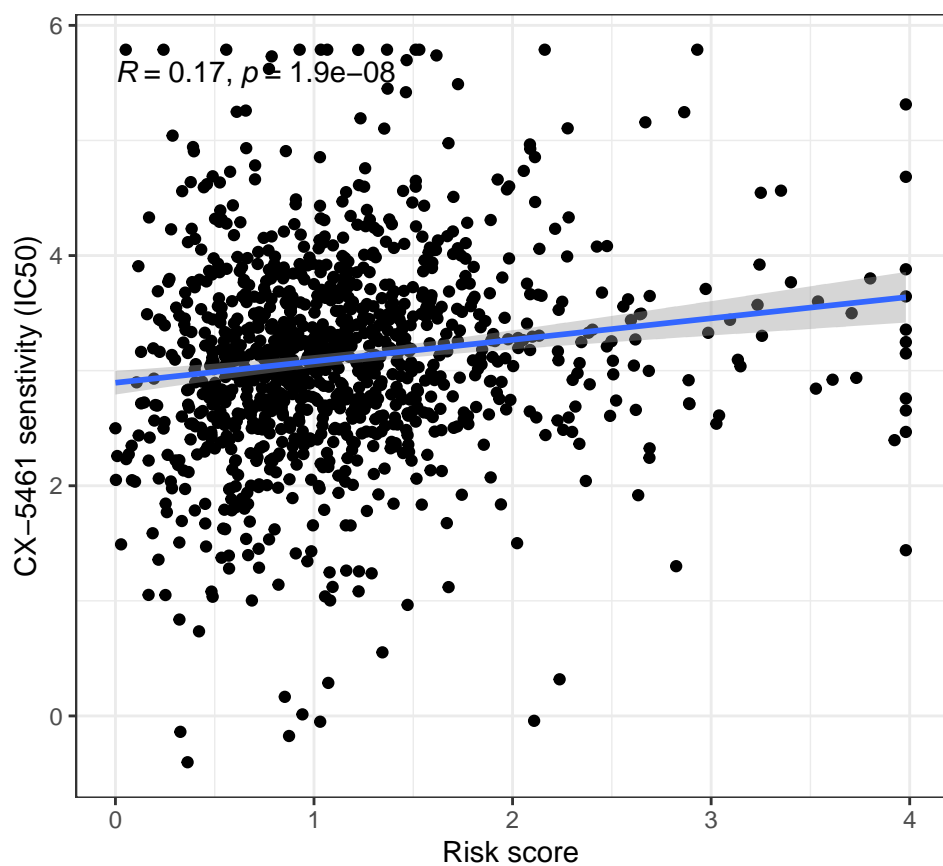

Supplement: Supplementary file 1 [file diagnostics-13-01203-s001.zip › Figure S3/Cor.CX-5461.pdf]

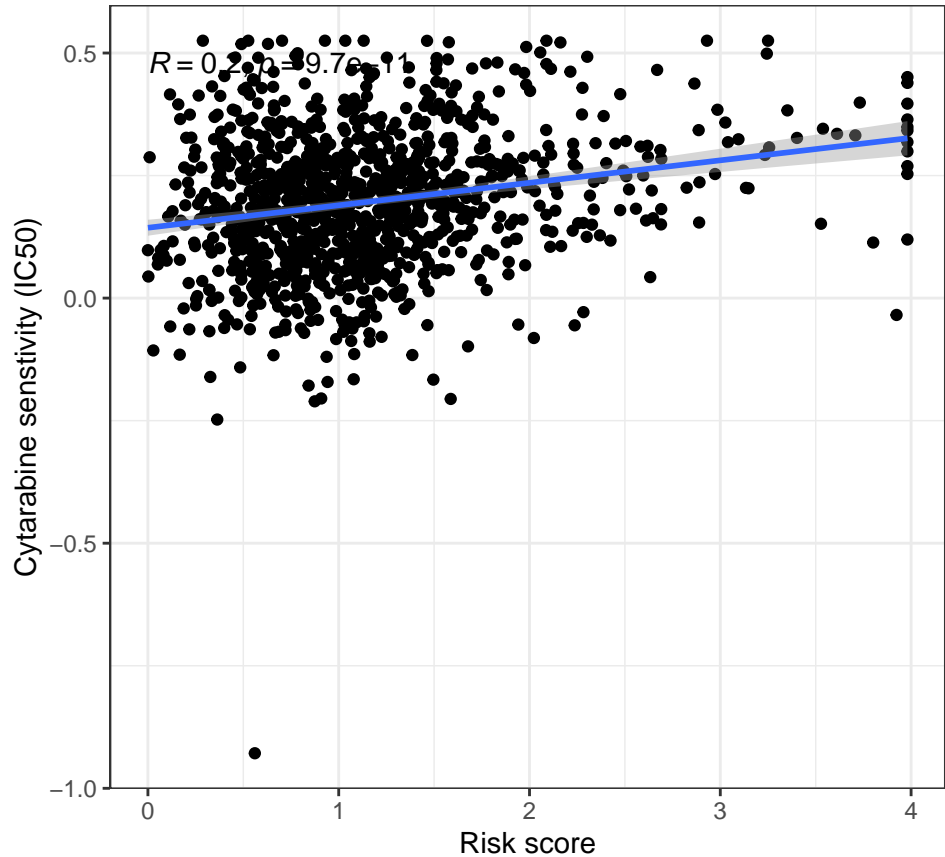

Supplement: Supplementary file 1 [file diagnostics-13-01203-s001.zip › Figure S3/Cor.Cytarabine.pdf]

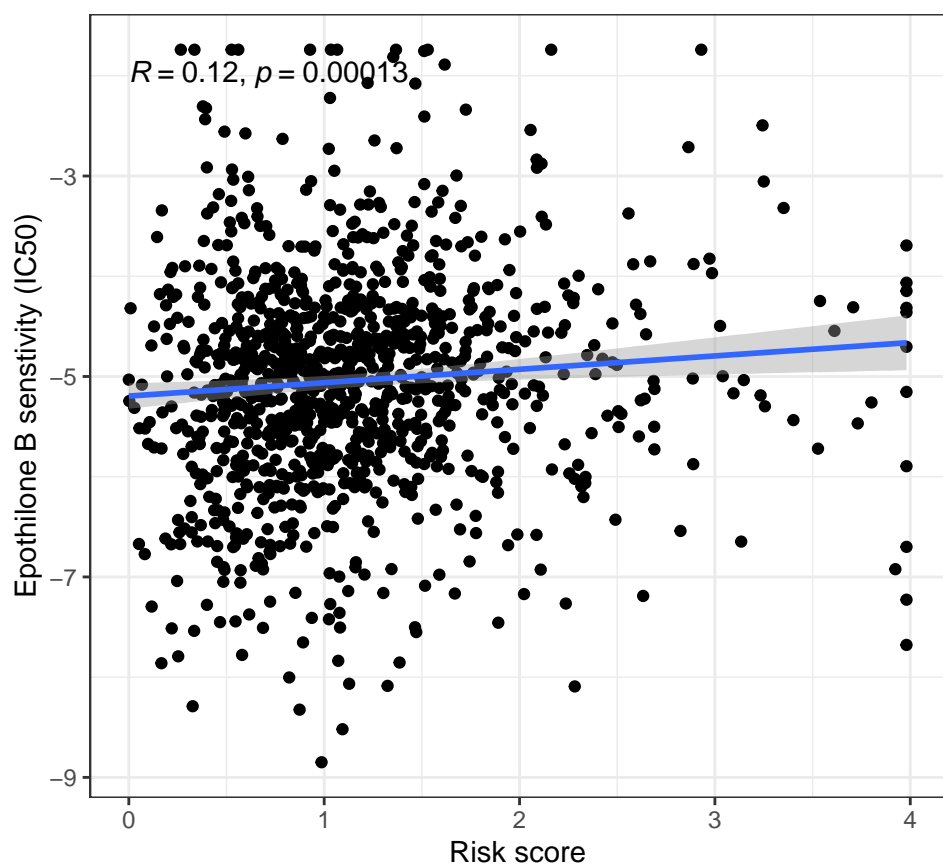

Supplement: Supplementary file 1 [file diagnostics-13-01203-s001.zip › Figure S3/Cor.Epothilone B.pdf]

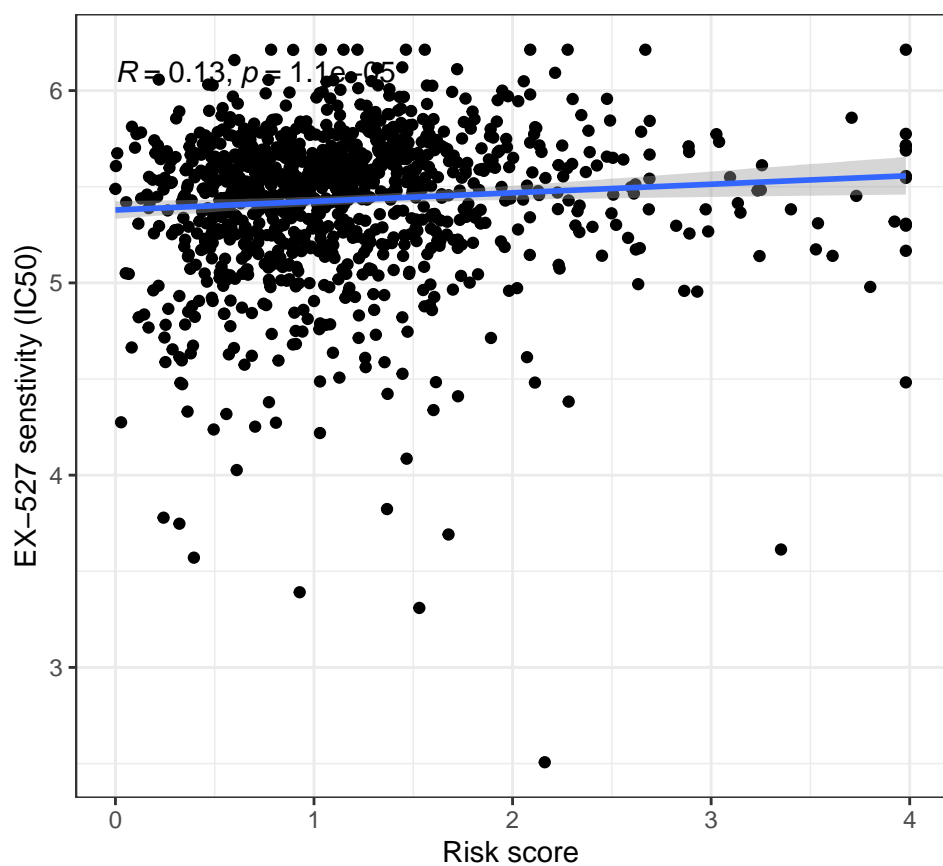

Supplement: Supplementary file 1 [file diagnostics-13-01203-s001.zip › Figure S3/Cor.EX-527.pdf]

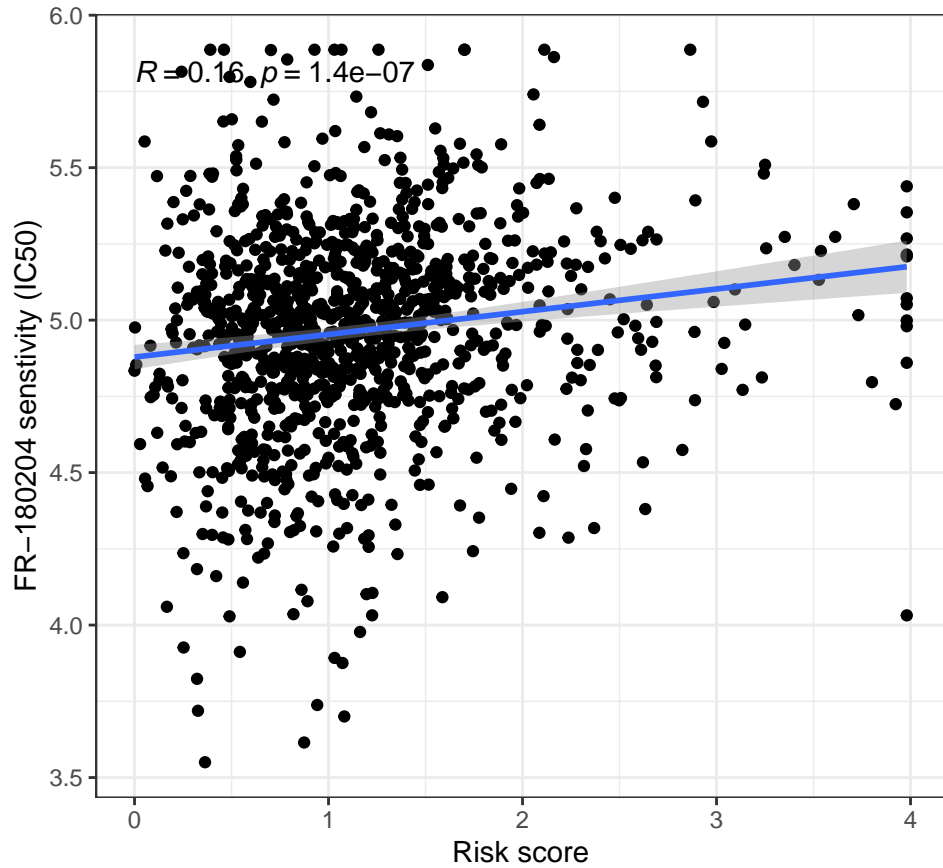

Supplement: Supplementary file 1 [file diagnostics-13-01203-s001.zip › Figure S3/Cor.FR-180204.pdf]

GSK1070916 sensitivity (IC50)

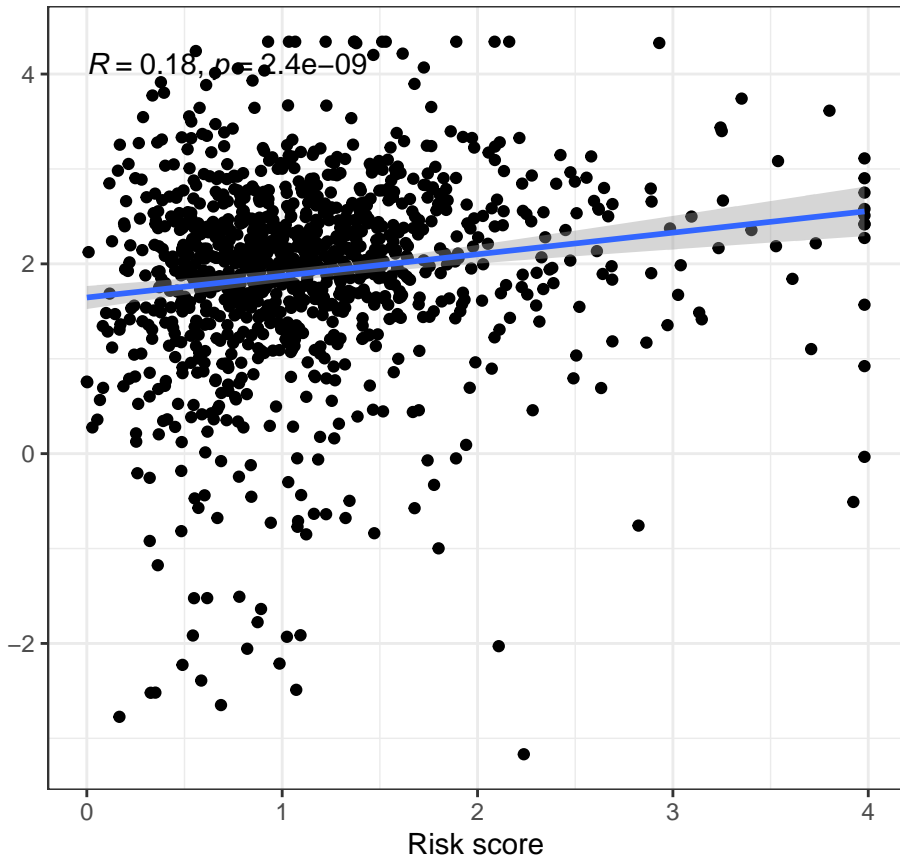

Supplement: Supplementary file 1 [file diagnostics-13-01203-s001.zip › Figure S3/Cor.GSK1070916.pdf]

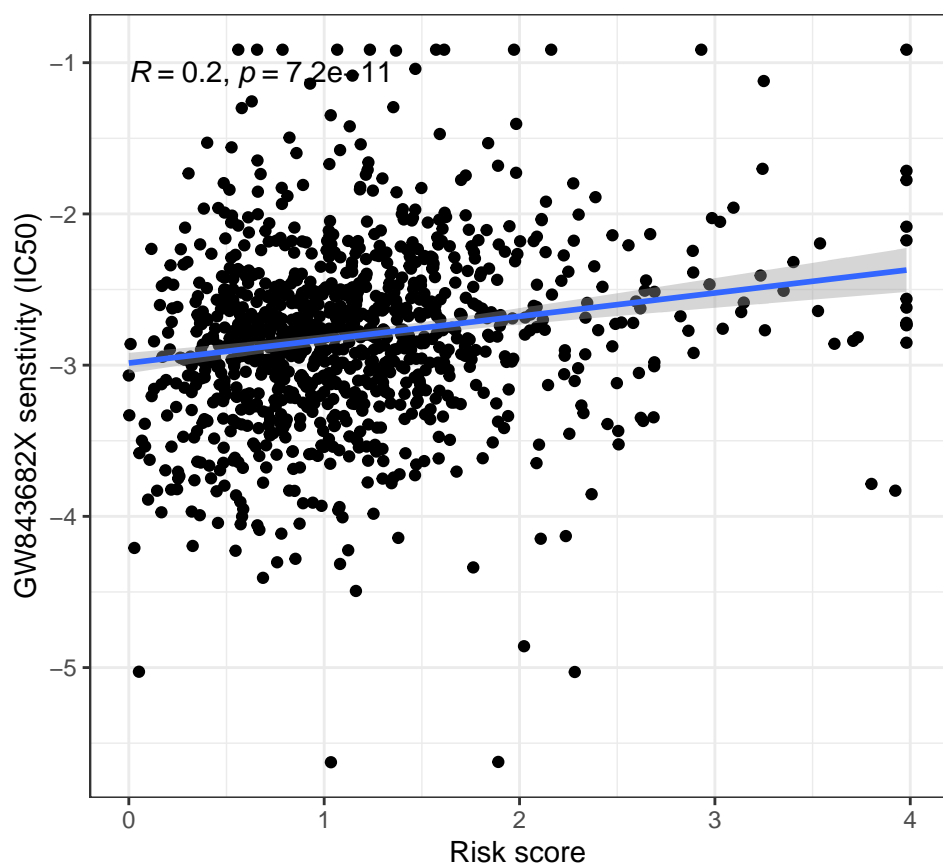

Supplement: Supplementary file 1 [file diagnostics-13-01203-s001.zip › Figure S3/Cor.GW843682X.pdf]

HG-5-113-01 sensitivity (IC50)

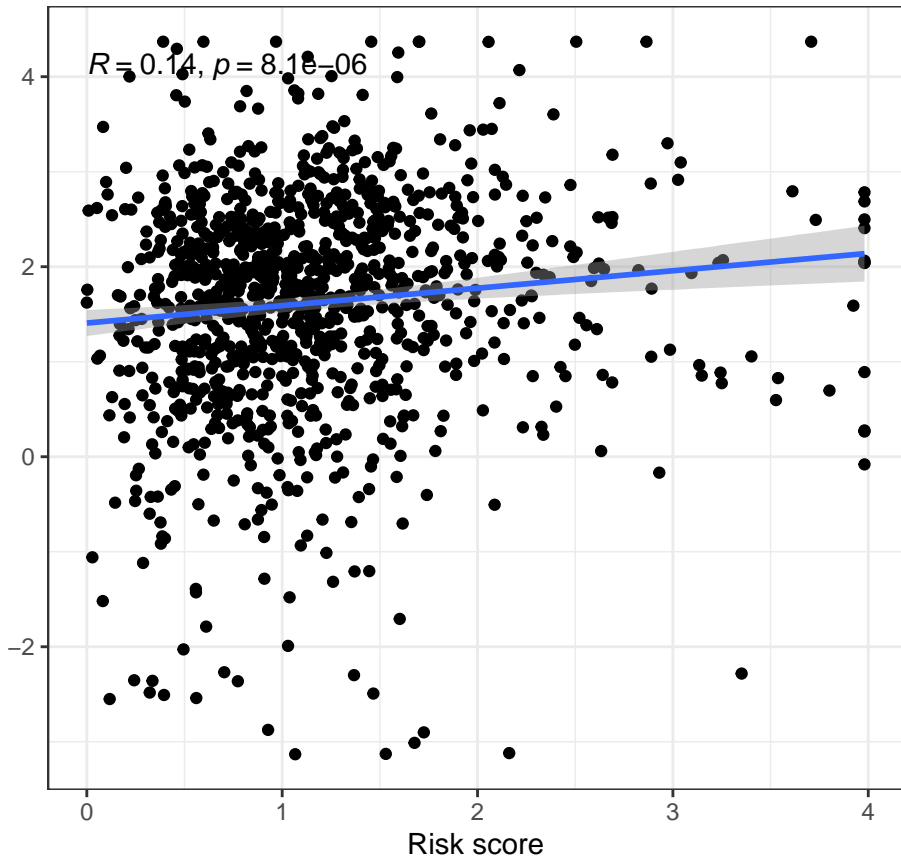

Supplement: Supplementary file 1 [file diagnostics-13-01203-s001.zip › Figure S3/Cor.HG-5-113-01.pdf]

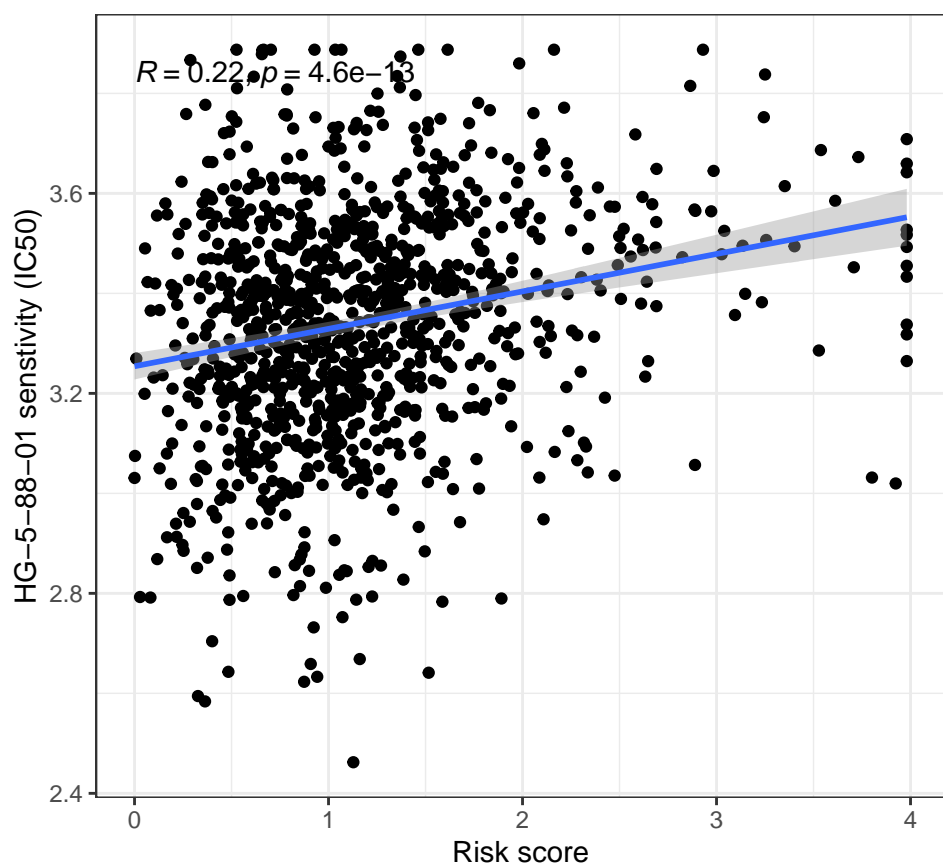

Supplement: Supplementary file 1 [file diagnostics-13-01203-s001.zip › Figure S3/Cor.HG-5-88-01.pdf]

Ispinesib Mesylate sensitivity (IC50)

$R = 0.16$ ,  $p = 1.5e-07$

0

1

2

3

4

Risk score

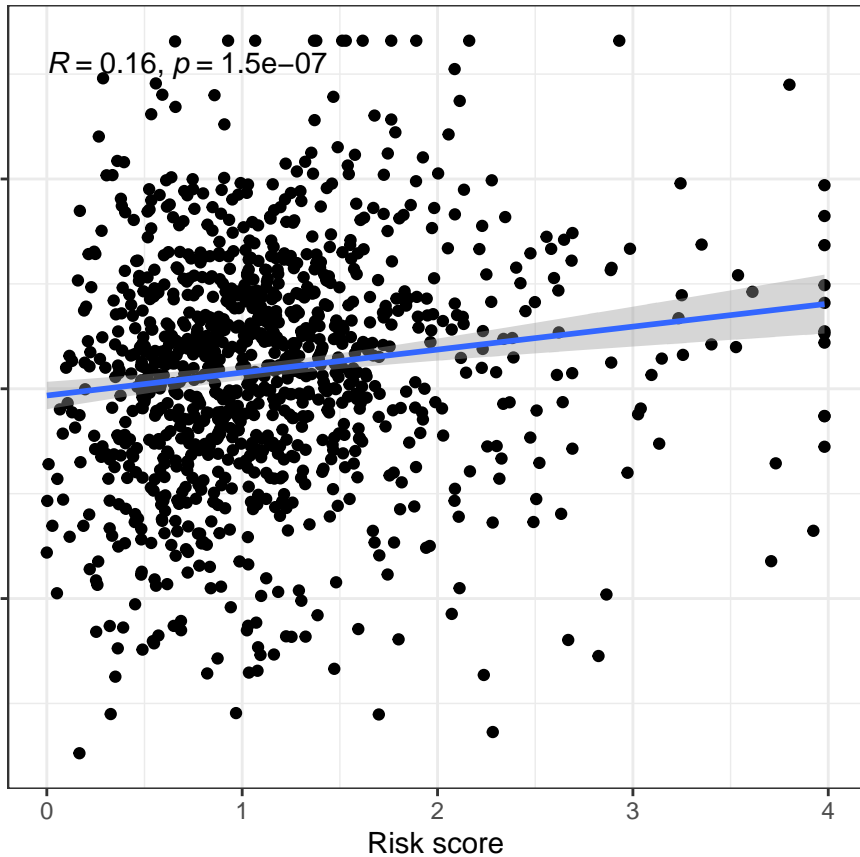

Supplement: Supplementary file 1 [file diagnostics-13-01203-s001.zip › Figure S3/Cor.Ispinesib Mesylate.pdf]

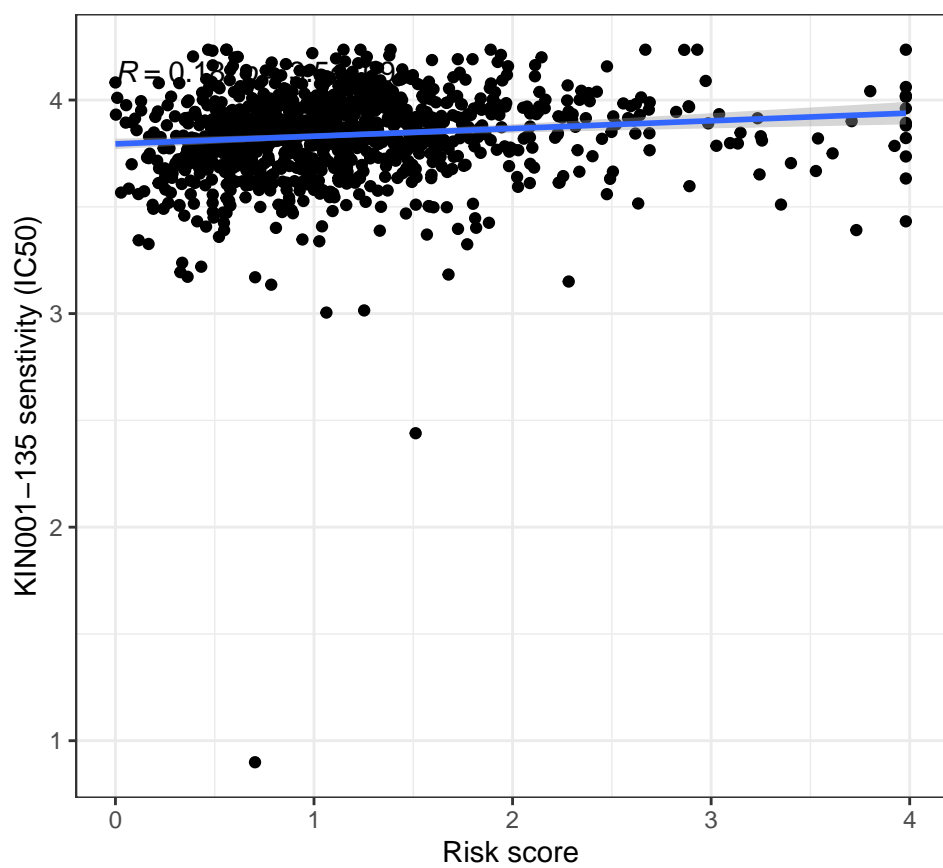

Supplement: Supplementary file 1 [file diagnostics-13-01203-s001.zip › Figure S3/Cor.KIN001-135.pdf]

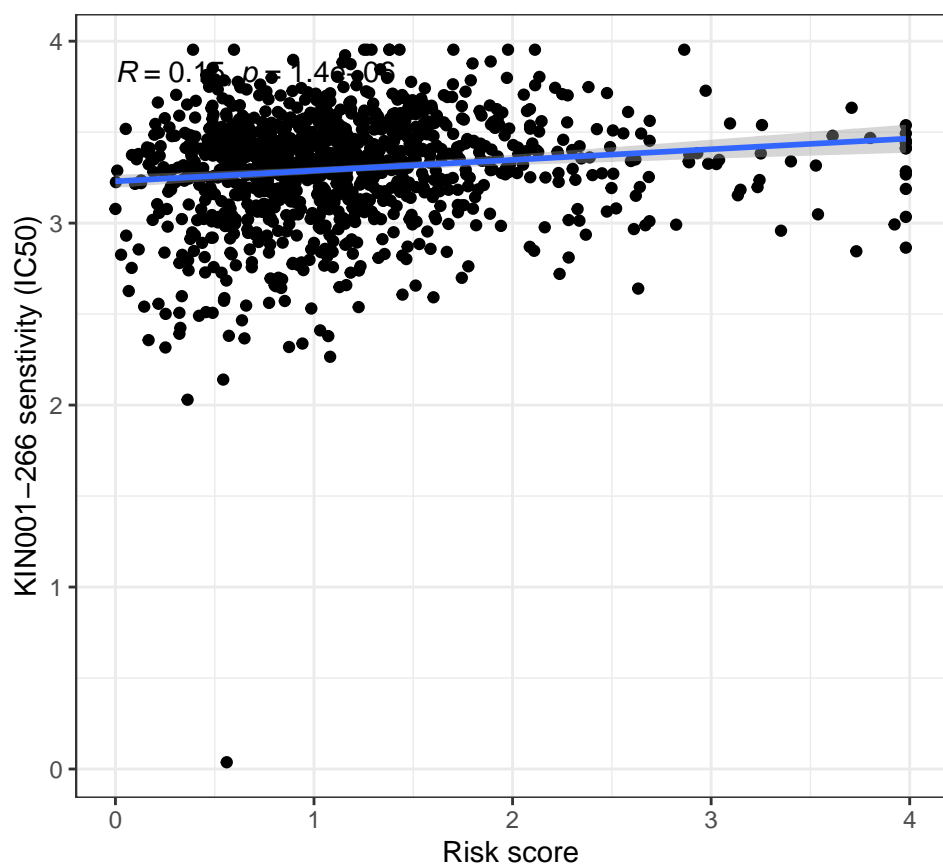

Supplement: Supplementary file 1 [file diagnostics-13-01203-s001.zip › Figure S3/Cor.KIN001-266.pdf]

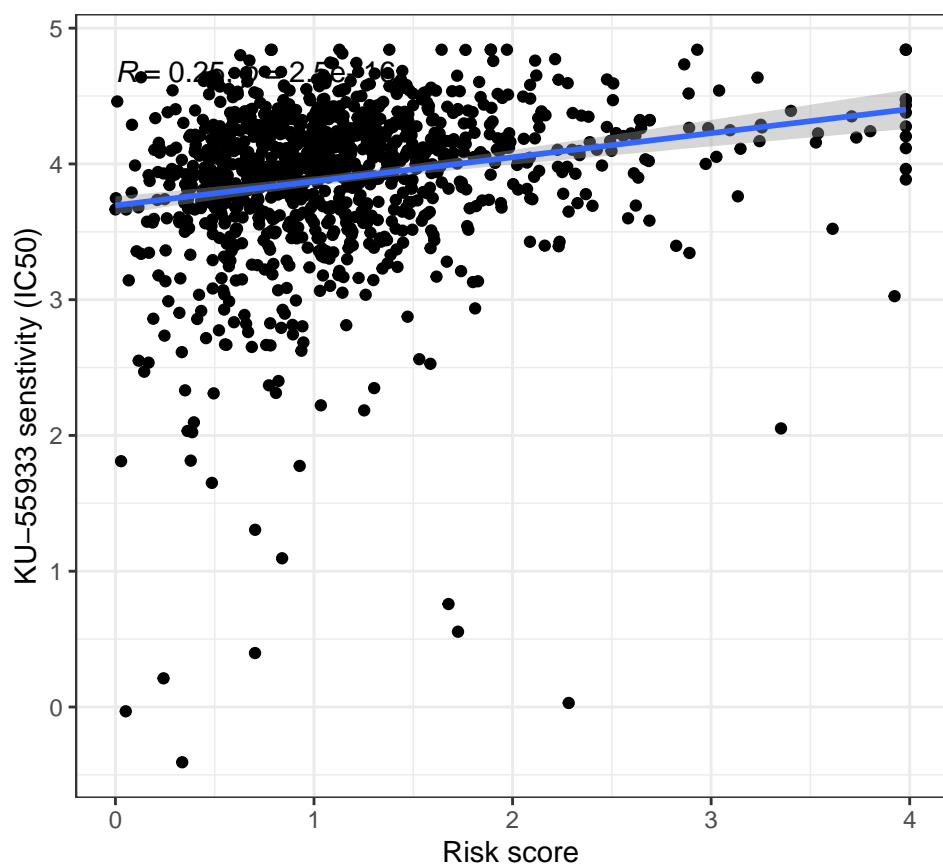

Supplement: Supplementary file 1 [file diagnostics-13-01203-s001.zip › Figure S3/Cor.KU-55933.pdf]

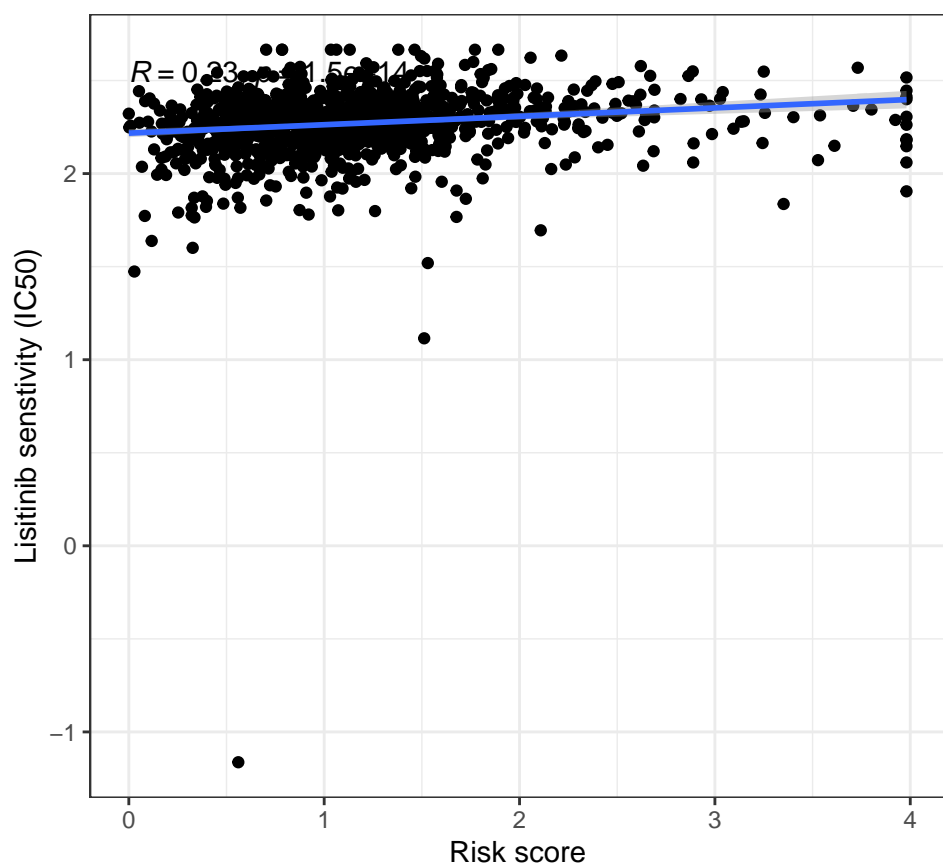

Supplement: Supplementary file 1 [file diagnostics-13-01203-s001.zip › Figure S3/Cor.Lisitinib.pdf]

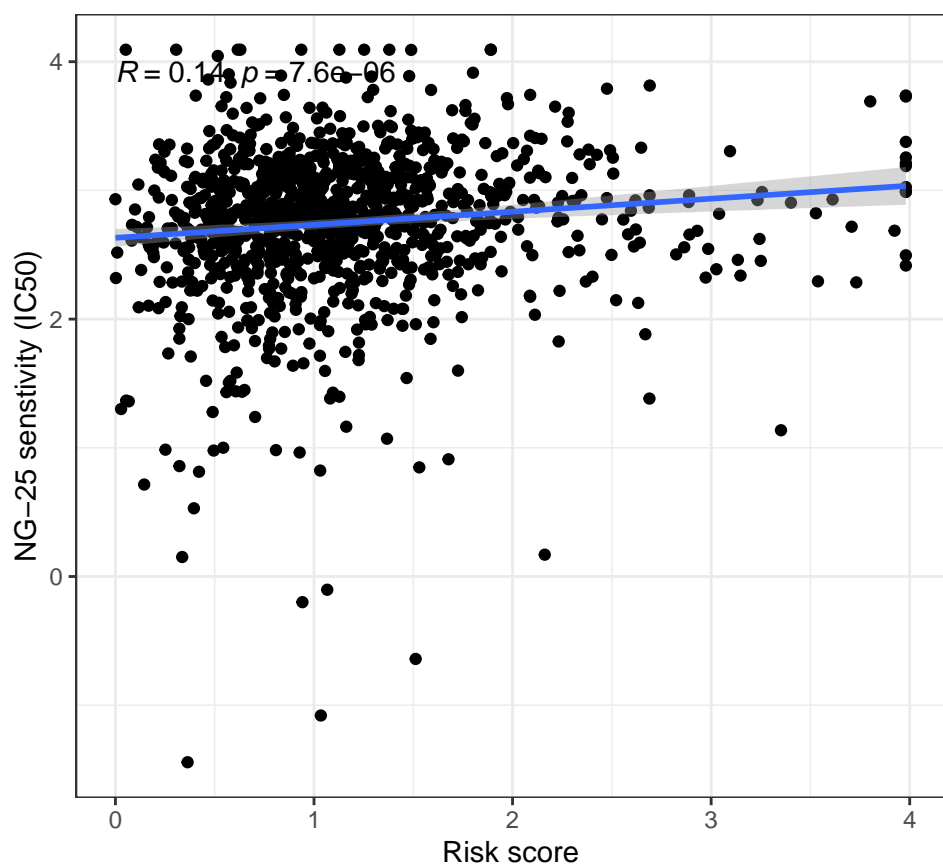

Supplement: Supplementary file 1 [file diagnostics-13-01203-s001.zip › Figure S3/Cor.NG-25.pdf]

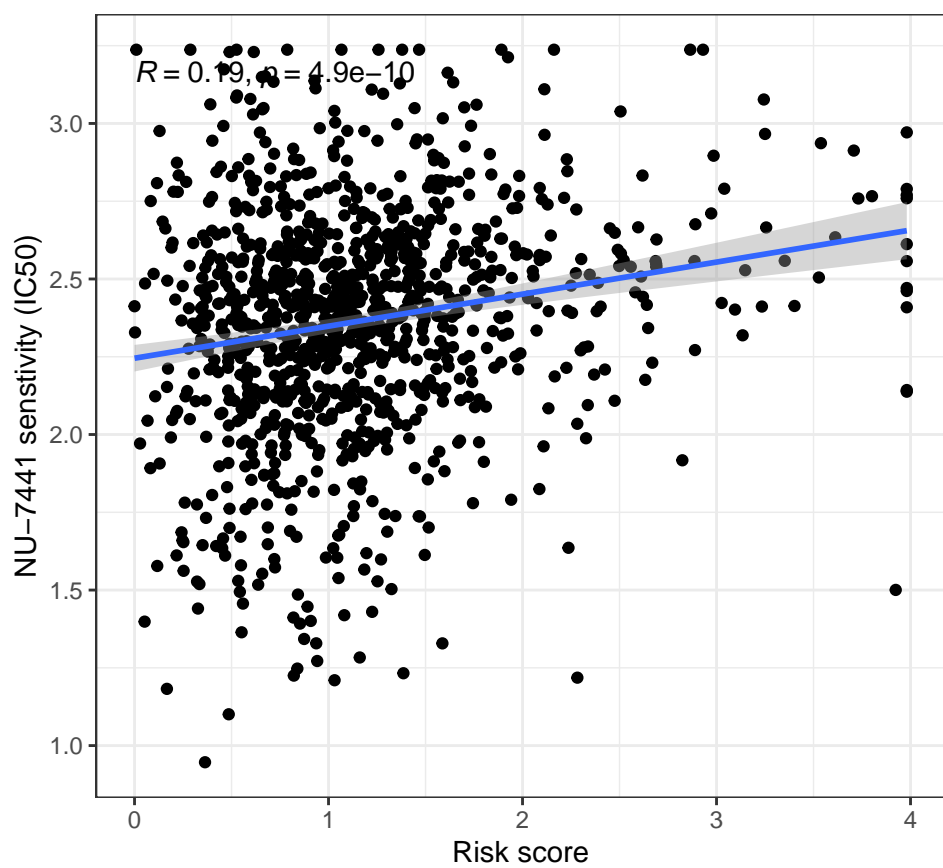

Supplement: Supplementary file 1 [file diagnostics-13-01203-s001.zip › Figure S3/Cor.NU-7441.pdf]

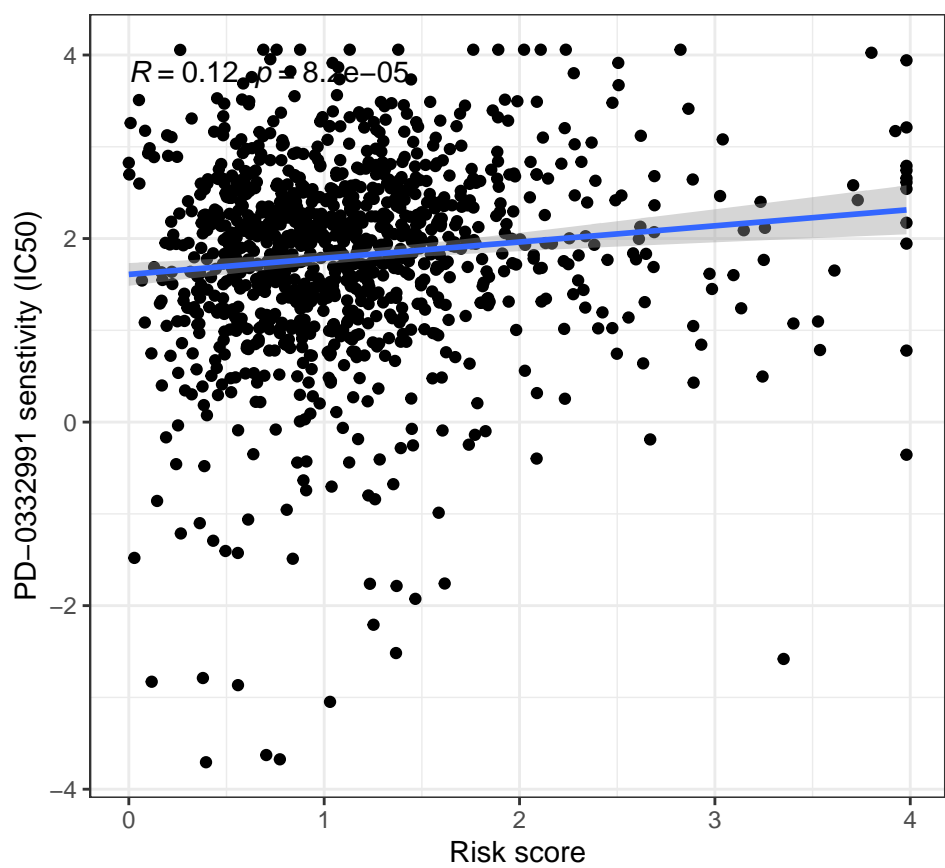

Supplement: Supplementary file 1 [file diagnostics-13-01203-s001.zip › Figure S3/Cor.PD-0332991.pdf]

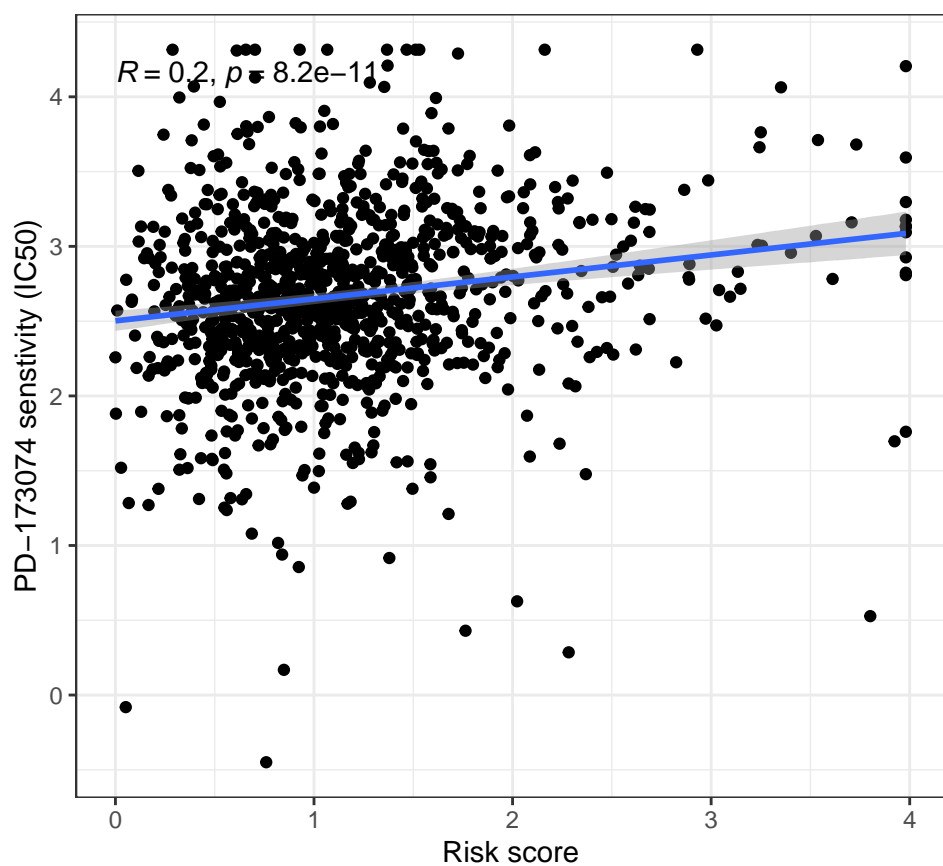

Supplement: Supplementary file 1 [file diagnostics-13-01203-s001.zip › Figure S3/Cor.PD-173074.pdf]

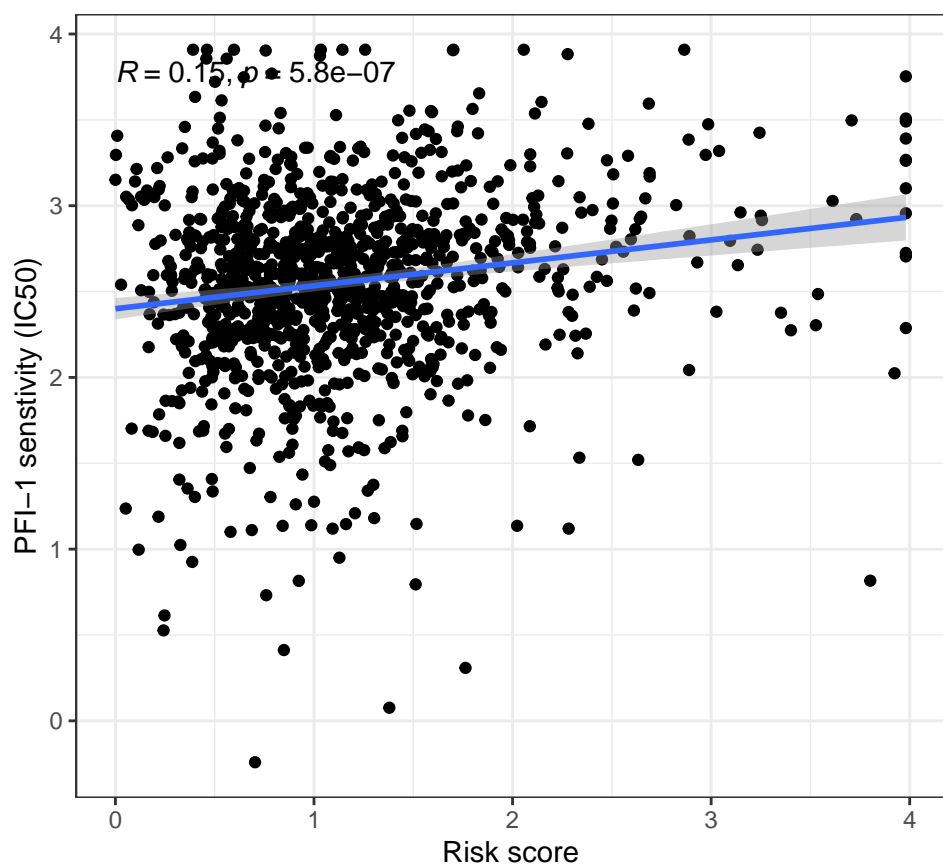

Supplement: Supplementary file 1 [file diagnostics-13-01203-s001.zip › Figure S3/Cor.PFI-1.pdf]

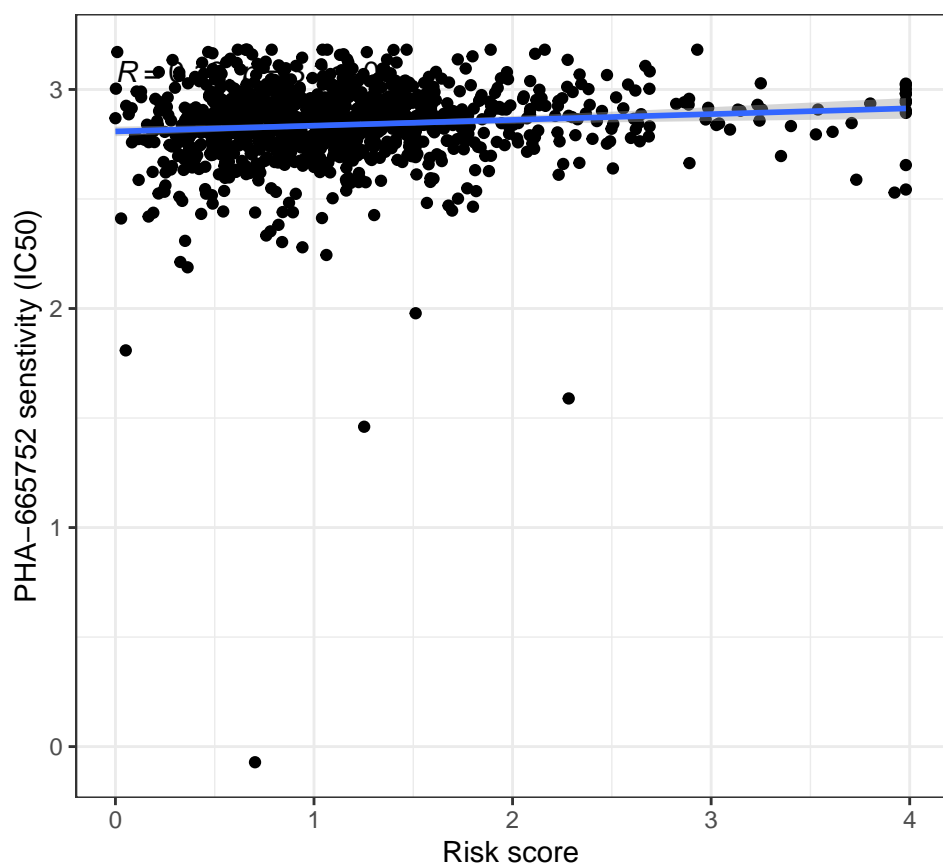

Supplement: Supplementary file 1 [file diagnostics-13-01203-s001.zip › Figure S3/Cor.PHA-665752.pdf]

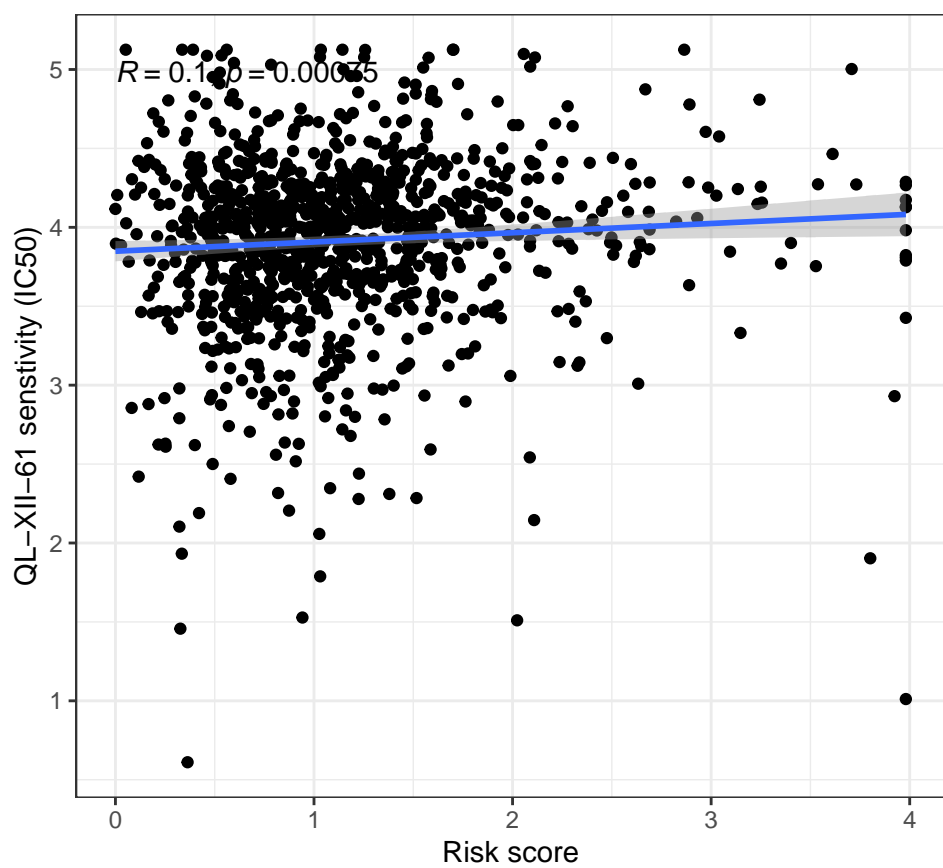

Supplement: Supplementary file 1 [file diagnostics-13-01203-s001.zip › Figure S3/Cor.QL-XII-61.pdf]

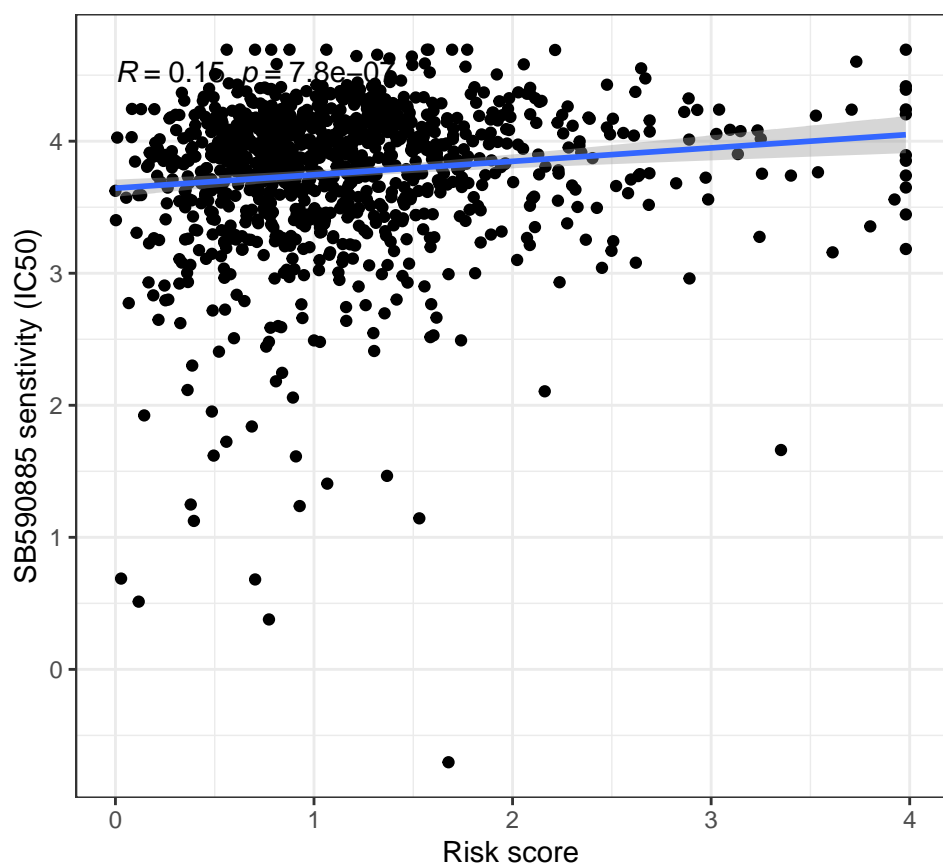

Supplement: Supplementary file 1 [file diagnostics-13-01203-s001.zip › Figure S3/Cor.SB590885.pdf]

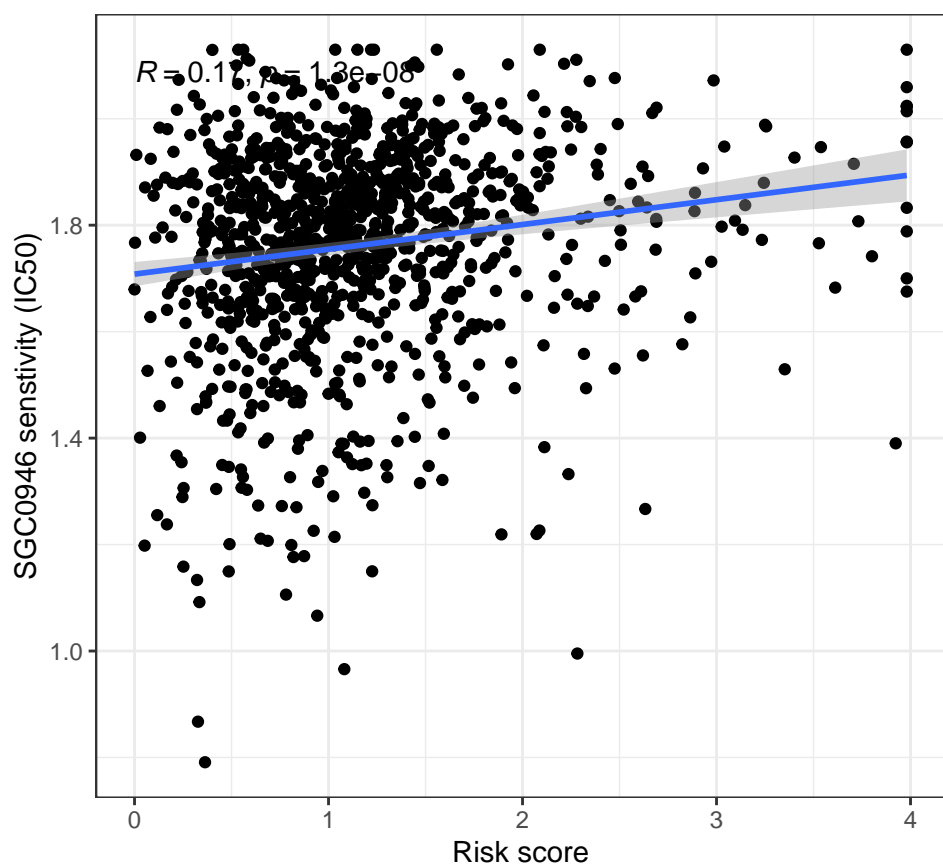

Supplement: Supplementary file 1 [file diagnostics-13-01203-s001.zip › Figure S3/Cor.SGC0946.pdf]

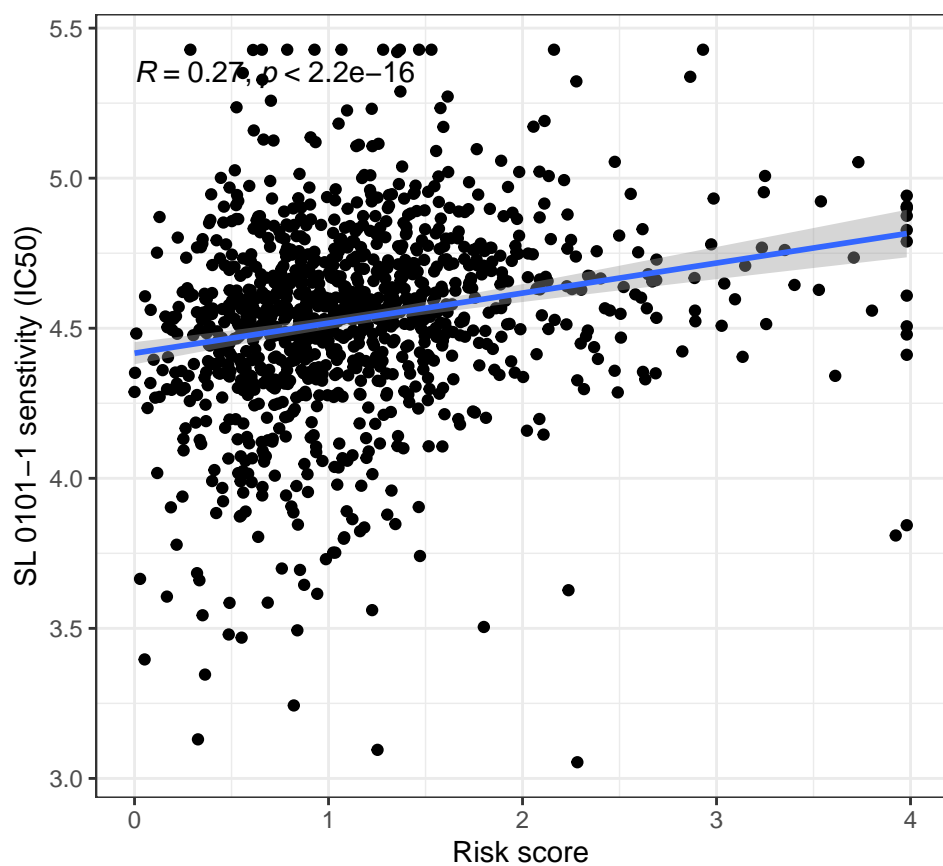

Supplement: Supplementary file 1 [file diagnostics-13-01203-s001.zip › Figure S3/Cor.SL 0101-1.pdf]

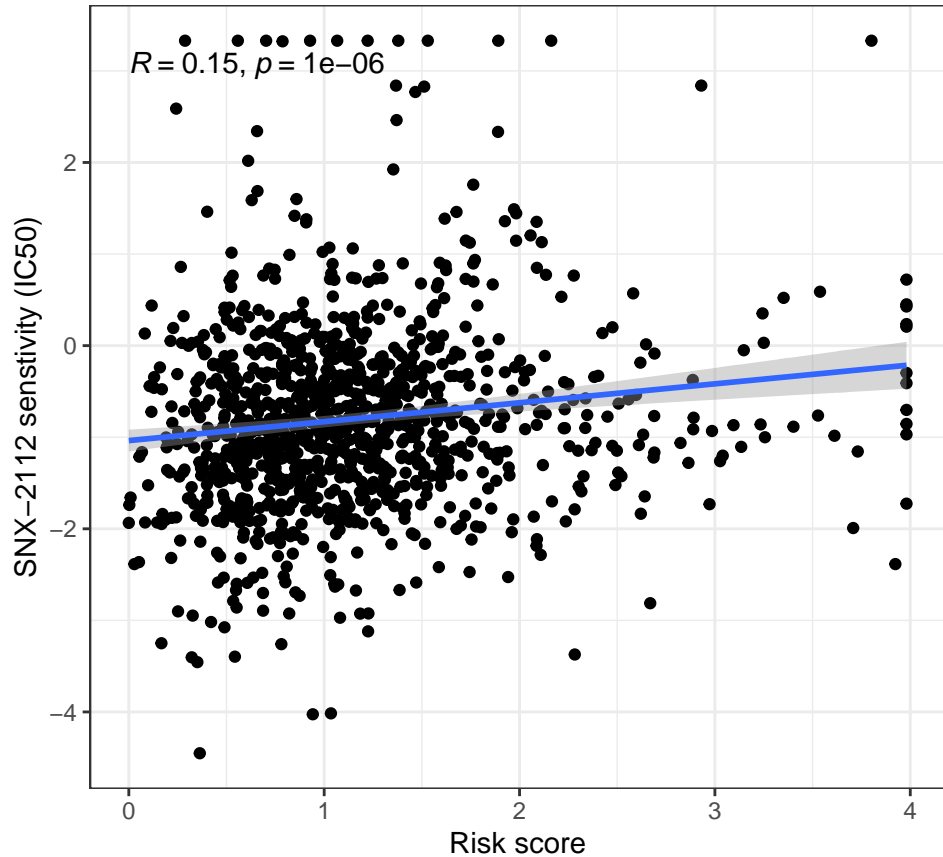

Supplement: Supplementary file 1 [file diagnostics-13-01203-s001.zip › Figure S3/Cor.SNX-2112.pdf]

Sunitinib sensitivity (IC50)

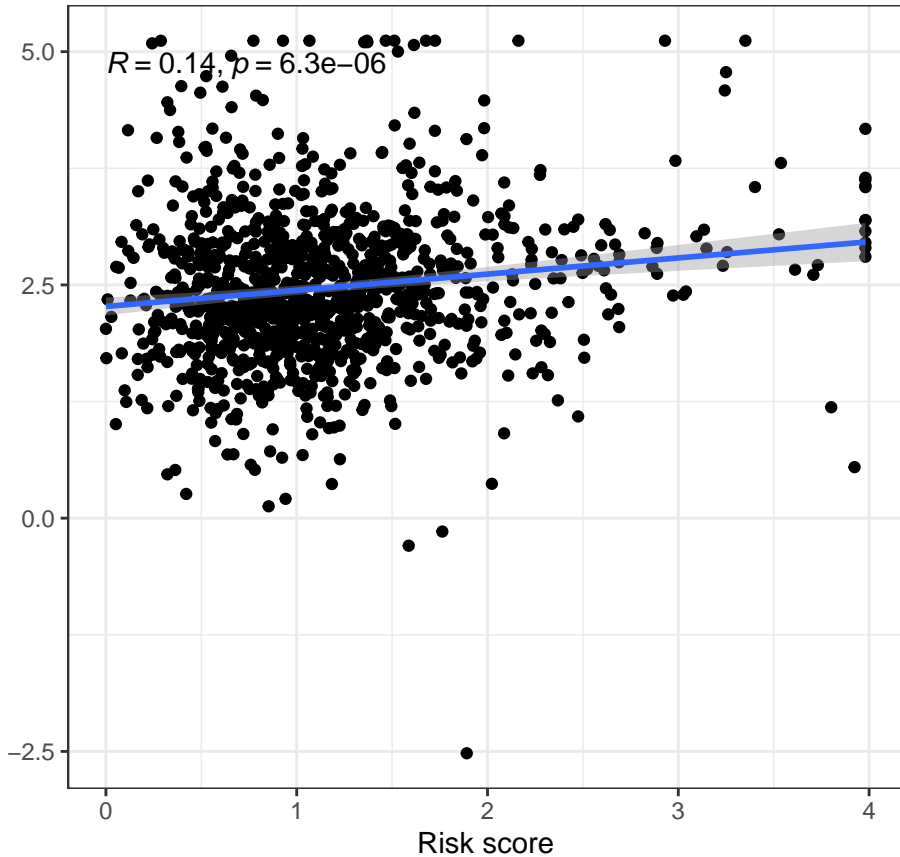

Supplement: Supplementary file 1 [file diagnostics-13-01203-s001.zip › Figure S3/Cor.Sunitinib.pdf]

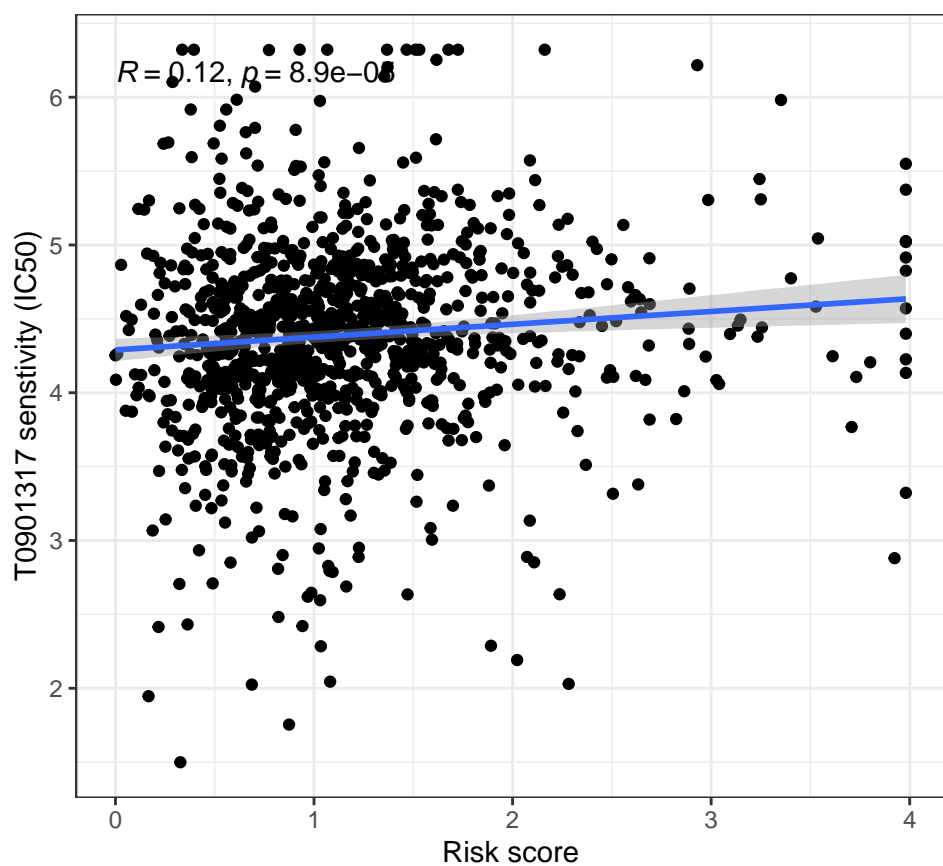

Supplement: Supplementary file 1 [file diagnostics-13-01203-s001.zip › Figure S3/Cor.T0901317.pdf]

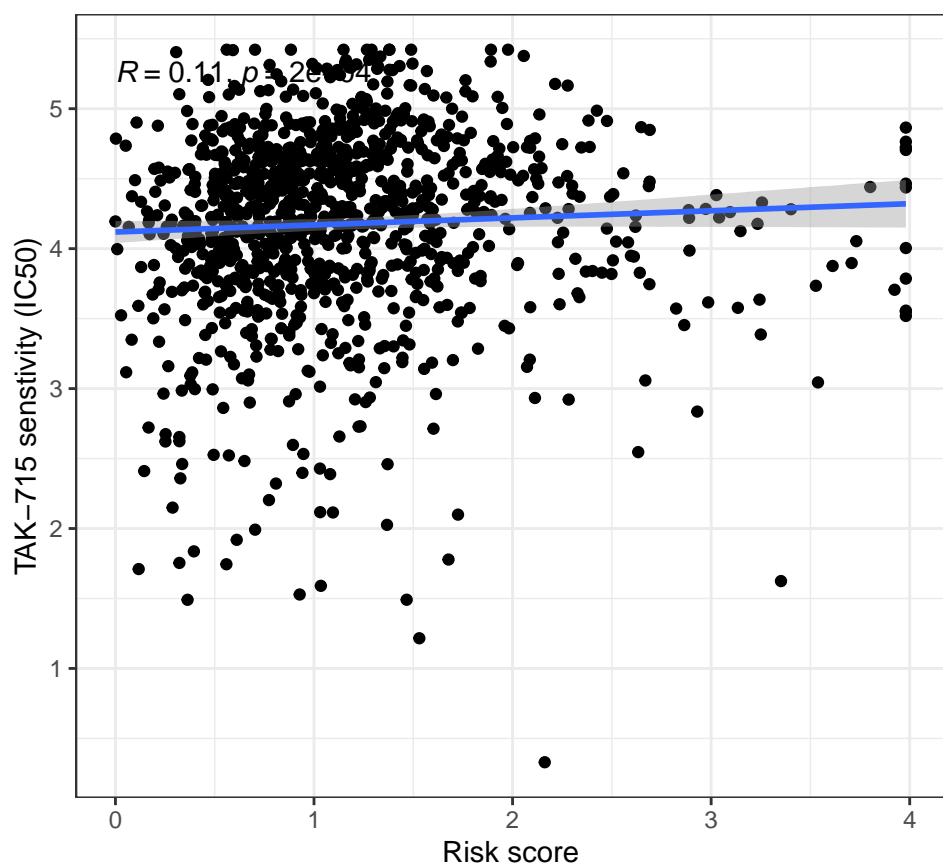

Supplement: Supplementary file 1 [file diagnostics-13-01203-s001.zip › Figure S3/Cor.TAK-715.pdf]

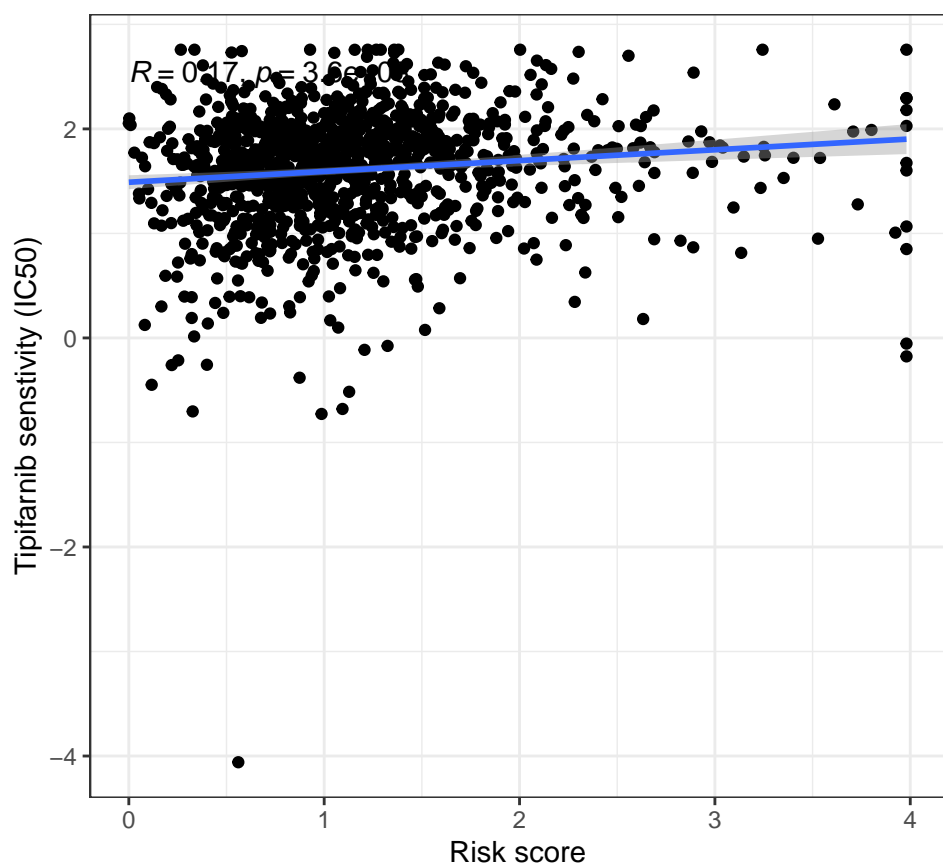

Supplement: Supplementary file 1 [file diagnostics-13-01203-s001.zip › Figure S3/Cor.Tipifarnib.pdf]

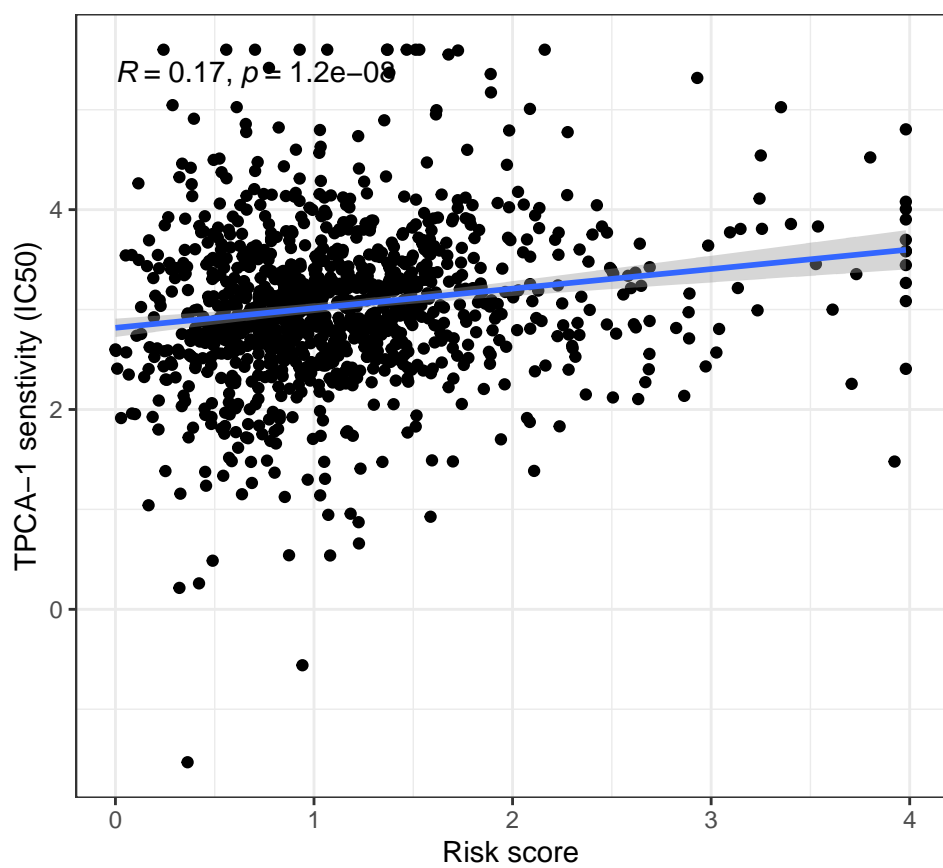

Supplement: Supplementary file 1 [file diagnostics-13-01203-s001.zip › Figure S3/Cor.TPCA-1.pdf]

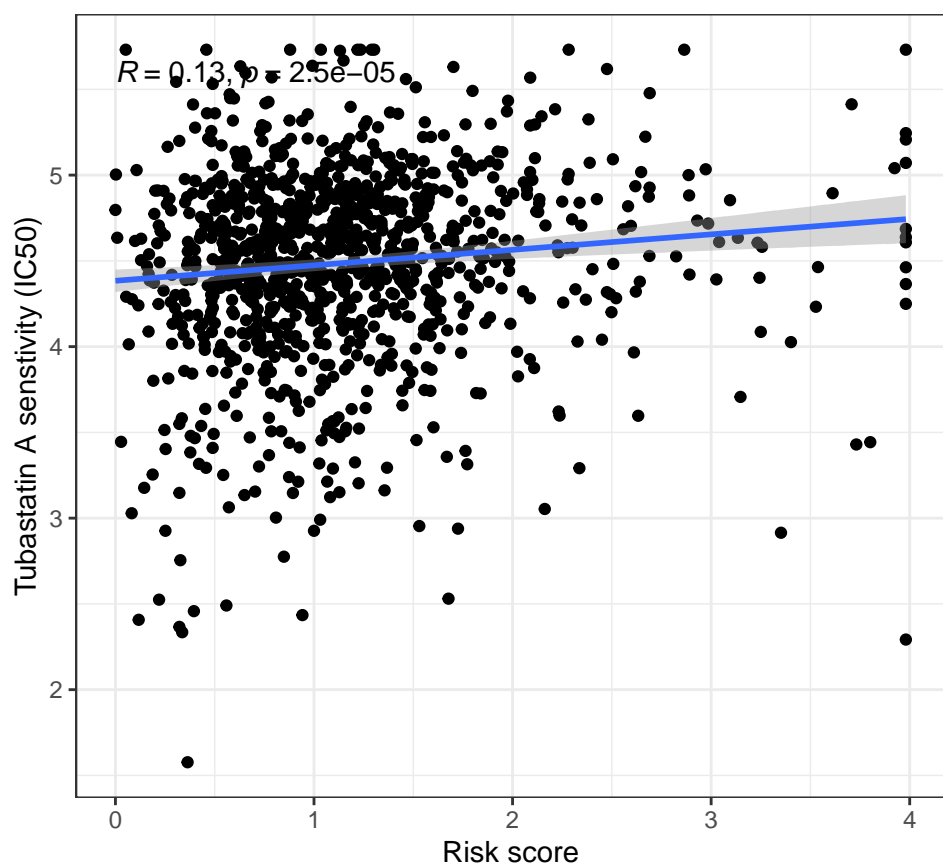

Supplement: Supplementary file 1 [file diagnostics-13-01203-s001.zip › Figure S3/Cor.Tubastatin A.pdf]

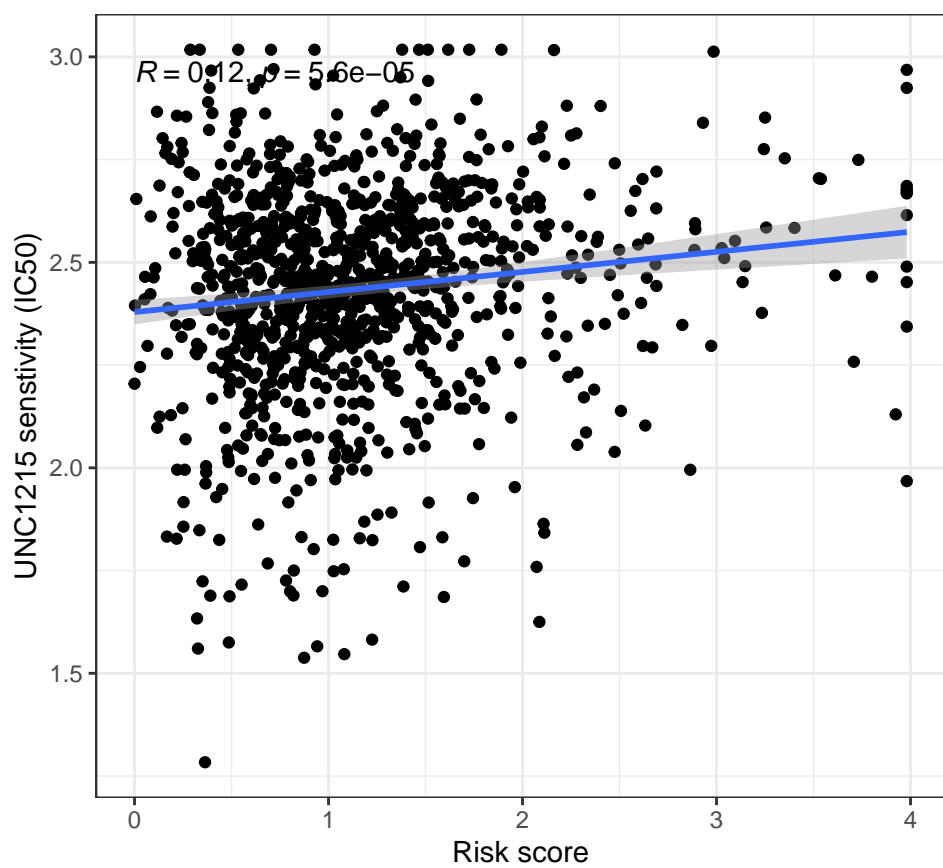

Supplement: Supplementary file 1 [file diagnostics-13-01203-s001.zip › Figure S3/Cor.UNC1215.pdf]

VX-11e sensitivity (IC50)

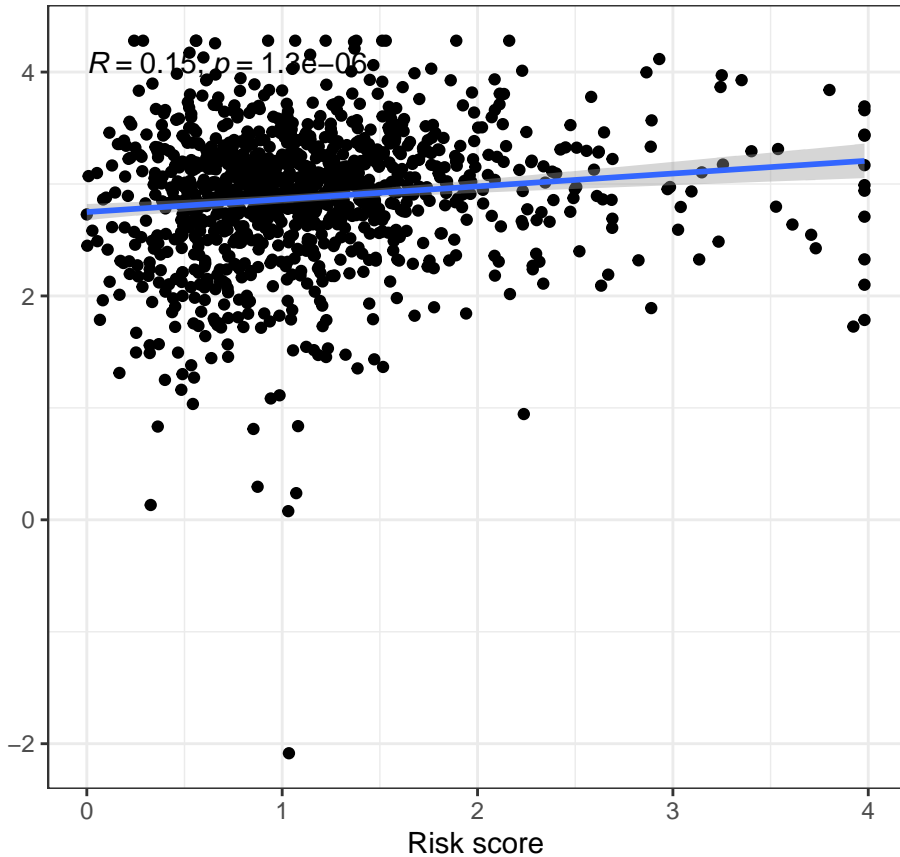

Supplement: Supplementary file 1 [file diagnostics-13-01203-s001.zip › Figure S3/Cor.VX-11e.pdf]

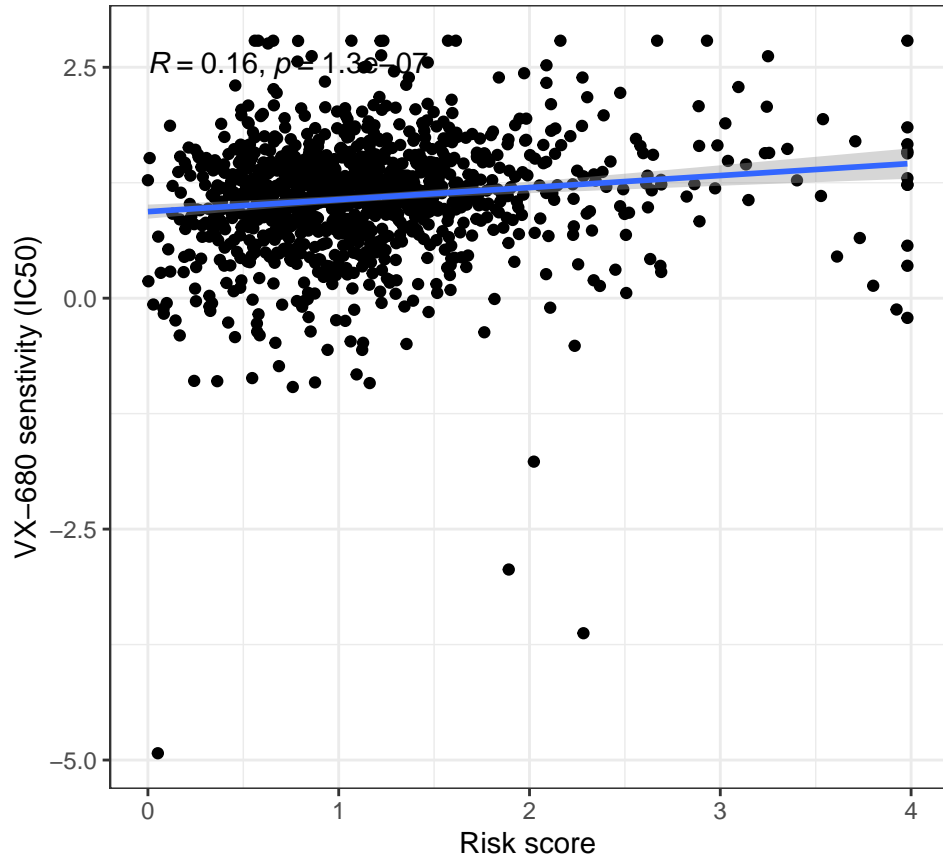

Supplement: Supplementary file 1 [file diagnostics-13-01203-s001.zip › Figure S3/Cor.VX-680.pdf]

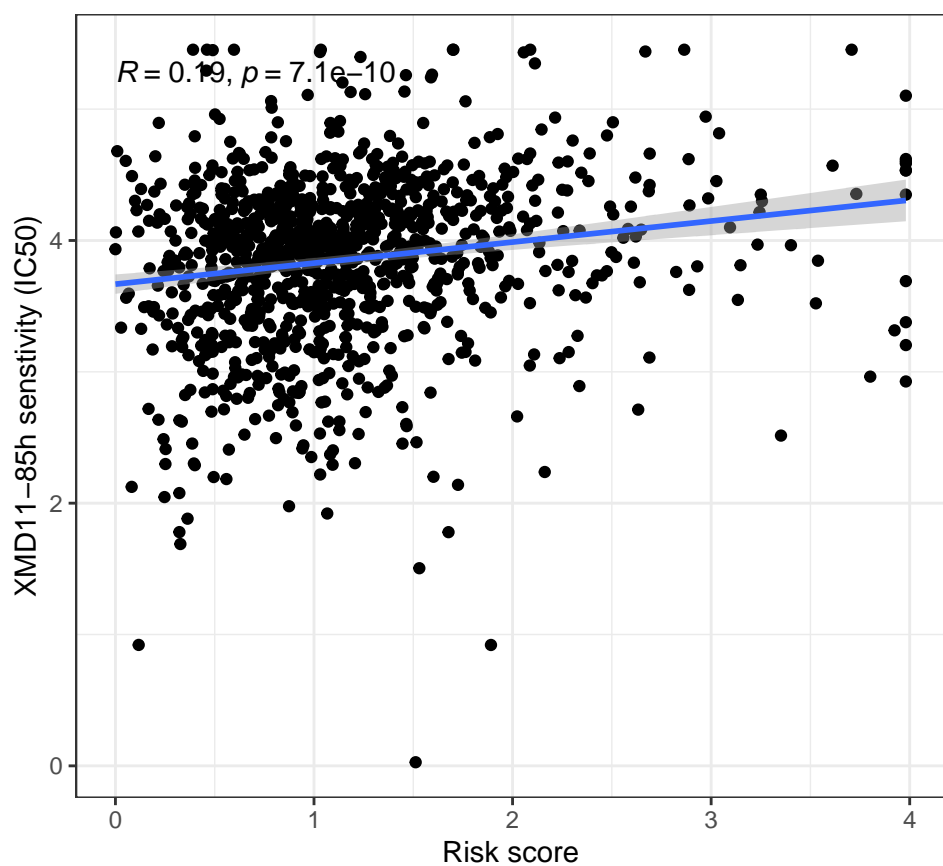

Supplement: Supplementary file 1 [file diagnostics-13-01203-s001.zip › Figure S3/Cor.XMD11-85h.pdf]

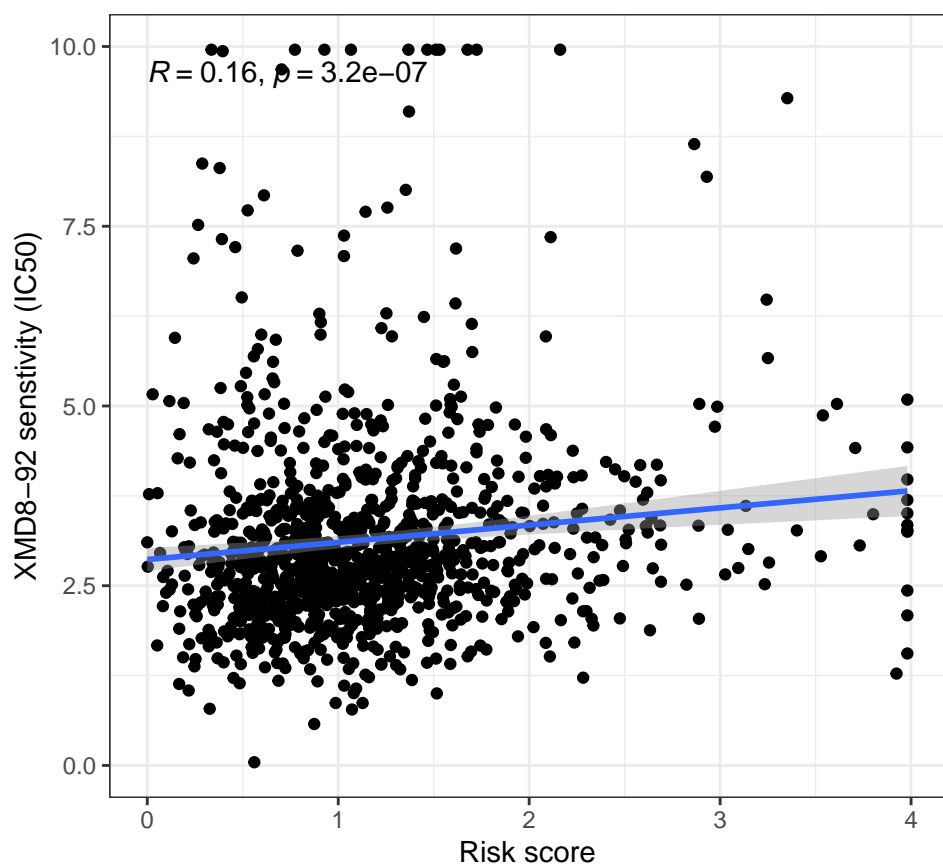

Supplement: Supplementary file 1 [file diagnostics-13-01203-s001.zip › Figure S3/Cor.XMD8-92.pdf]

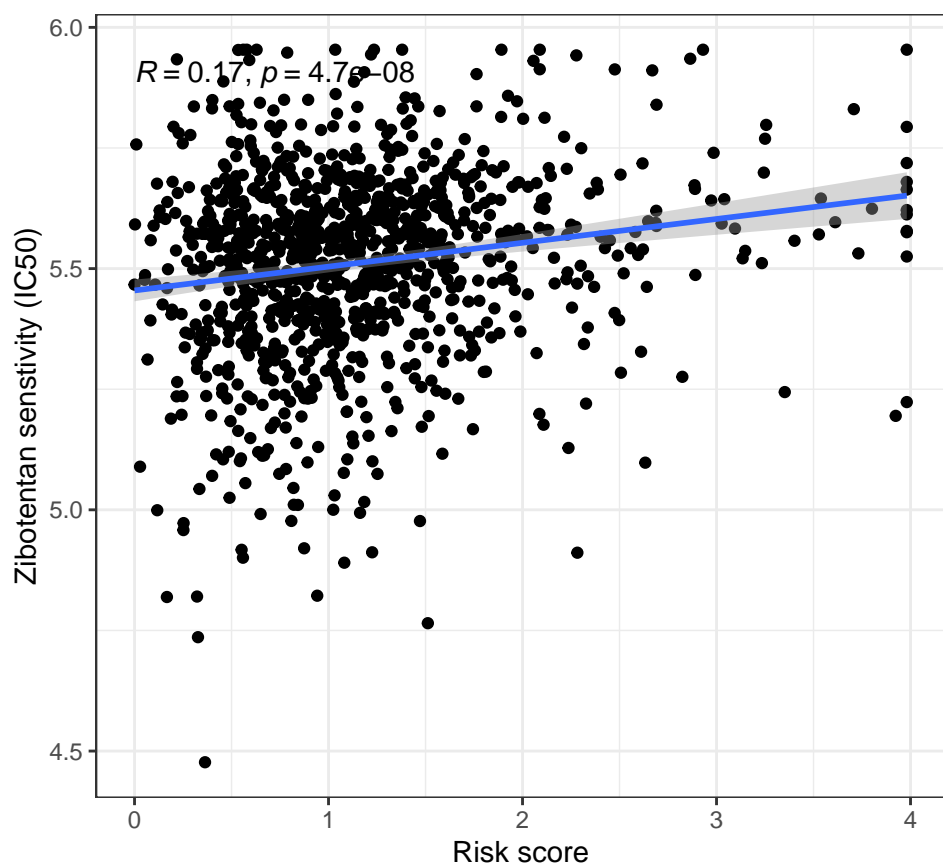

Supplement: Supplementary file 1 [file diagnostics-13-01203-s001.zip › Figure S3/Cor.Zibotentan.pdf]

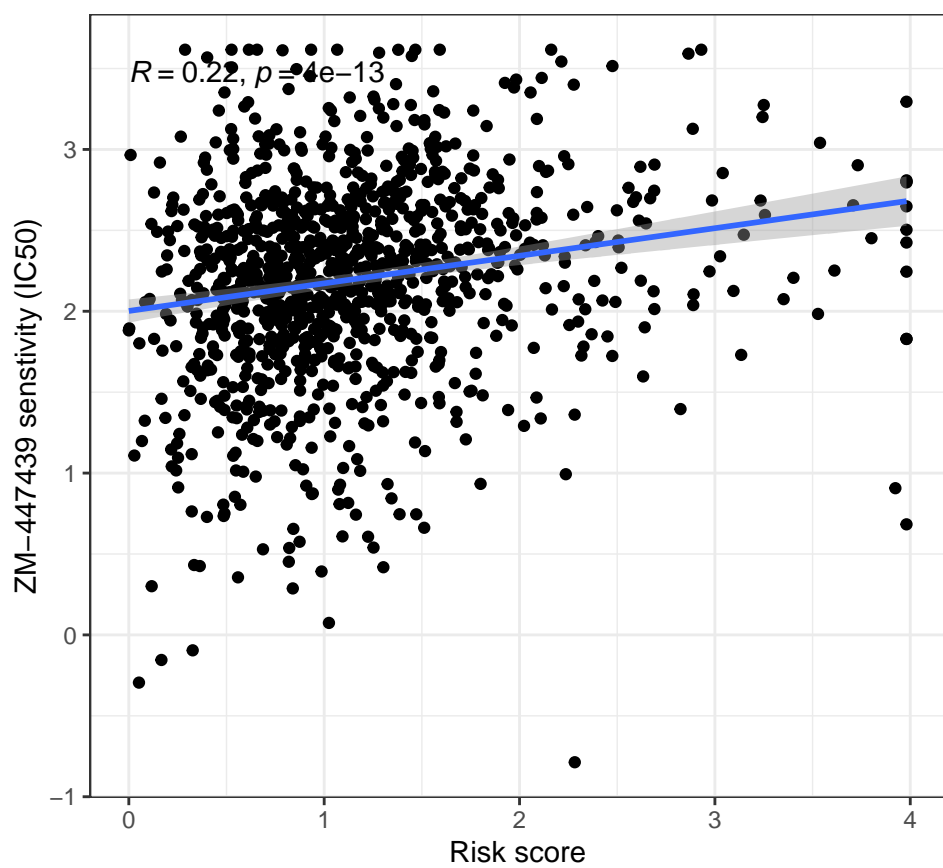

Supplement: Supplementary file 1 [file diagnostics-13-01203-s001.zip › Figure S3/Cor.ZM-447439.pdf]

Risk 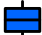 low 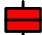 high

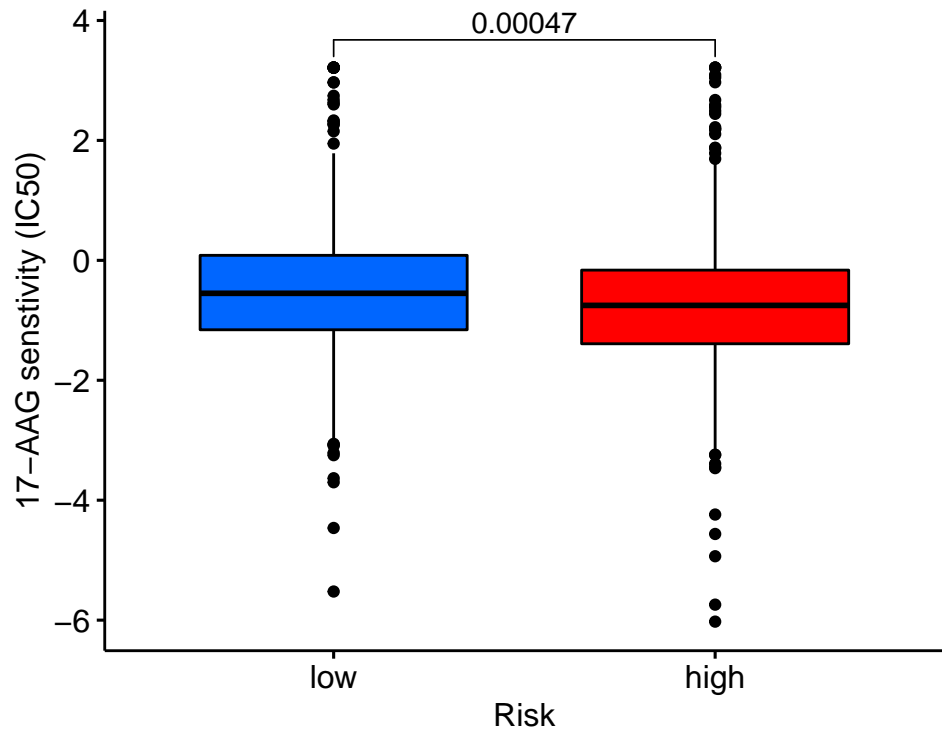

Supplement: Supplementary file 1 [file diagnostics-13-01203-s001.zip › Figure S3/durgSenstivity.17-AAG.pdf]

Risk 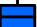 low 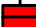 high

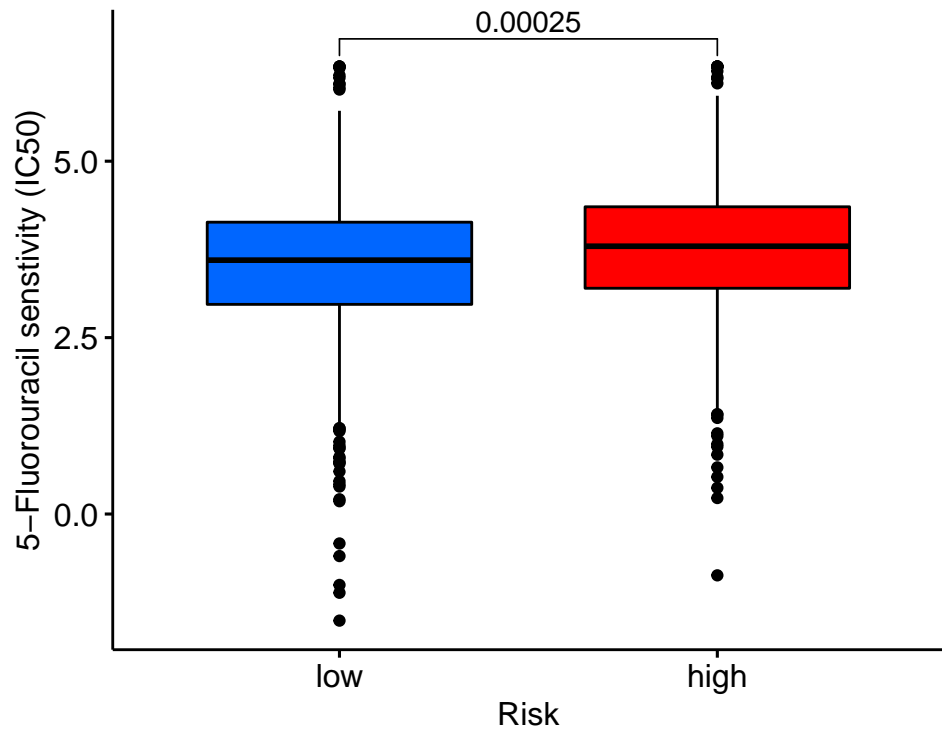

Supplement: Supplementary file 1 [file diagnostics-13-01203-s001.zip › Figure S3/durgSenstivity.5-Fluorouracil.pdf]

Risk 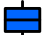 low 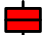 high

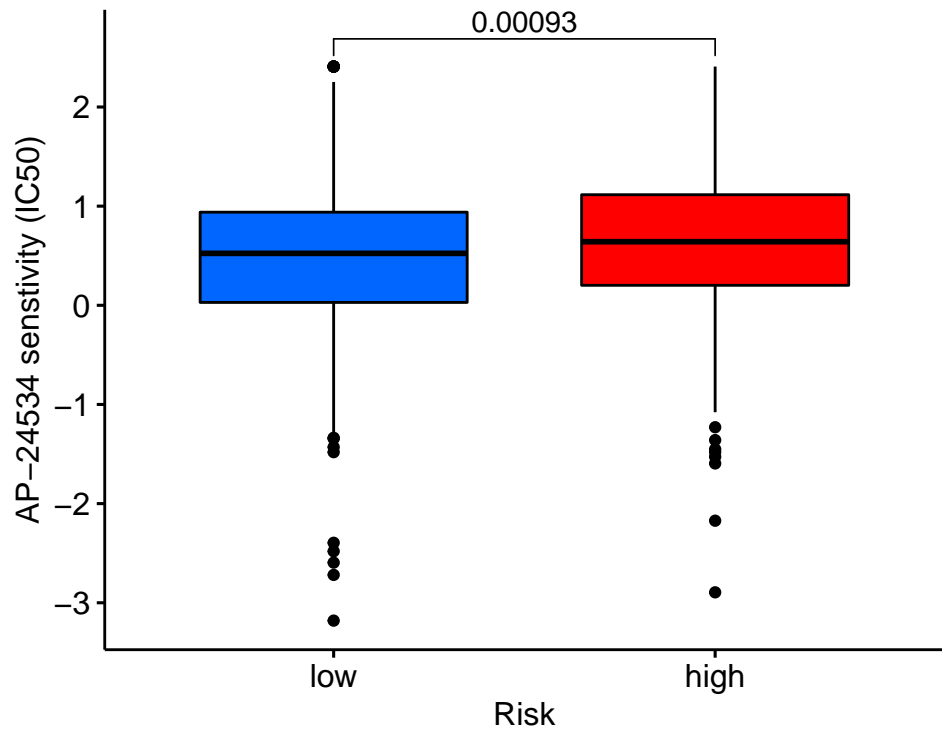

Supplement: Supplementary file 1 [file diagnostics-13-01203-s001.zip › Figure S3/durgSenstivity.AP-24534.pdf]

Risk 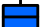 low 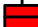 high

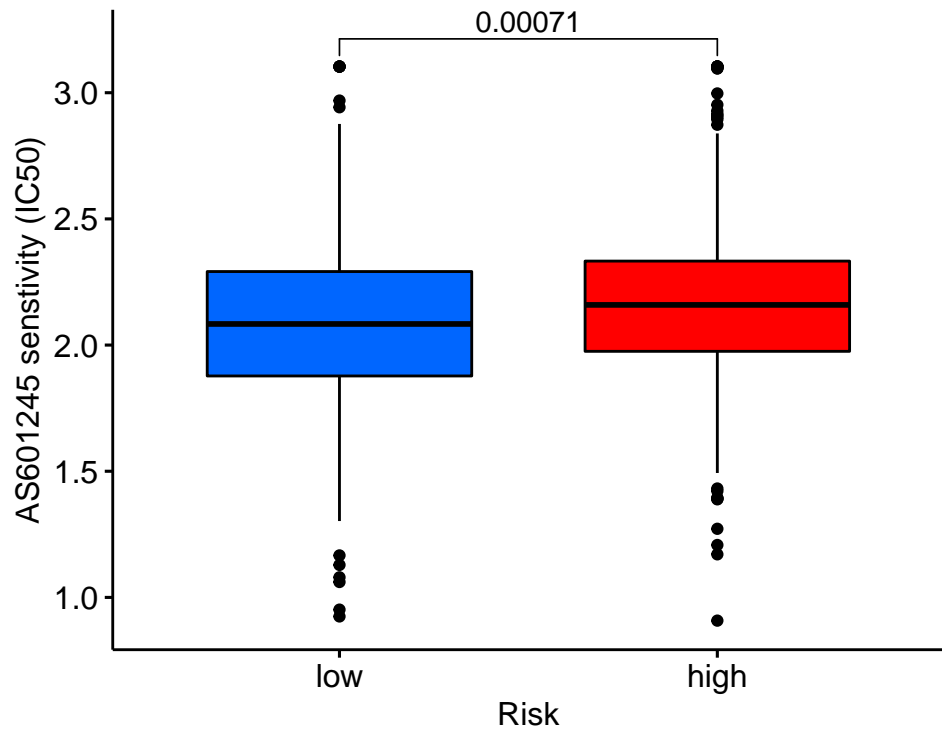

Supplement: Supplementary file 1 [file diagnostics-13-01203-s001.zip › Figure S3/durgSenstivity.AS601245.pdf]

Risk 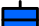 low 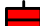 high

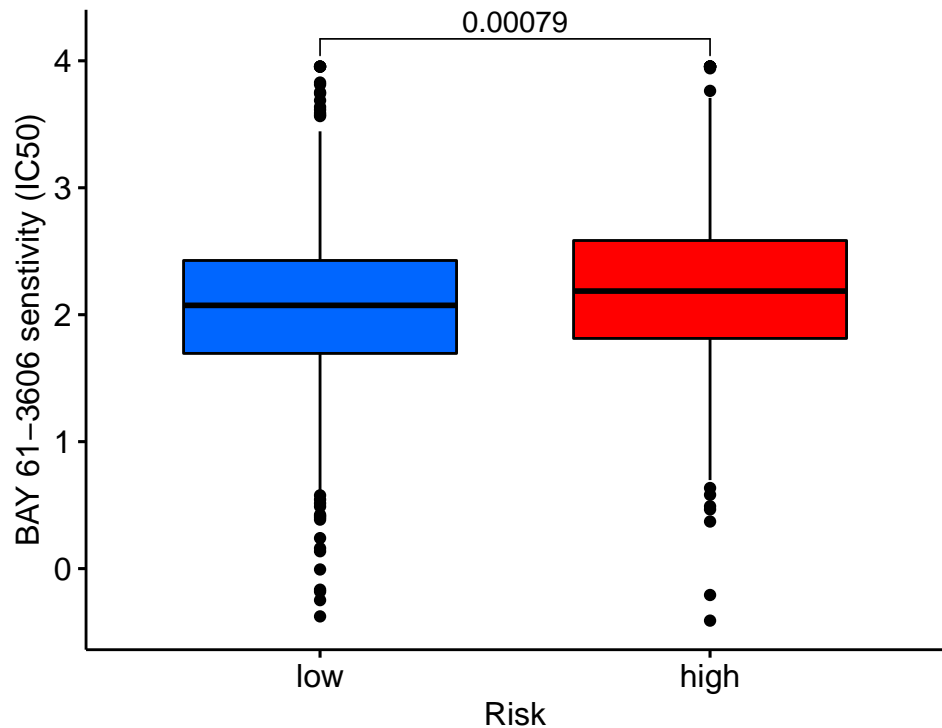

Supplement: Supplementary file 1 [file diagnostics-13-01203-s001.zip › Figure S3/durgSenstivity.BAY 61-3606.pdf]

Risk 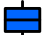 low 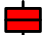 high

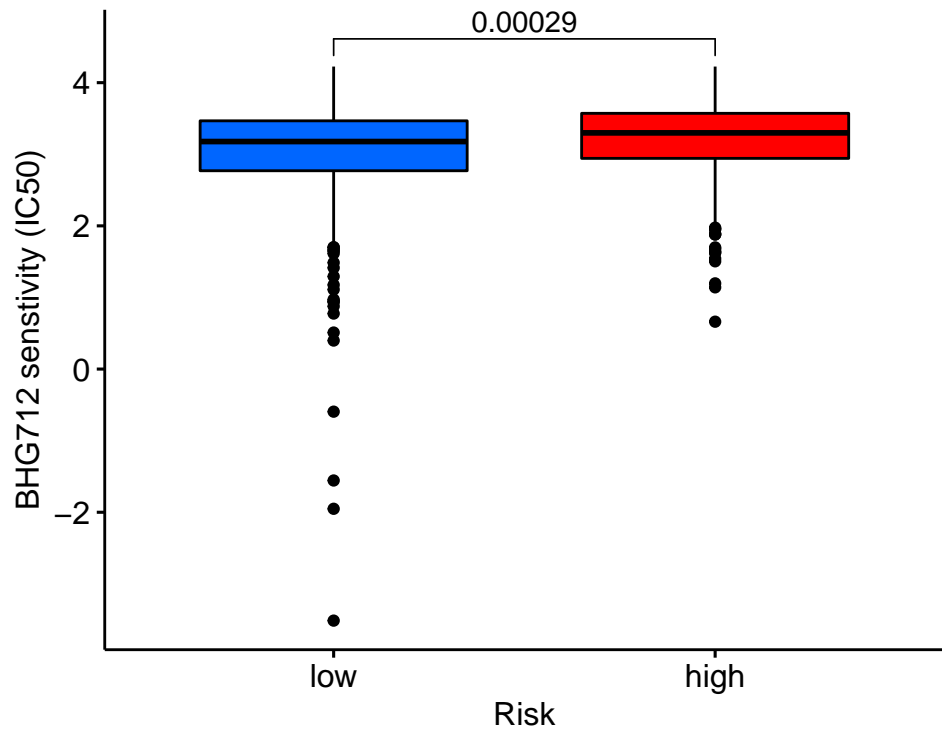

Supplement: Supplementary file 1 [file diagnostics-13-01203-s001.zip › Figure S3/durgSenstivity.BHG712.pdf]

Risk 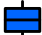 low 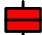 high

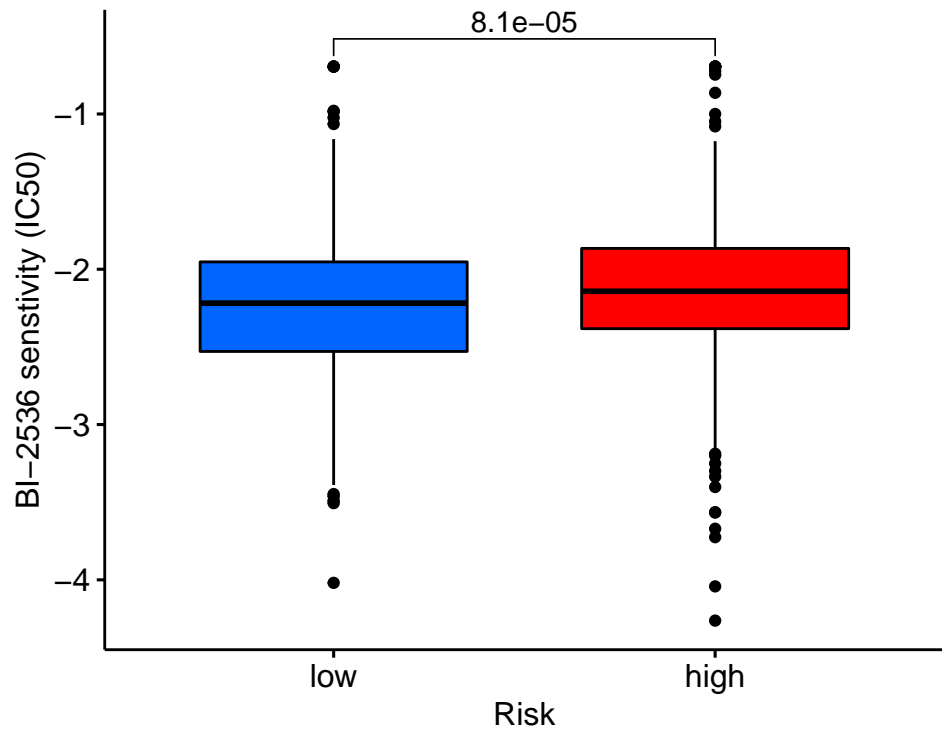

Supplement: Supplementary file 1 [file diagnostics-13-01203-s001.zip › Figure S3/durgSenstivity.BI-2536.pdf]

Risk 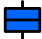 low 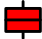 high

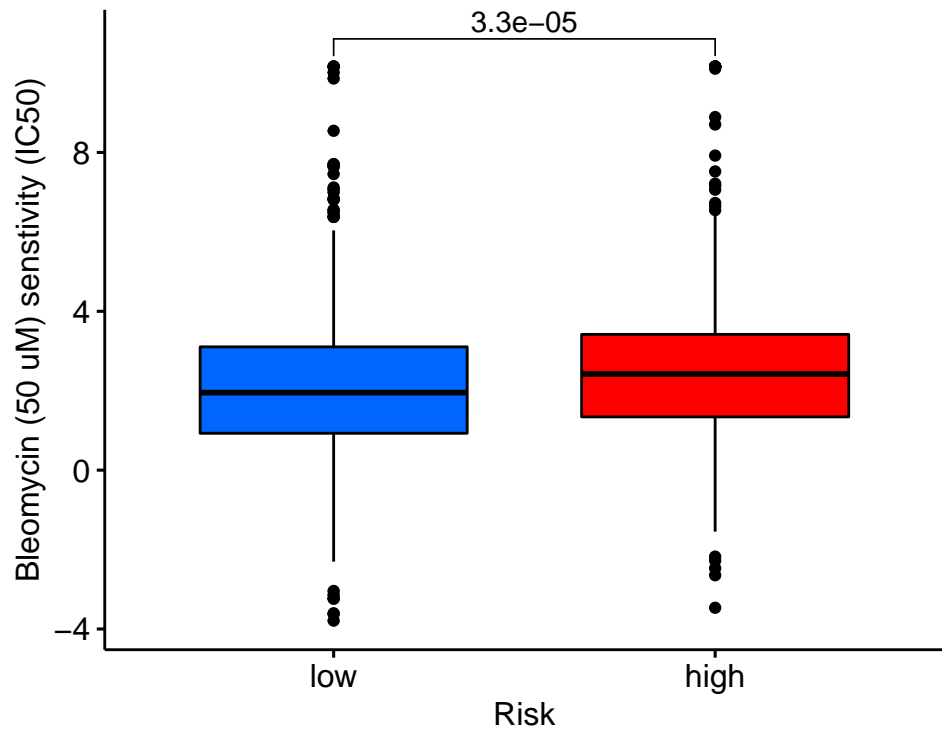

Supplement: Supplementary file 1 [file diagnostics-13-01203-s001.zip › Figure S3/durgSenstivity.Bleomycin (50 uM).pdf]

BMS-754807 sensitivity (IC50)

Risk low high

0.00032

low

high

Risk

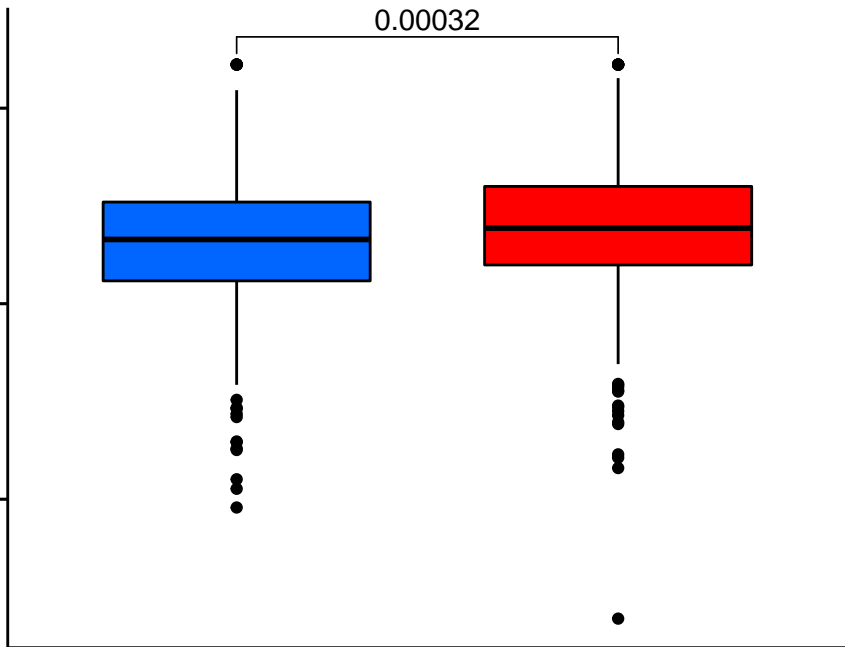

Supplement: Supplementary file 1 [file diagnostics-13-01203-s001.zip › Figure S3/durgSenstivity.BMS-754807.pdf]

Risk 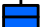 low 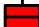 high

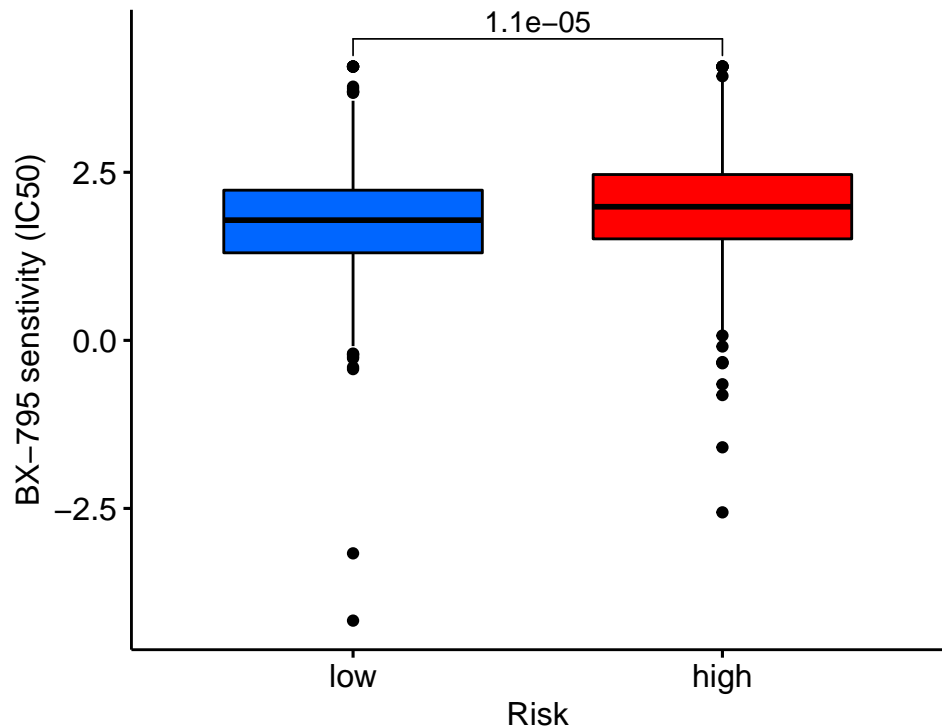

Supplement: Supplementary file 1 [file diagnostics-13-01203-s001.zip › Figure S3/durgSenstivity.BX-795.pdf]

Risk 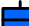 low 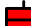 high

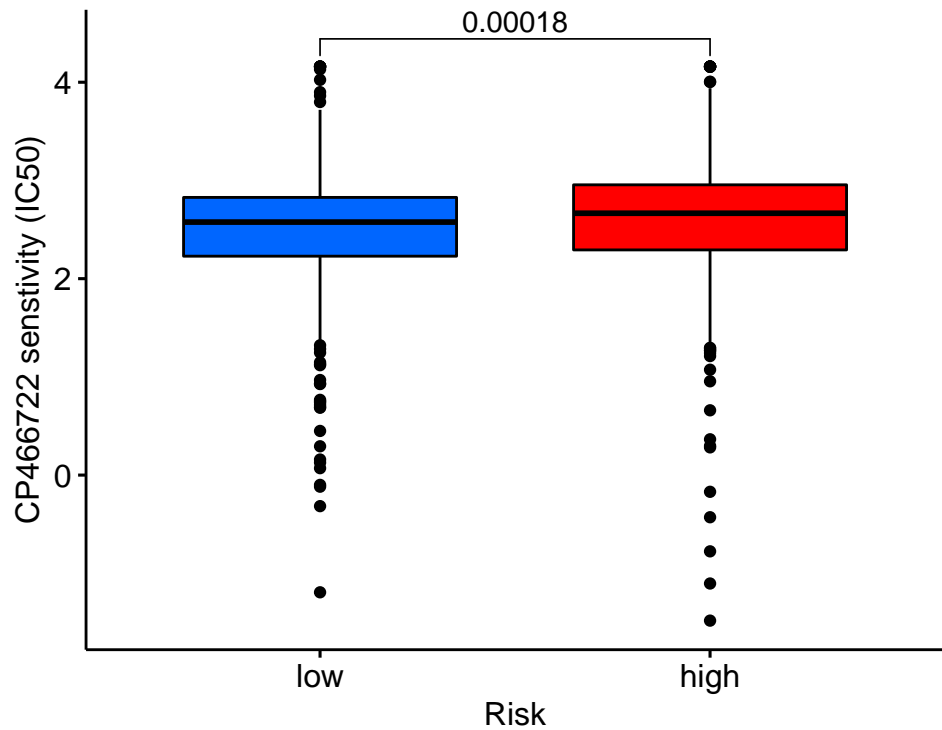

Supplement: Supplementary file 1 [file diagnostics-13-01203-s001.zip › Figure S3/durgSenstivity.CP466722.pdf]

Risk 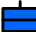 low 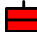 high

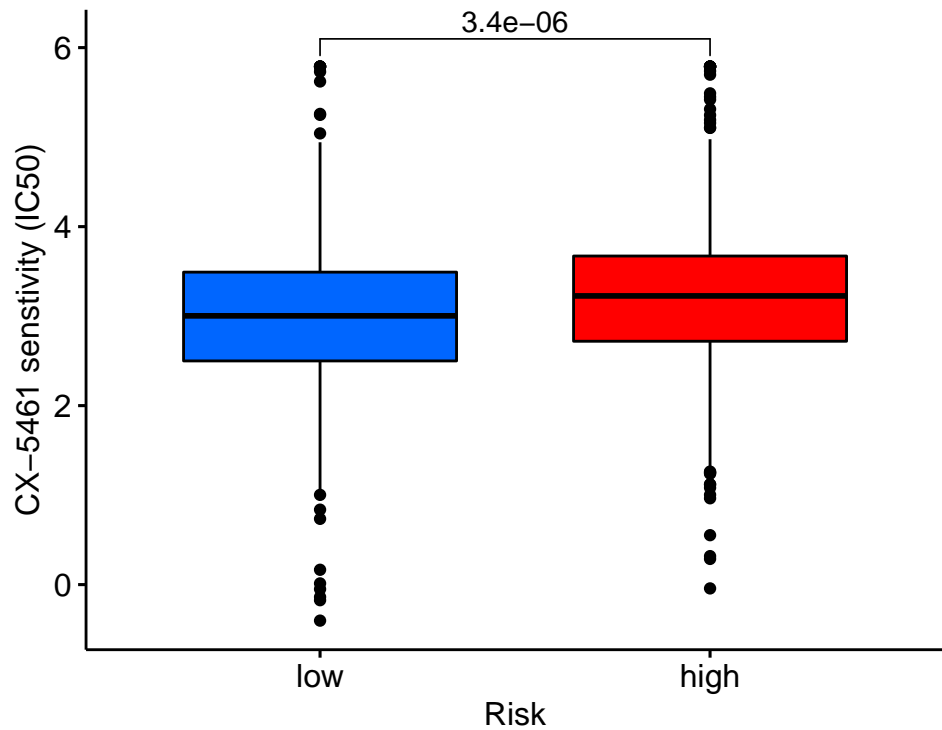

Supplement: Supplementary file 1 [file diagnostics-13-01203-s001.zip › Figure S3/durgSenstivity.CX-5461.pdf]

Risk 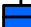 low 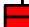 high

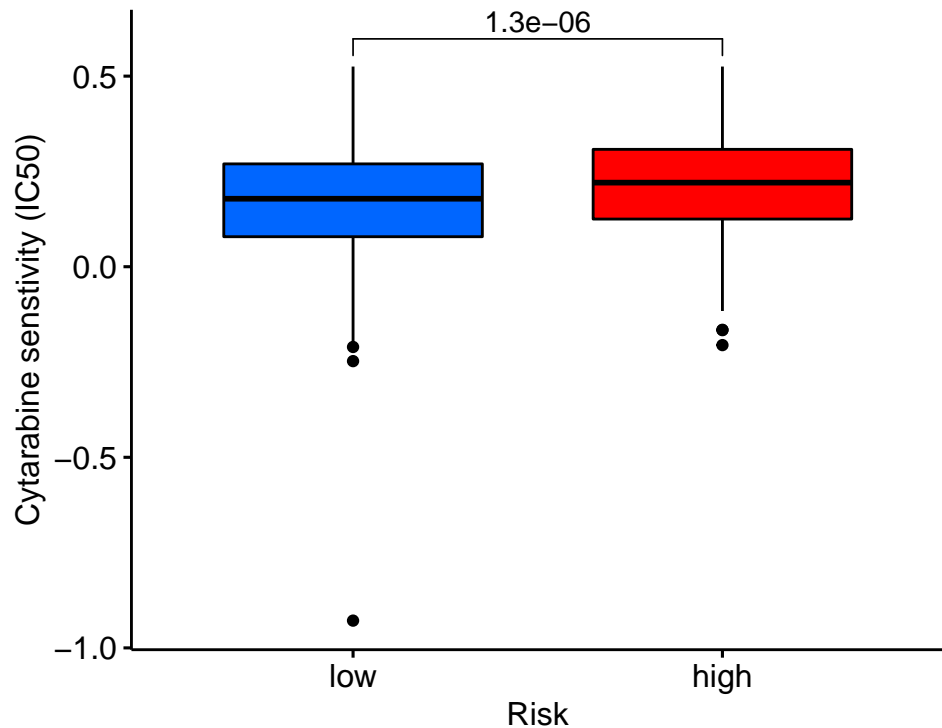

Supplement: Supplementary file 1 [file diagnostics-13-01203-s001.zip › Figure S3/durgSenstivity.Cytarabine.pdf]

Risk 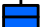 low 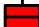 high

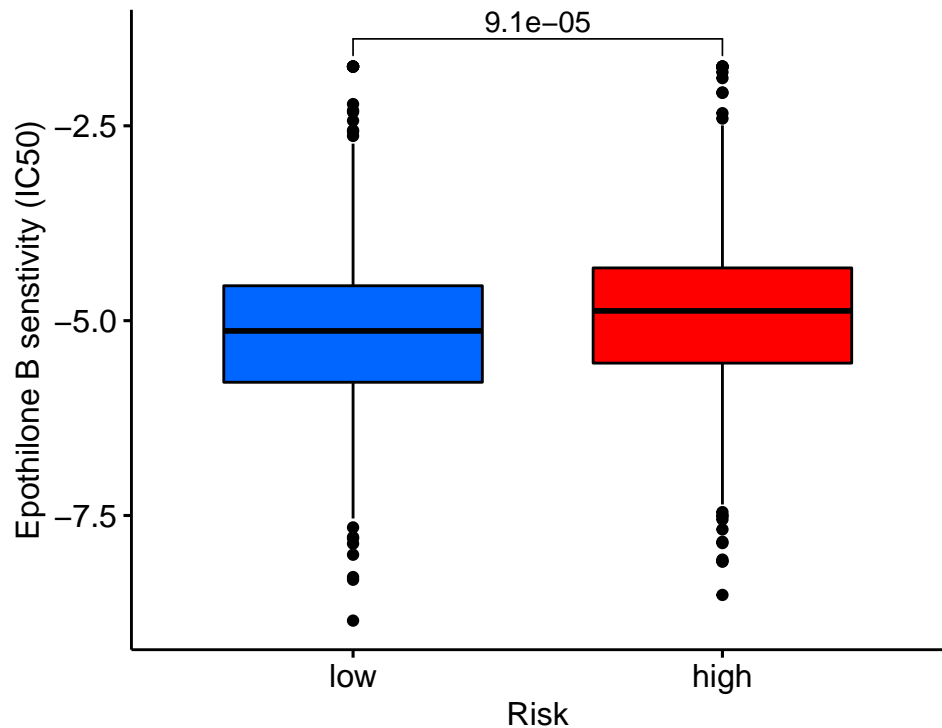

Supplement: Supplementary file 1 [file diagnostics-13-01203-s001.zip › Figure S3/durgSenstivity.Epothilone B.pdf]

Risk 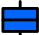 low 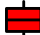 high

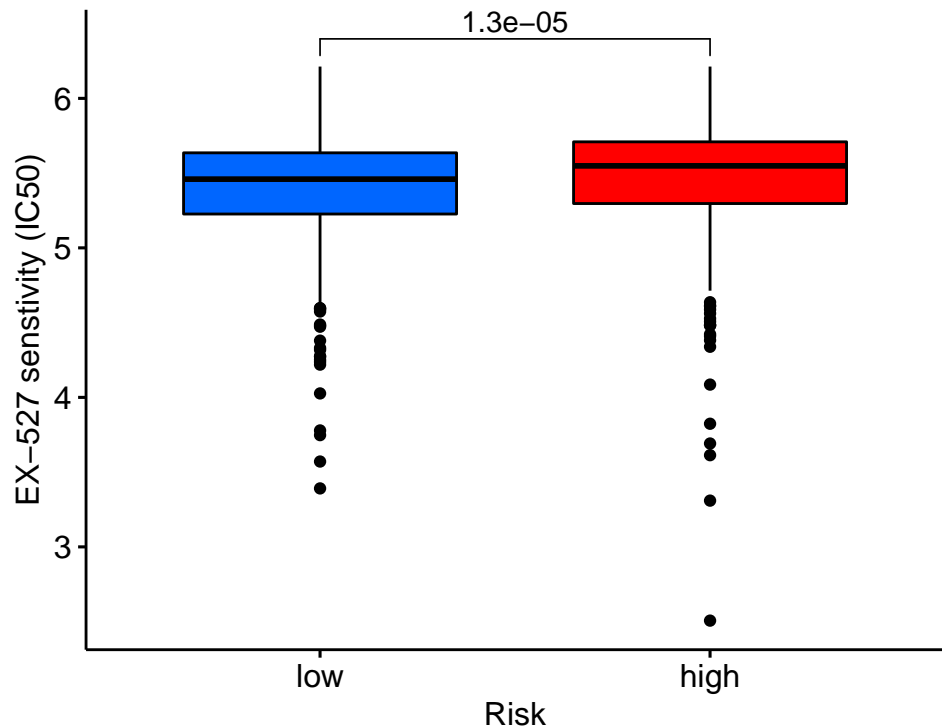

Supplement: Supplementary file 1 [file diagnostics-13-01203-s001.zip › Figure S3/durgSenstivity.EX-527.pdf]

Risk 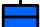 low 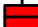 high

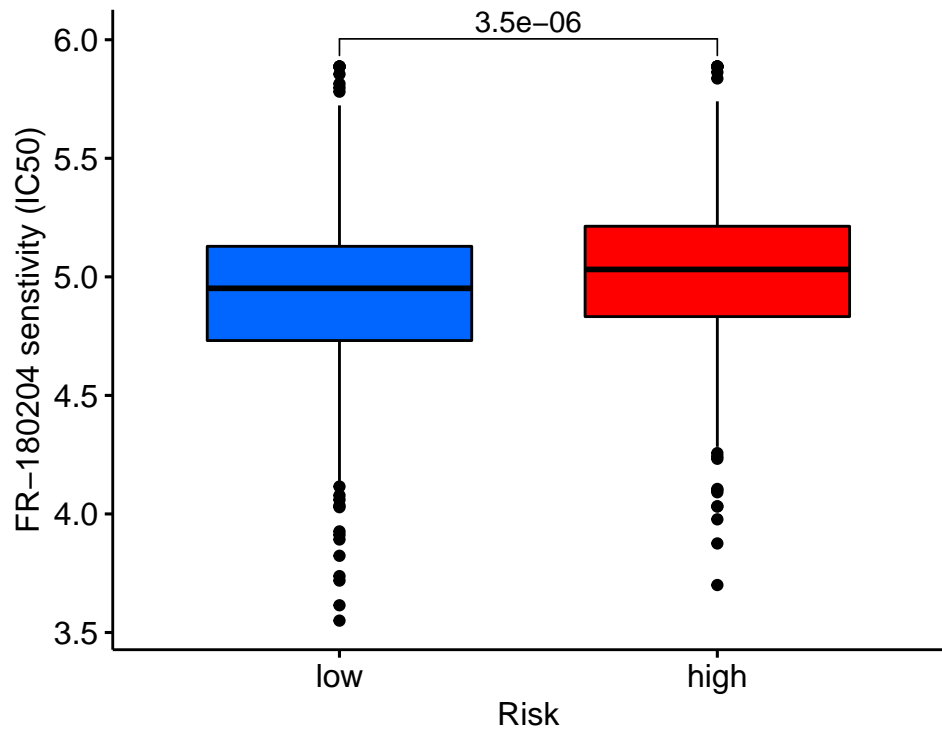

Supplement: Supplementary file 1 [file diagnostics-13-01203-s001.zip › Figure S3/durgSenstivity.FR-180204.pdf]

Risk 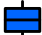 low 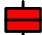 high

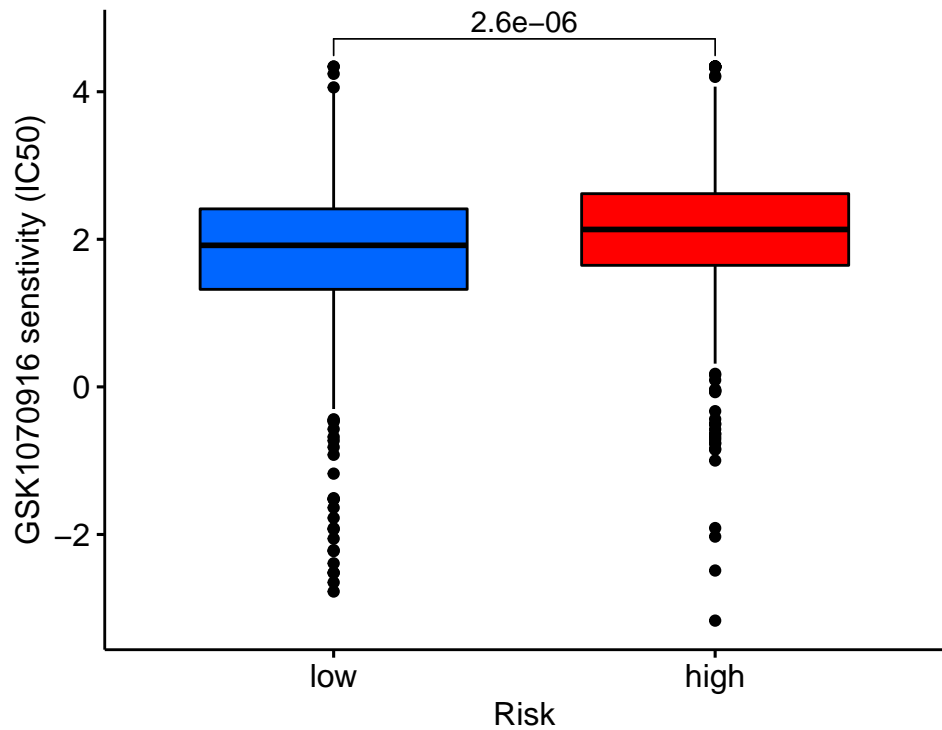

Supplement: Supplementary file 1 [file diagnostics-13-01203-s001.zip › Figure S3/durgSenstivity.GSK1070916.pdf]

Risk 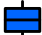 low 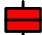 high

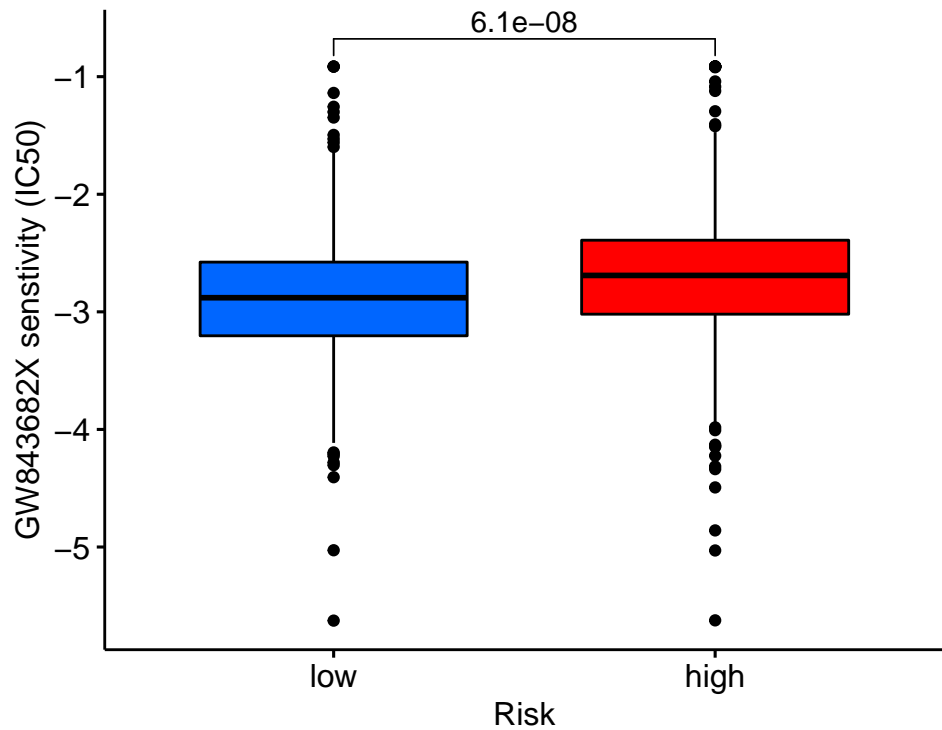

Supplement: Supplementary file 1 [file diagnostics-13-01203-s001.zip › Figure S3/durgSenstivity.GW843682X.pdf]

HG-5-113-01 sensitivity (IC50)

Risk 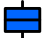 low 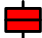 high

$3.1\text{e-}05$

low

high

Risk

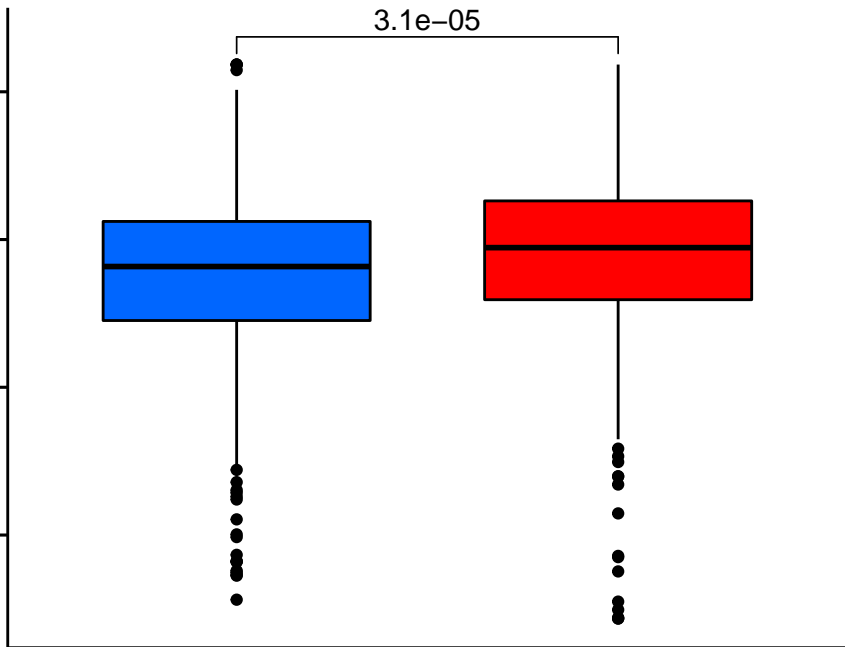

Supplement: Supplementary file 1 [file diagnostics-13-01203-s001.zip › Figure S3/durgSenstivity.HG-5-113-01.pdf]

Risk 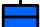 low 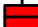 high

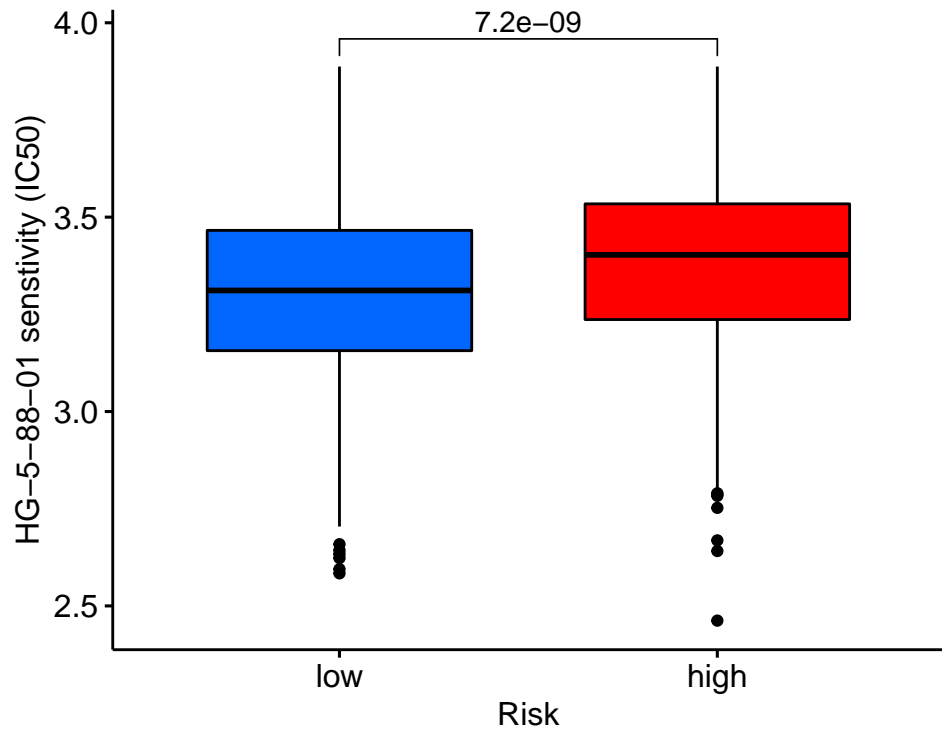

Supplement: Supplementary file 1 [file diagnostics-13-01203-s001.zip › Figure S3/durgSenstivity.HG-5-88-01.pdf]

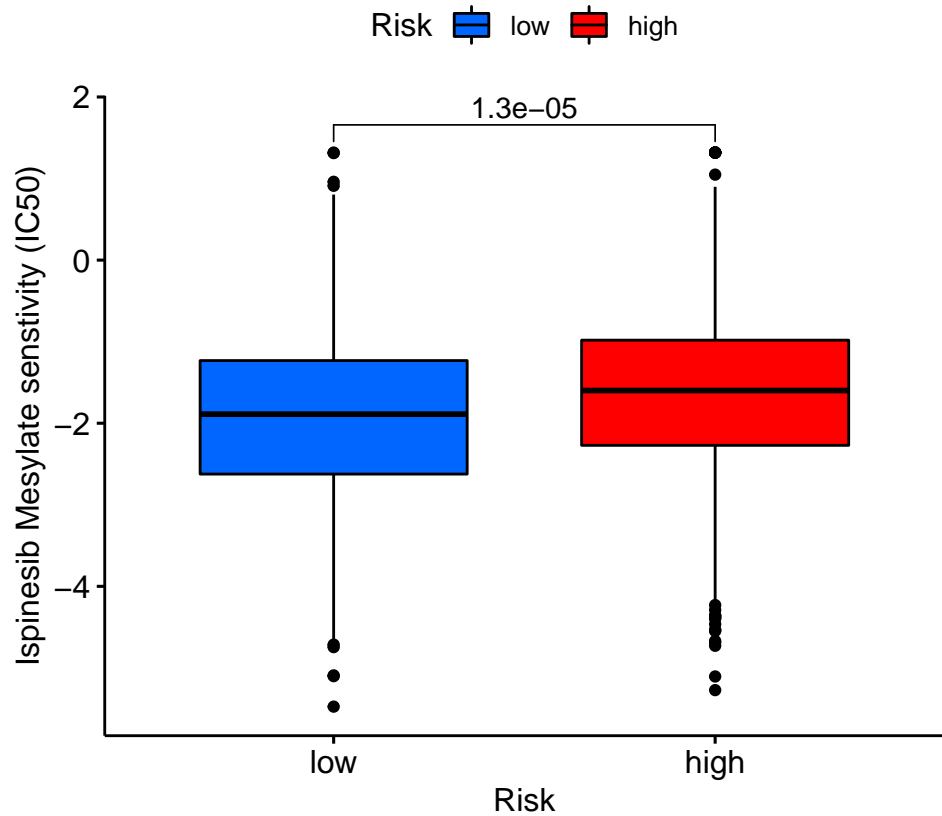

Supplement: Supplementary file 1 [file diagnostics-13-01203-s001.zip › Figure S3/durgSenstivity.Ispinesib Mesylate.pdf]

Risk 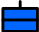 low 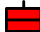 high

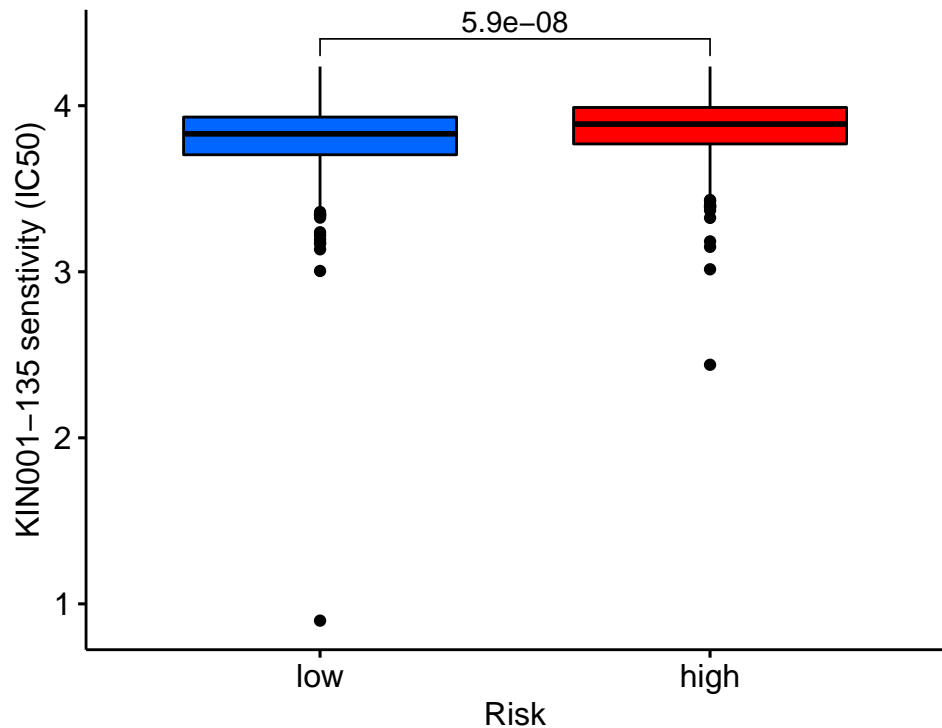

Supplement: Supplementary file 1 [file diagnostics-13-01203-s001.zip › Figure S3/durgSenstivity.KIN001-135.pdf]

Risk 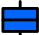 low 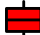 high

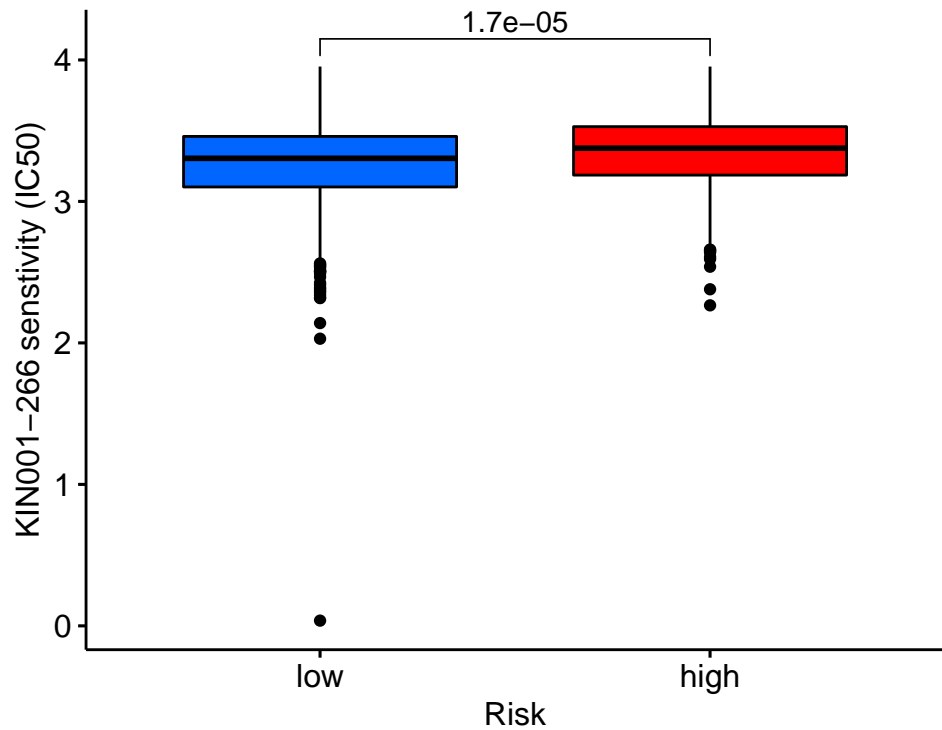

Supplement: Supplementary file 1 [file diagnostics-13-01203-s001.zip › Figure S3/durgSenstivity.KIN001-266.pdf]

Risk 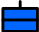 low 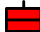 high

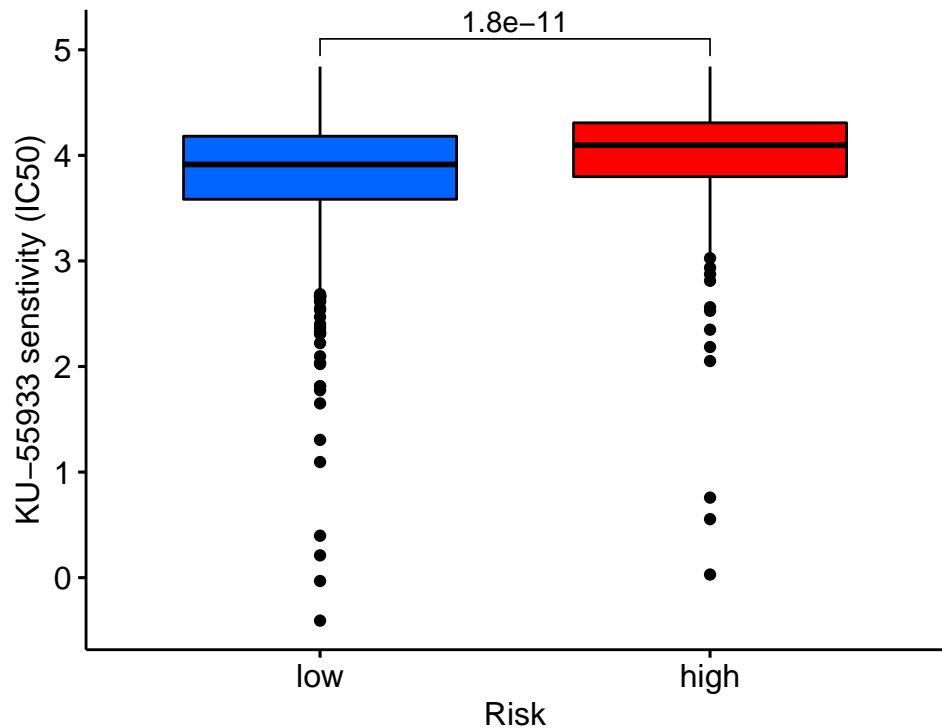

Supplement: Supplementary file 1 [file diagnostics-13-01203-s001.zip › Figure S3/durgSenstivity.KU-55933.pdf]

Risk 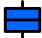 low 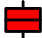 high

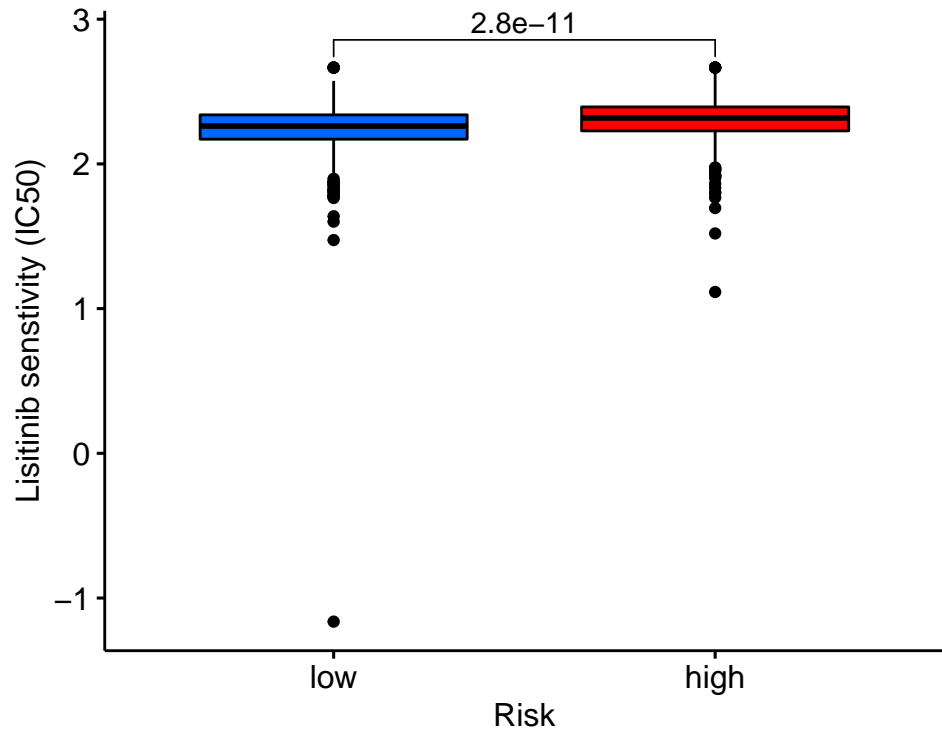

Supplement: Supplementary file 1 [file diagnostics-13-01203-s001.zip › Figure S3/durgSenstivity.Lisitinib.pdf]

Risk 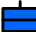 low 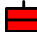 high

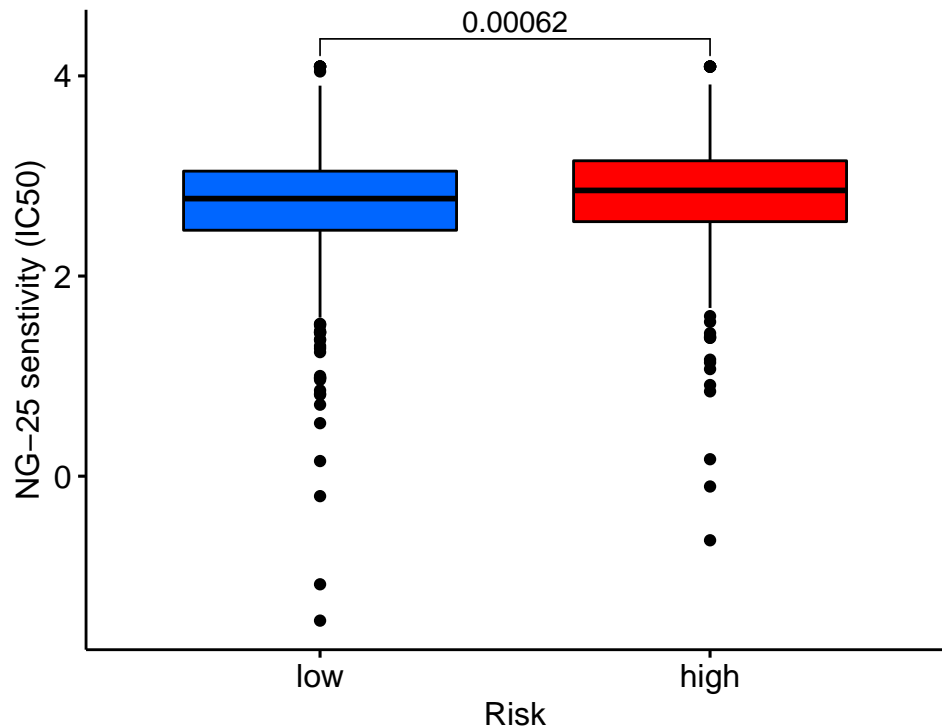

Supplement: Supplementary file 1 [file diagnostics-13-01203-s001.zip › Figure S3/durgSenstivity.NG-25.pdf]

Risk 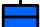 low 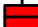 high

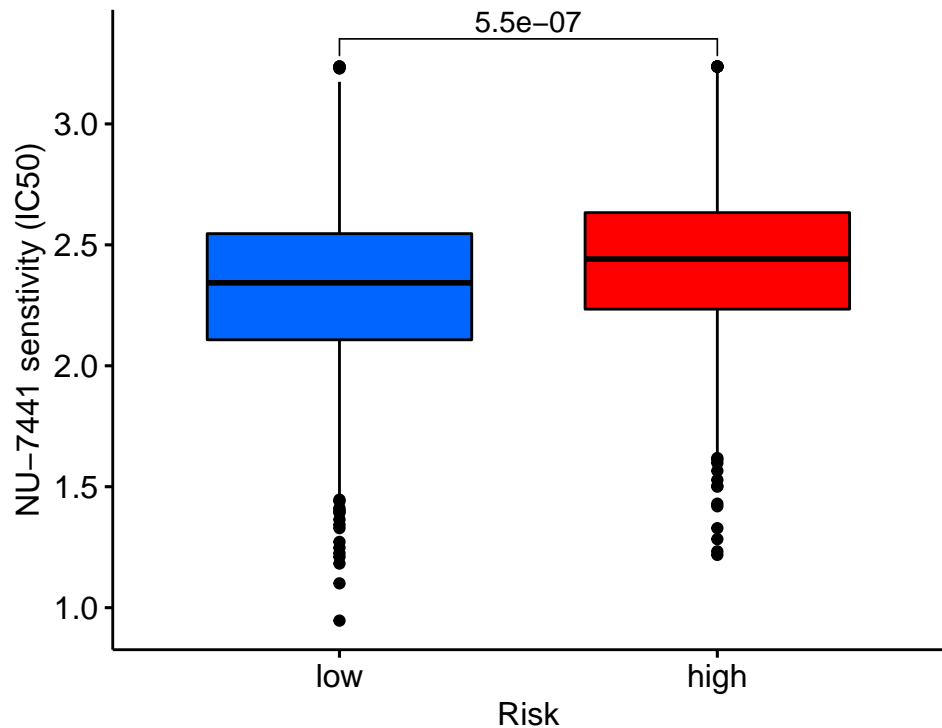

Supplement: Supplementary file 1 [file diagnostics-13-01203-s001.zip › Figure S3/durgSenstivity.NU-7441.pdf]

PD-0332991 sensitivity (IC50)

Risk 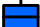 low 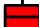 high

0.00012

low

high

Risk

2.5

0.0

-2.5

Supplement: Supplementary file 1 [file diagnostics-13-01203-s001.zip › Figure S3/durgSenstivity.PD-0332991.pdf]

Risk 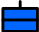 low 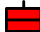 high

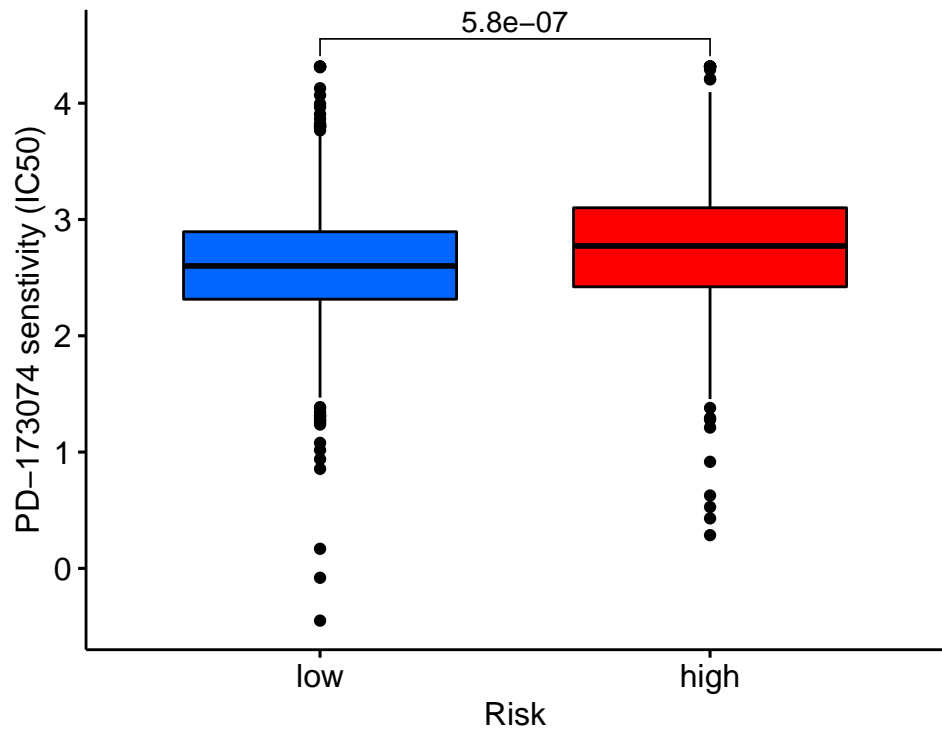

Supplement: Supplementary file 1 [file diagnostics-13-01203-s001.zip › Figure S3/durgSenstivity.PD-173074.pdf]

Risk 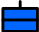 low 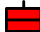 high

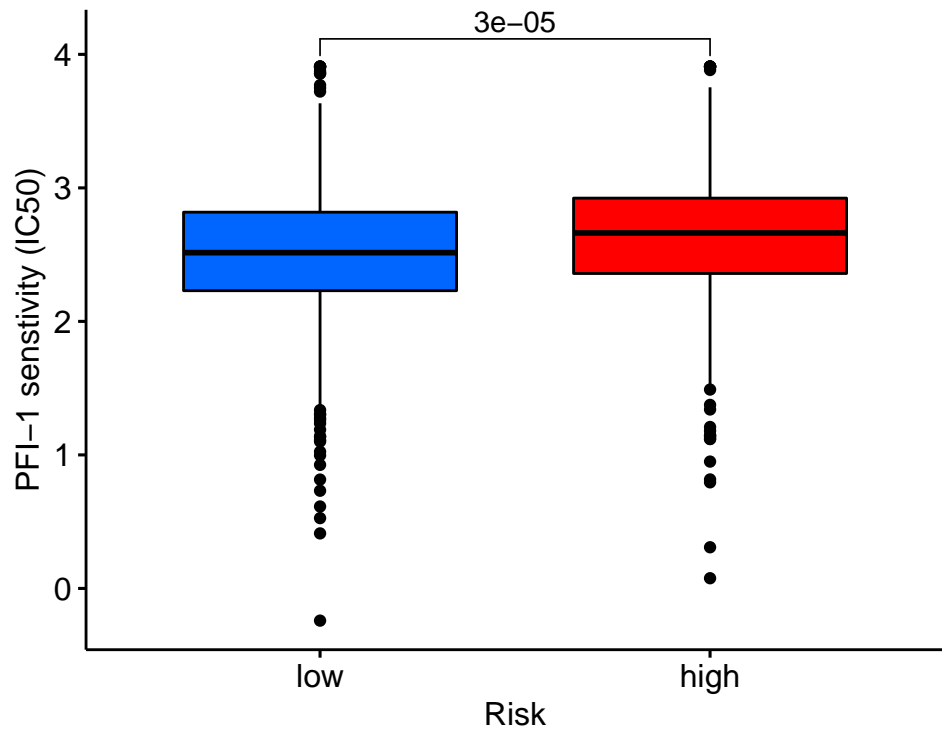

Supplement: Supplementary file 1 [file diagnostics-13-01203-s001.zip › Figure S3/durgSenstivity.PFI-1.pdf]

Risk 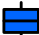 low 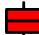 high

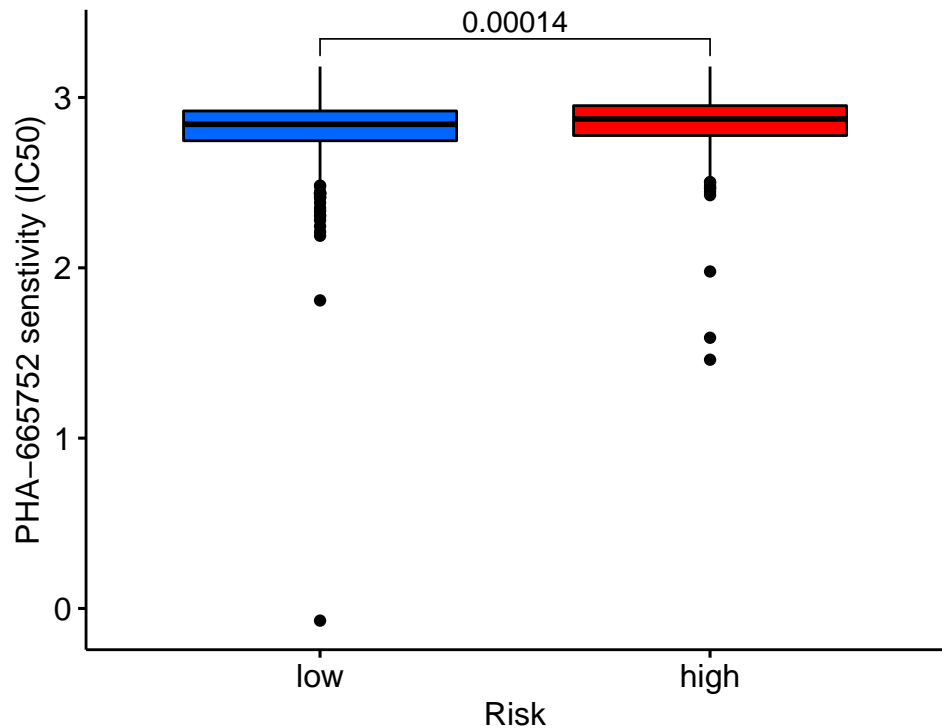

Supplement: Supplementary file 1 [file diagnostics-13-01203-s001.zip › Figure S3/durgSenstivity.PHA-665752.pdf]

Risk 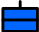 low 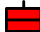 high

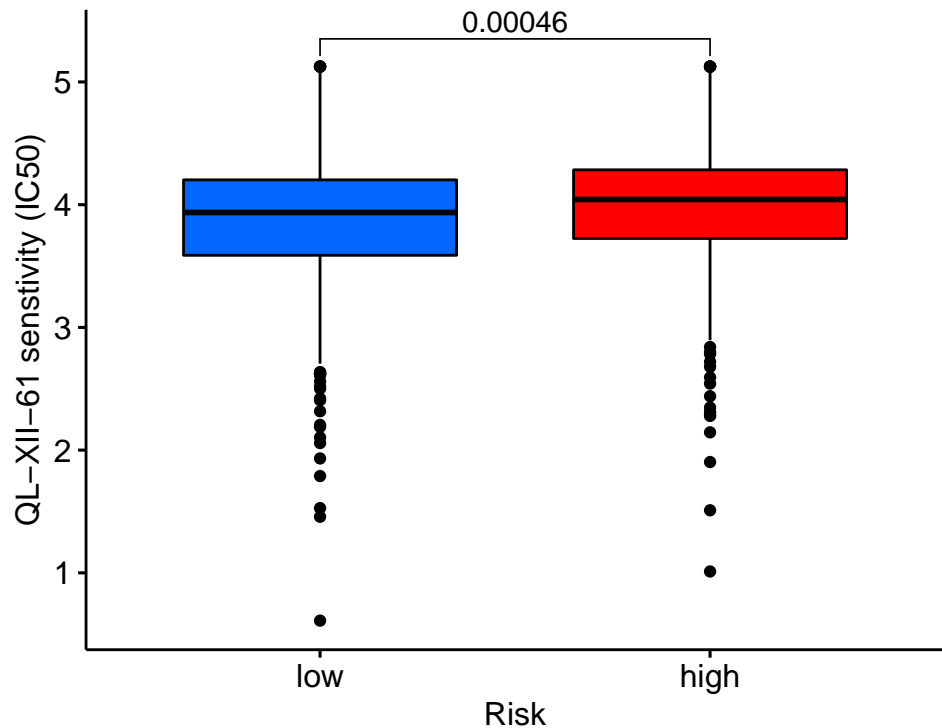

Supplement: Supplementary file 1 [file diagnostics-13-01203-s001.zip › Figure S3/durgSenstivity.QL-XII-61.pdf]

Risk 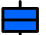 low 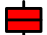 high

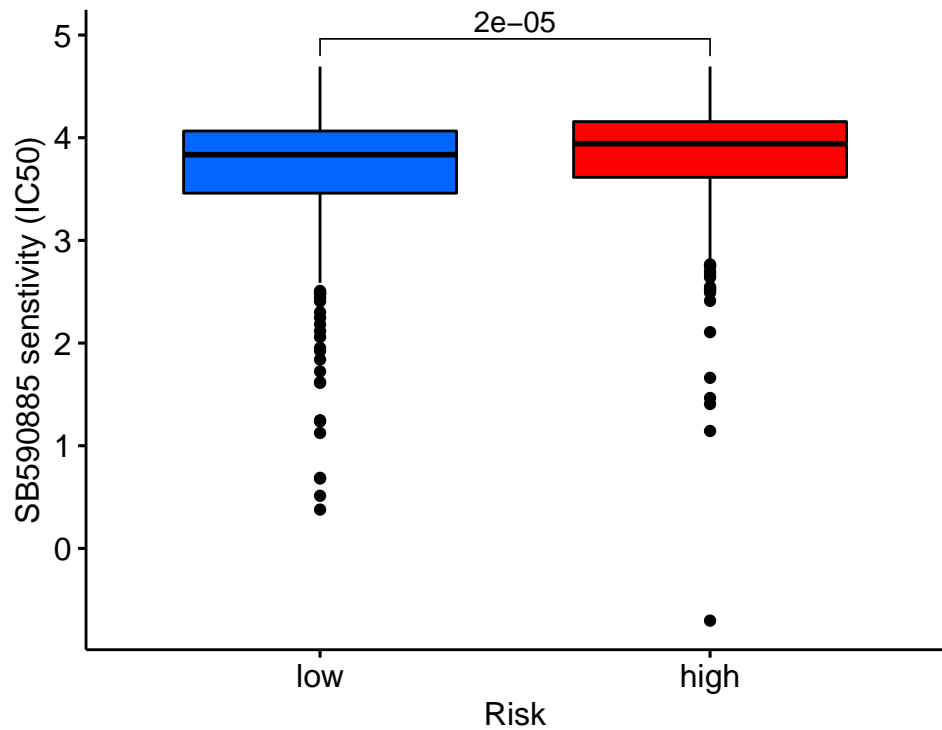

Supplement: Supplementary file 1 [file diagnostics-13-01203-s001.zip › Figure S3/durgSenstivity.SB590885.pdf]

Risk 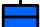 low 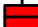 high

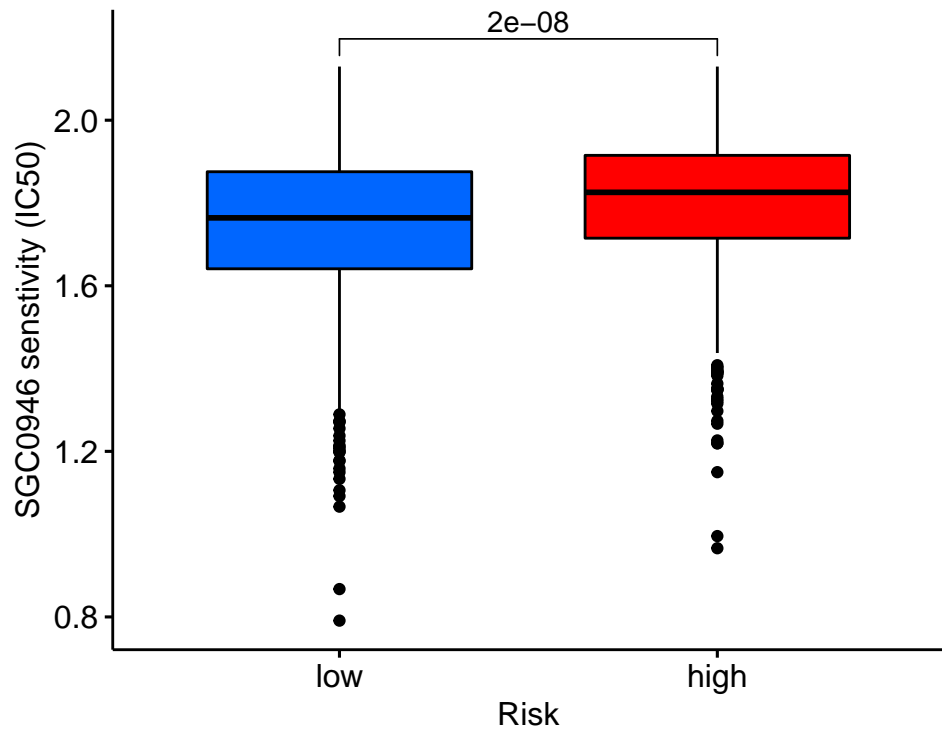

Supplement: Supplementary file 1 [file diagnostics-13-01203-s001.zip › Figure S3/durgSenstivity.SGC0946.pdf]

Risk 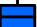 low 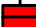 high

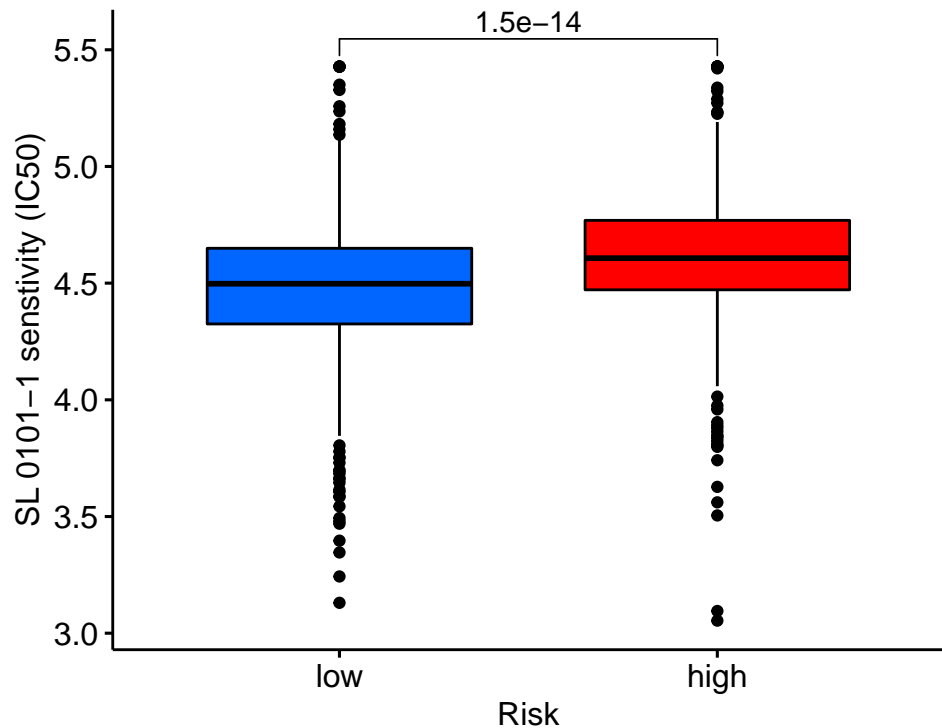

Supplement: Supplementary file 1 [file diagnostics-13-01203-s001.zip › Figure S3/durgSenstivity.SL 0101-1.pdf]

Risk 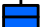 low 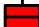 high

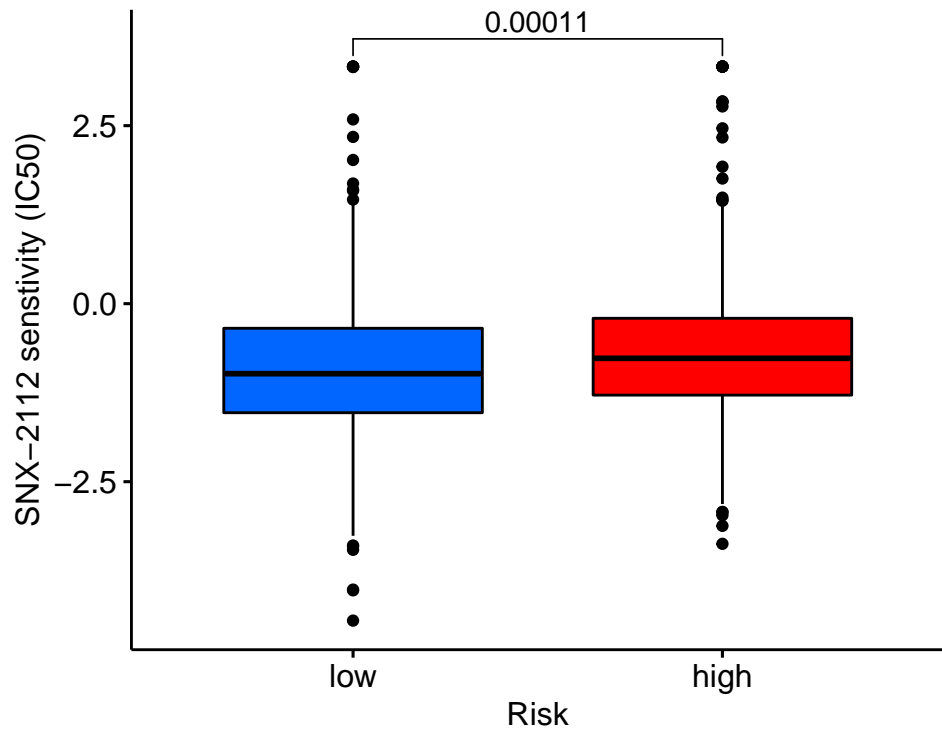

Supplement: Supplementary file 1 [file diagnostics-13-01203-s001.zip › Figure S3/durgSenstivity.SNX-2112.pdf]

Risk 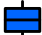 low 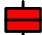 high

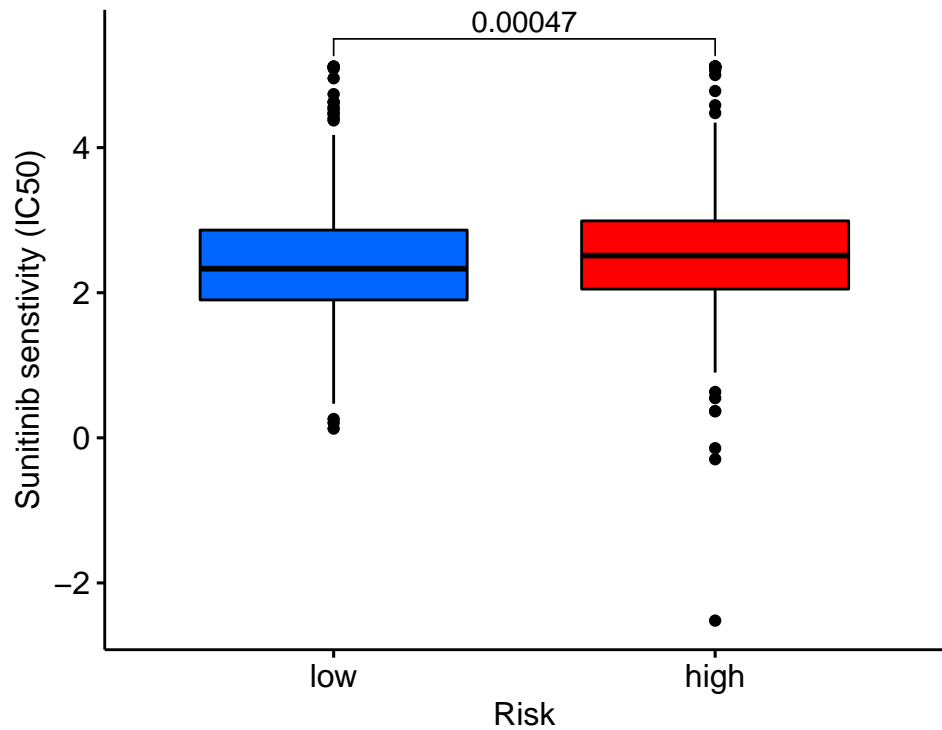

Supplement: Supplementary file 1 [file diagnostics-13-01203-s001.zip › Figure S3/durgSenstivity.Sunitinib.pdf]

Risk 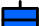 low 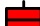 high

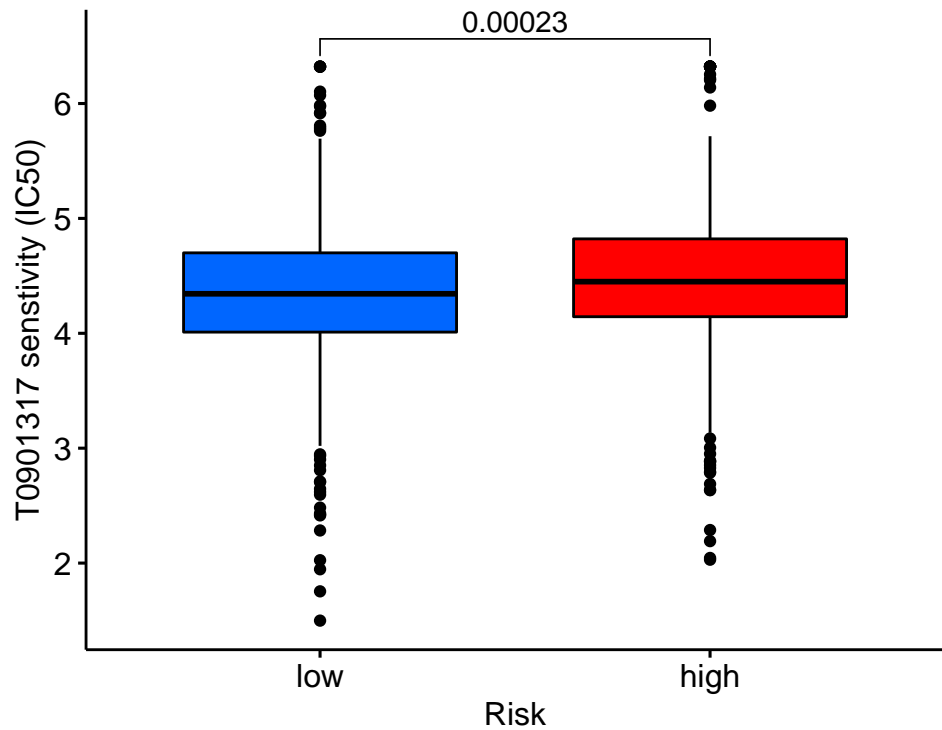

Supplement: Supplementary file 1 [file diagnostics-13-01203-s001.zip › Figure S3/durgSenstivity.T0901317.pdf]

Risk 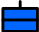 low 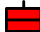 high

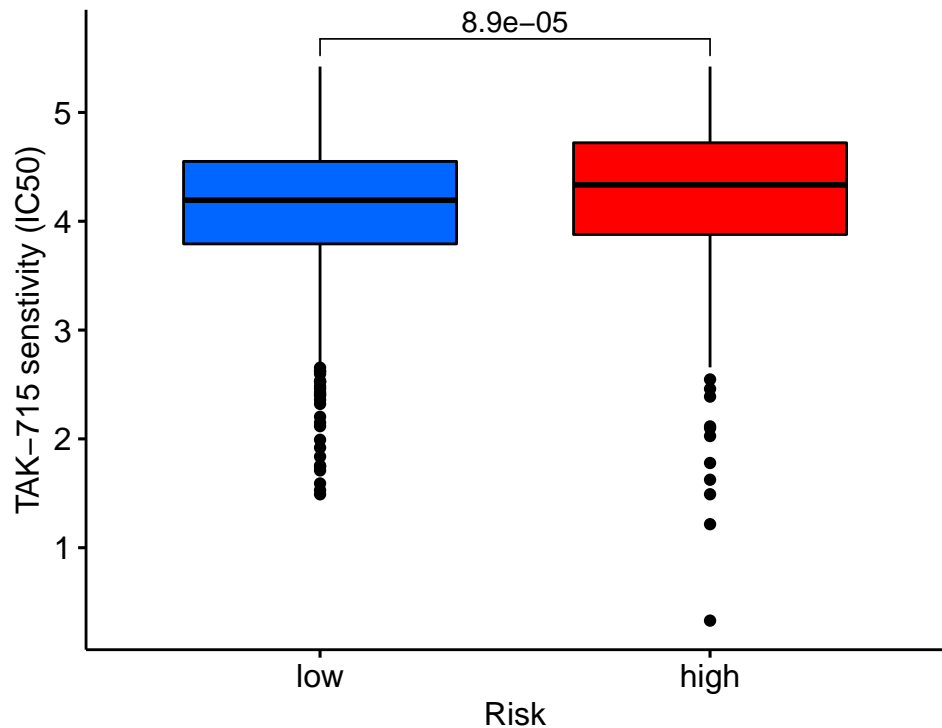

Supplement: Supplementary file 1 [file diagnostics-13-01203-s001.zip › Figure S3/durgSenstivity.TAK-715.pdf]

Risk 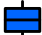 low 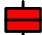 high

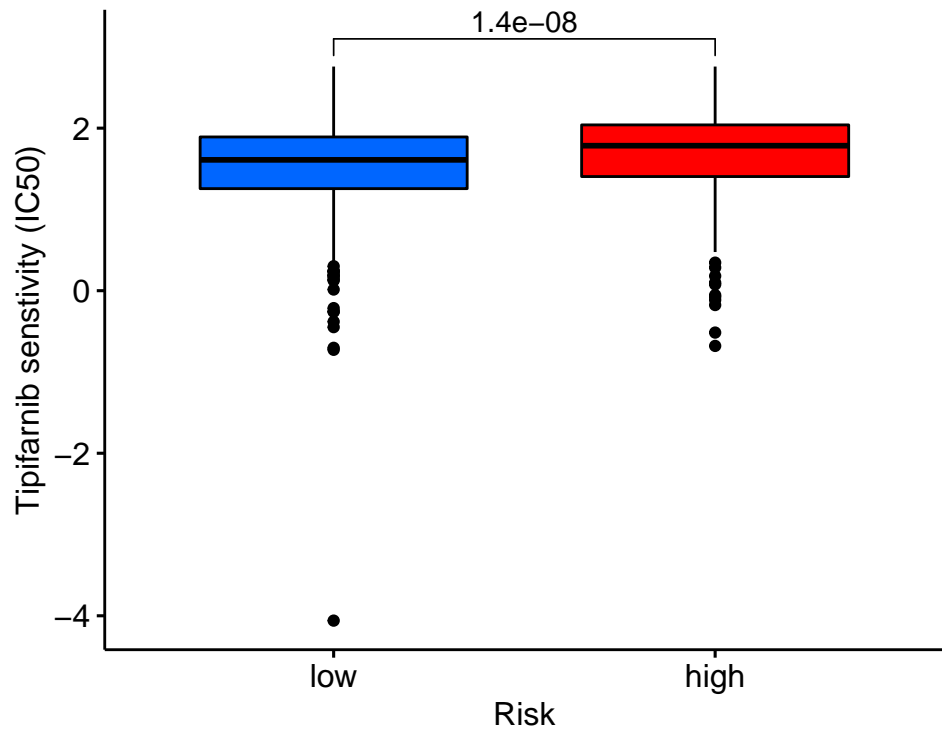

Supplement: Supplementary file 1 [file diagnostics-13-01203-s001.zip › Figure S3/durgSenstivity.Tipifarnib.pdf]

Risk 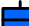 low 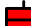 high

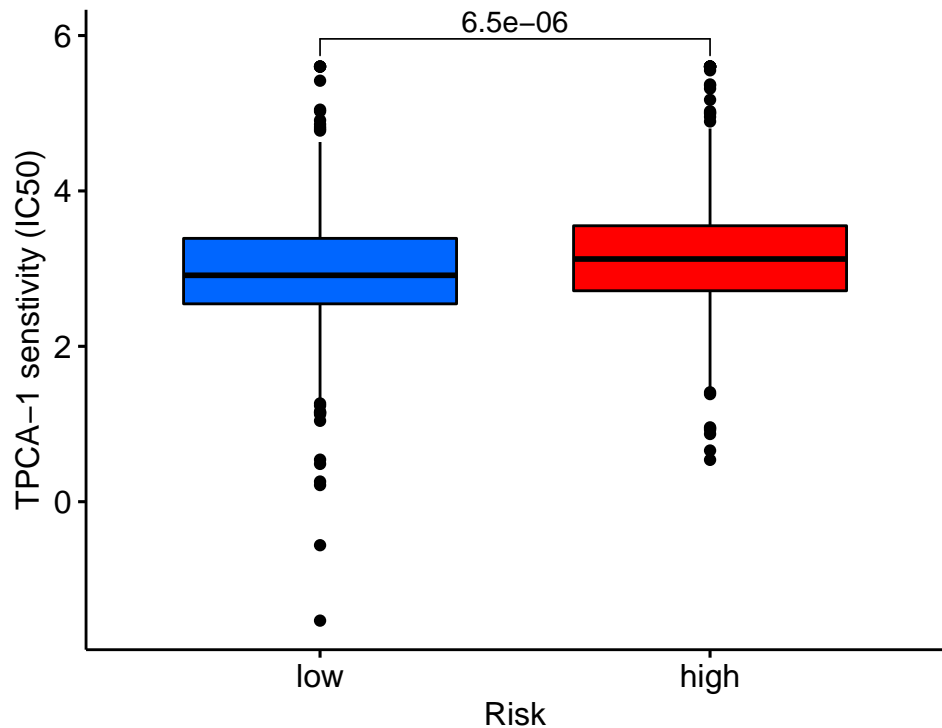

Supplement: Supplementary file 1 [file diagnostics-13-01203-s001.zip › Figure S3/durgSenstivity.TPCA-1.pdf]

Risk 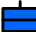 low 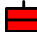 high

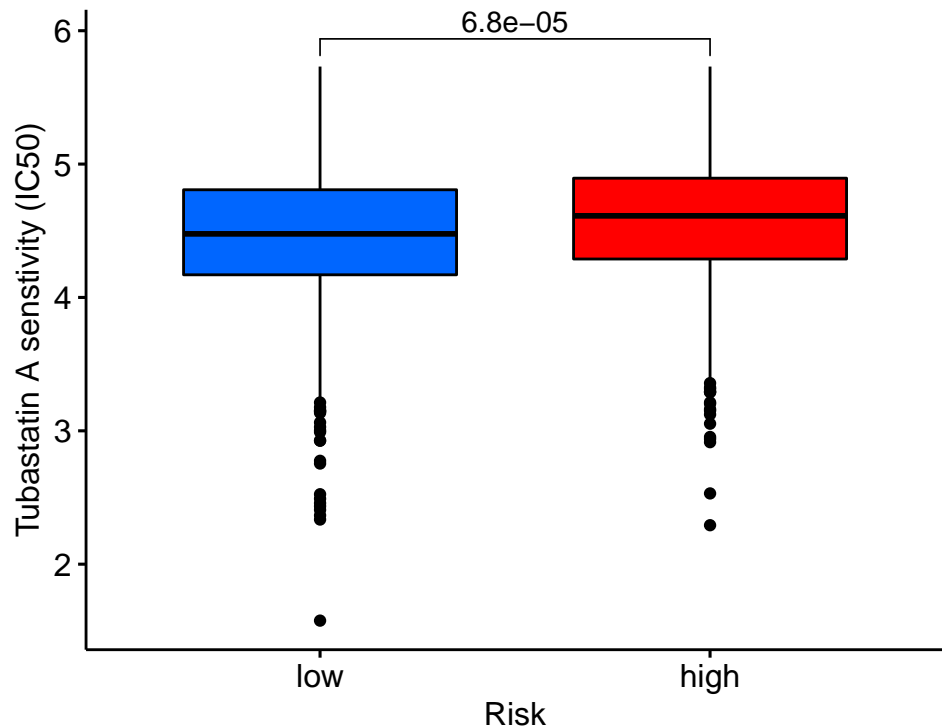

Supplement: Supplementary file 1 [file diagnostics-13-01203-s001.zip › Figure S3/durgSenstivity.Tubastatin A.pdf]

Risk 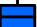 low 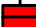 high

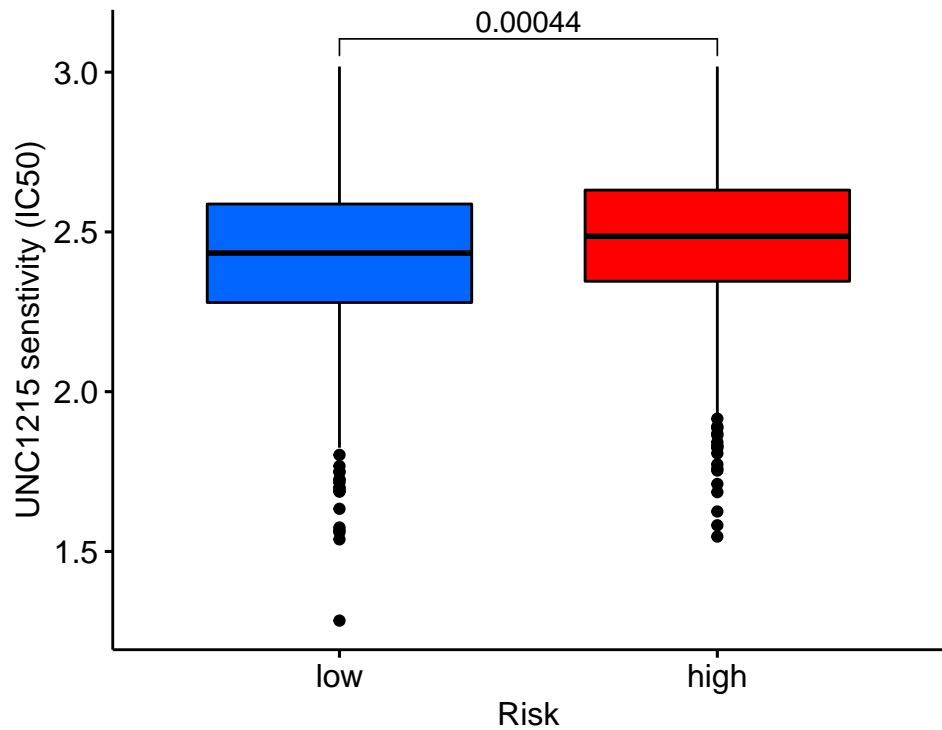

Supplement: Supplementary file 1 [file diagnostics-13-01203-s001.zip › Figure S3/durgSenstivity.UNC1215.pdf]

Risk 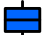 low 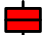 high

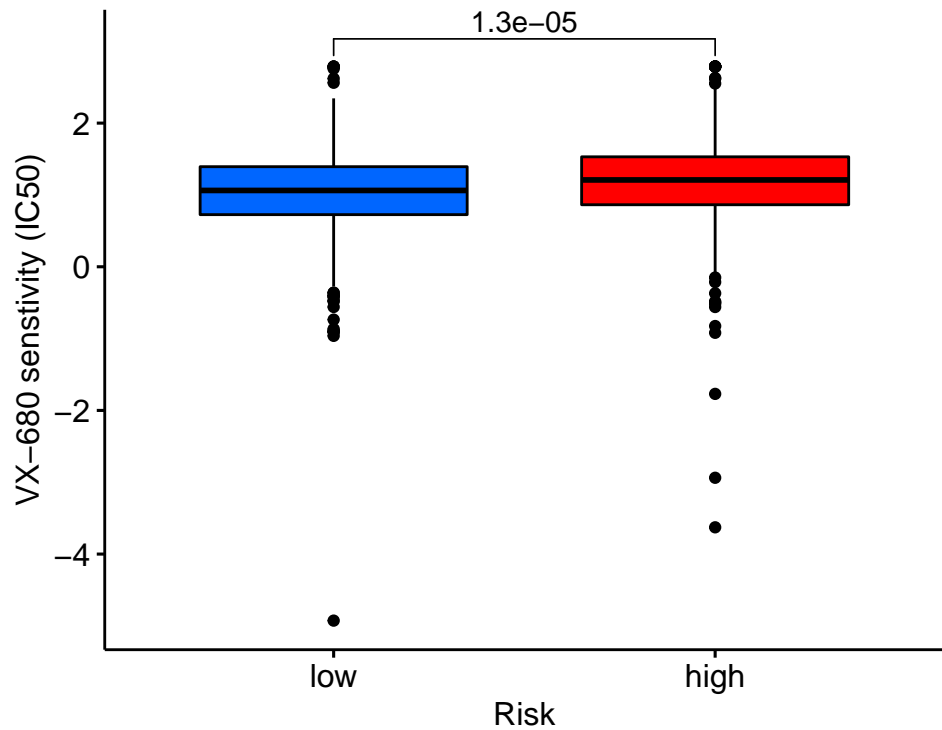

Supplement: Supplementary file 1 [file diagnostics-13-01203-s001.zip › Figure S3/durgSenstivity.VX-680.pdf]

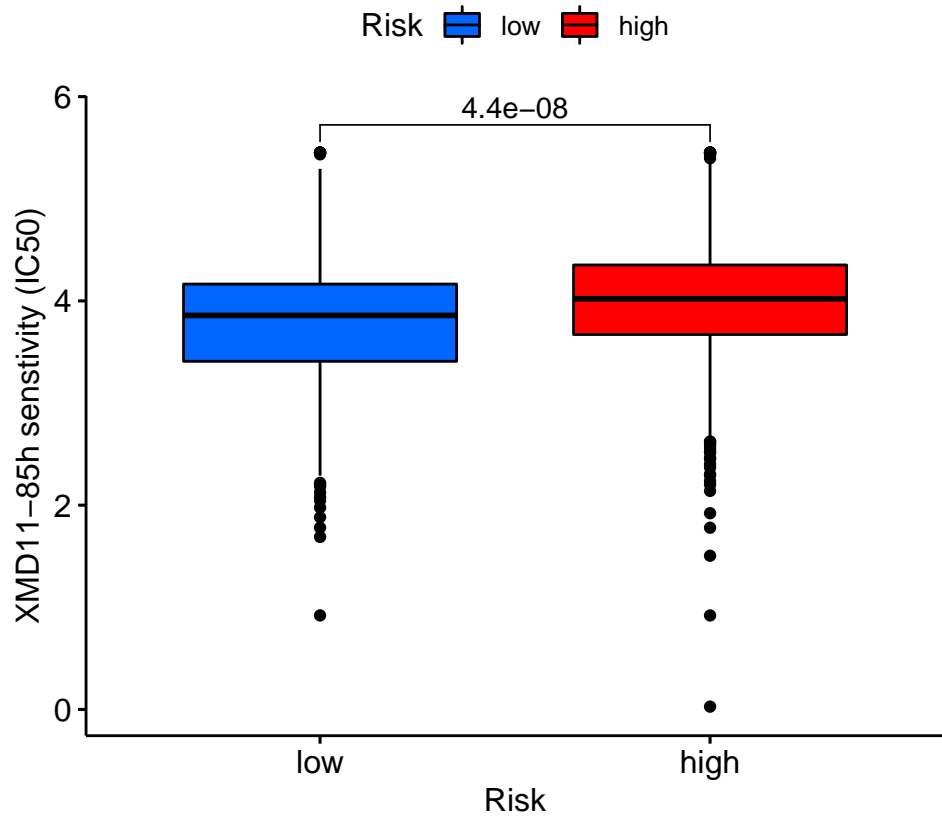

Supplement: Supplementary file 1 [file diagnostics-13-01203-s001.zip › Figure S3/durgSenstivity.XMD11-85h.pdf]

XMD8-92 sensitivity (IC50)

Risk 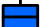 low 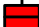 high

$5.9e-05$

10.0

7.5

5.0

2.5

0.0

low

high

Risk

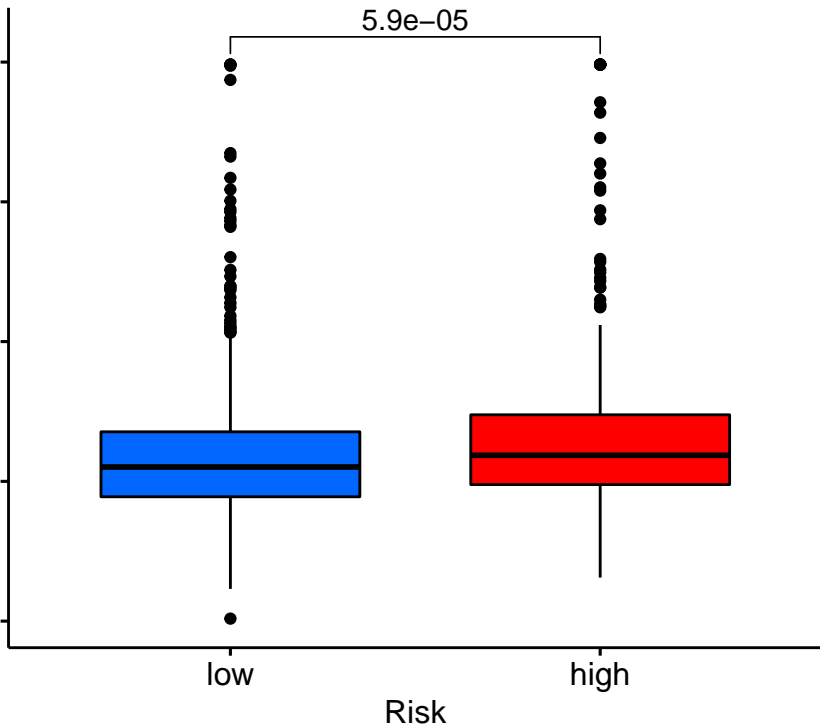

Supplement: Supplementary file 1 [file diagnostics-13-01203-s001.zip › Figure S3/durgSenstivity.XMD8-92.pdf]

Risk 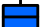 low 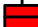 high

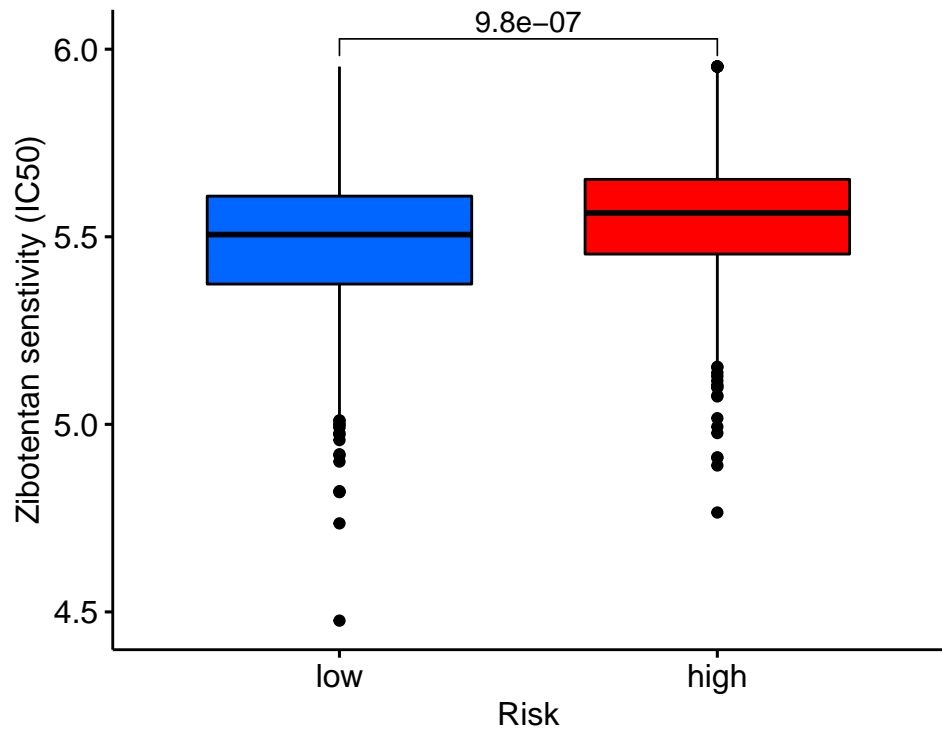

Supplement: Supplementary file 1 [file diagnostics-13-01203-s001.zip › Figure S3/durgSenstivity.Zibotentan.pdf]

Risk 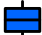 low 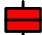 high

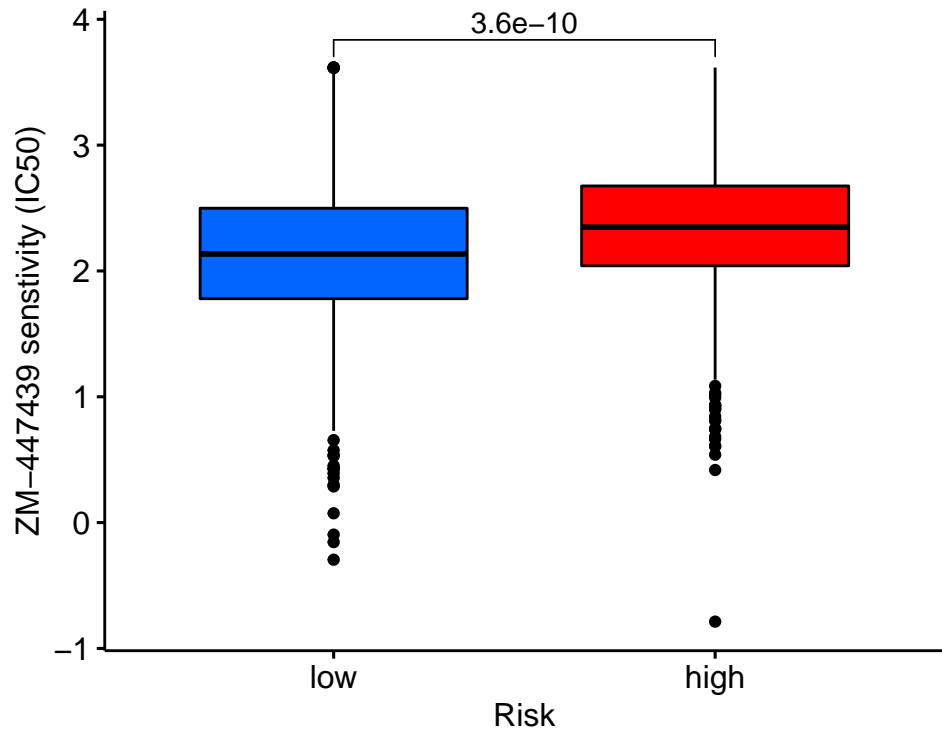

Supplement: Supplementary file 1 [file diagnostics-13-01203-s001.zip › Figure S3/durgSenstivity.ZM-447439.pdf]
