# Supplementary material for: Construct ceRNA Network and Risk Model of Breast Cancer Using Machine Learning Methods under the Mechanism of Cuproptosis
Source: Diagnostics (Basel). 2023 Mar 22;13(6):1203. doi: 10.3390/diagnostics13061203 (PMC10047351; doi:10.3390/diagnostics13061203)
Supplement: Supplementary file 1 [file durgSenstivity.681640.pdf › Figure S3/durgSenstivity.681640.pdf]

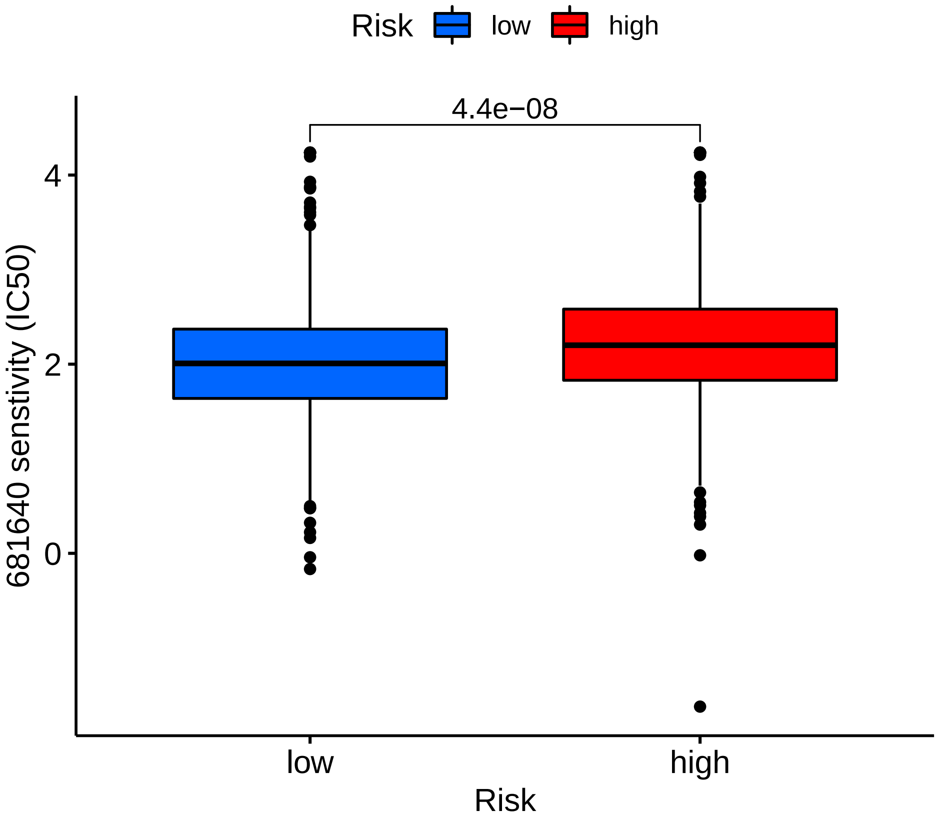

Risk
low
high
4.4e−08
4
2
0
681640 senstivity (IC50)
low
high
Risk
