# Supplementary material for: Construct ceRNA Network and Risk Model of Breast Cancer Using Machine Learning Methods under the Mechanism of Cuproptosis
Source: Diagnostics (Basel). 2023 Mar 22;13(6):1203. doi: 10.3390/diagnostics13061203 (PMC10047351; doi:10.3390/diagnostics13061203)
Supplement: Supplementary file 1 [file durgSenstivity.VX-11e.pdf › Figure S3/durgSenstivity.VX-11e.pdf]

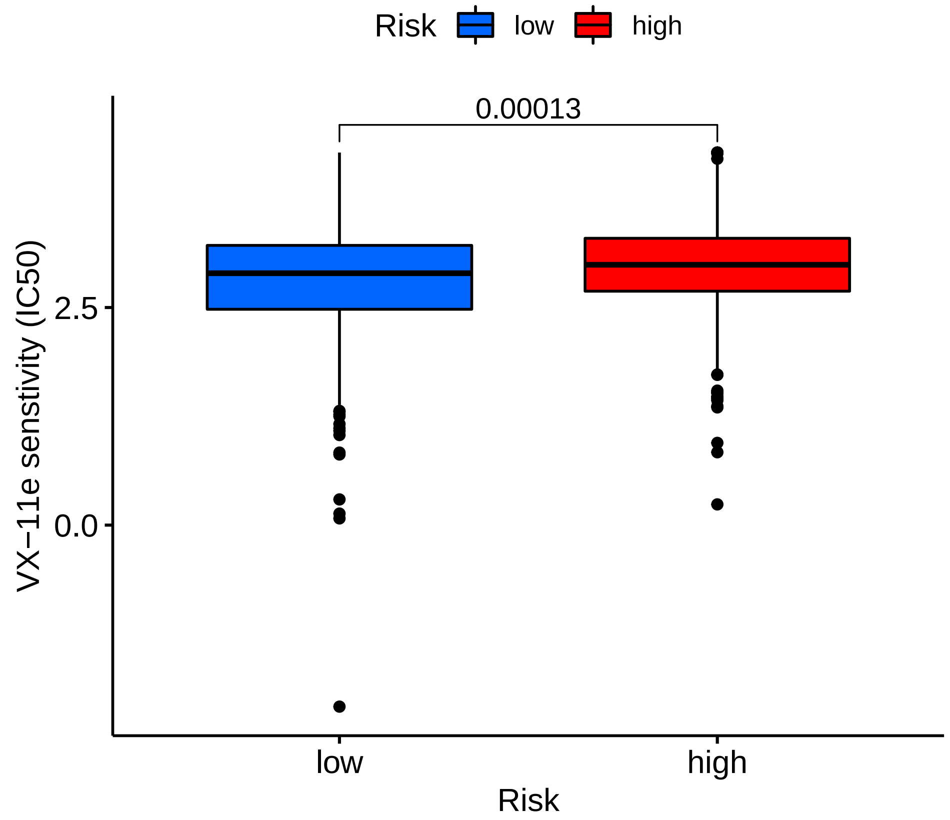

Risk
low
high
0.00013
VX−11e senstivity (IC50)
2.5
0.0
low
high
Risk
